# *Th*IPK1 regulates lignocellulolytic enzyme expression during wood degradation in white-rot fungi

**DOI:** 10.1128/mbio.01243-25

**Published:** 2025-08-18

**Authors:** Xinlei Zhang, Rong Zhu, Diao Yin, Chengkai Wang, Shenglong Liu, Ursula Kües, Rong Jia, Yazhong Xiao, Zemin Fang, Juanjuan Liu

**Affiliations:** 1 School of Life Sciences, Anhui University 428675 , Hefei, Anhui, China; 2 Anhui Key Laboratory of Biocatalysis and Modern Biomanufacturing, Hefei, Anhui, China; 3 Anhui Provincial Engineering Technology Research Center of Microorganisms and Biocatalysis, Hefei, Anhui, China; 4 Büsgen-Institut, Molecular Wood Biotechnology and Technical Mycology and Goettingen Center for Molecular Biosciences (GZMB), University of Goettingen 163282, Göttingen, Germany; Friedrich-Schiller-Universitat, Jena, Germany

**Keywords:** wood degradation, lignocellulolytic enzyme, inositol polyphosphate, IPK1, Zn_2_Cys_6 _transcription factor

## Abstract

White-rot fungi play a crucial role in terrestrial carbon cycling by decomposing lignocellulose, particularly lignin, in plant cell walls. The degradation process is initiated by the fungal perception of lignocellulosic signals, which trigger a complex regulatory network controlling lignocellulolytic enzyme expression. However, the ecological and molecular mechanisms underlying how these fungi sense lignocellulosic signals and regulate their degradation capacity remain unclear. In this study, a degenerated *Trametes hirsuta* AH28-2 that lost lignocellulolytic degradation capacity was generated through successive subcultures. Phenotypic stability, transcriptomic analyses, and functional validation revealed that *Th*IPK1, a key gene in the inositol polyphosphate pathway, regulates the orchestrated expression of lignocellulolytic enzymes in response to distinct lignin monomers. Epigenetic modifications, particularly 5mC methylation, were identified as mediators of signal transduction and regulation for *Th*IPK1 and its downstream Zn_2_Cys_6_ transcription factors, which differentially control lignocellulolytic gene transcription. IPK1 exhibits a closer relationship among white-rot fungi compared to their phylogenetic relationships. These findings provide novel insights into the molecular basis of white-rot fungal adaptation to lignocellulosic environments, contributing to our understanding of microbial-driven lignin turnover in forest ecosystems.

**IMPORTANCE:**

White-rot fungi are among the most efficient lignocellulose degraders in nature. Understanding how white-rot fungi sense and respond to lignocellulose is critical for deciphering microbial contributions to forest carbon turnover. Despite their ecological importance, the molecular mechanisms underlying lignin signal perception remain elusive. In this study, we uncover a regulatory axis involving inositol polyphosphate signaling and epigenetic modulation that connects environmental lignin cues to the transcriptional control of lignocellulolytic enzymes. By identifying *Th*IPK1 as a crucial regulator and revealing 5mC methylation and Zn_2_Cys_6_ transcription factors as downstream effectors, we demonstrate how fungi integrate chemical signals from lignin monomers into adaptive gene expression. These findings not only reveal a novel lignin-responsive regulatory mechanism but also provide a framework for understanding fungal adaptation and function in dynamic, lignin-rich environments.

## INTRODUCTION

Lignocellulose, composed of cellulose, hemicellulose, and lignin, is the most abundant source of organic carbon in terrestrial ecosystems. Lignin, a complex and heterogeneous aromatic polymer, provides structural rigidity to plant cell walls, making them highly recalcitrant to degradation (1). In forest ecosystems, white-rot fungi, primarily basidiomycetes, are the key decomposers capable of mineralizing lignin, playing a pivotal role in carbon cycling and organic matter turnover (2). Their lignocellulolytic enzyme systems, including oxidoreductases, such as lignin peroxidase (LiP), manganese peroxidase (MnP), versatile peroxidase (VP), and laccase (Lac), as well as hydrolytic enzymes targeting cellulose and hemicellulose, facilitate the breakdown of plant biomass into bioavailable carbon sources (3). Effective lignin degradation is not only essential for nutrient recycling but also influences soil carbon sequestration and greenhouse gas emissions (4).

The wood-decaying white-rot fungi that harbor most basidiomycetes or certain types of ascomycetes are the most effective microorganisms with the ability to completely degrade lignocellulose, especially lignin into its monomeric constituents (5, 6). Although lignocellulose varies considerably in type, relative amount, and structure depending on plant species, age, and growing season, many white-rot fungi are able to dominate and colonize the lignocellulosic matrix by adapting their lignocellulosic enzymes. According to recent transcriptomic or proteomic studies on tailored responses of different fungi to various compositions of plant biomass (5, 7–9), significant variations have been observed between their secreted lignocellulosic enzymes, even in closely related white-rot fungal species. However, the mechanism by which the white-rot fungi sense the lignocellulosic signals and further regulate downstream lignocellulosic enzyme expression remains unclear.

Extensive studies in ascomycetes have shown that cellulolytic enzymes can be induced by the end product of cellulose (such as cellobiose) (10, 11). This regulatory mechanism enables microorganisms to acquire carbon sources more efficiently in complex environments while optimizing their metabolic energy allocation (12). The Pth11-like G protein-coupled receptors and the serine-arginine protein kinase SrpkF have been identified as being involved in environmental sensing and the regulation of cellulolytic enzymes in fungi such as *Trichoderma reesei* (13, 14). However, the regulation of lignin-degrading enzymes in basidiomycetes, particularly their response to complex lignocellulosic substrates, remains largely unexplored. Lignin and certain lignin monomeric compounds are considered to regulate the expression of lignocellulolytic enzyme gene (15, 16). Limited evidence suggests that intracellular signaling molecules, such as cAMP, play a role in regulating the expression of lignin-degrading enzymes in response to lignin monomeric compounds (17, 18). Additionally, some transcription factors, such as Zn_2_Cys_6_ zinc finger proteins, have been implicated in lignin degradation in *Pleurotus ostreatus* (19, 20). These findings suggest that white-rot fungi possess complex regulatory mechanisms that optimize enzyme production based on substrate availability.

Despite these insights, a major gap remains in understanding how white-rot fungi sense and respond to the diverse lignocellulosic substrates they encounter in different environments. Forests, grasslands, and wetland ecosystems contain highly variable plant biomass compositions, requiring fungi to adjust their enzymatic strategies accordingly (21–23). Recent transcriptomic and proteomic analyses have revealed species-specific and even strain-specific responses to different plant materials (24–26). Such adaptability suggests that white-rot fungi dynamically regulate lignocellulolytic enzyme expression in response to their ecological niche. Investigating these regulatory pathways not only advances our understanding of fungal metabolism but also provides insight into their broader role in shaping terrestrial carbon cycling.

Inositol polyphosphates (InsPs) are a crucial class of signaling molecules derived from inositol, and they are enzymatically phosphorylated by a variety of inositol polyphosphate kinases to generate highly phosphorylated forms (27). The specific number and position of phosphates on the inositol ring confer InsPs with different structures and distinct physiological functions. Among them, inositol hexakisphosphate (InsP_6_) and inositol pyrophosphate (PP-InsPs), which have been revealed to participate in multiple cellular activities of yeasts and *Cryptococcus neoformans*, have drawn increasing attention for their function in fungi (28, 29). InsP_6_ is catalyzed by 1,3,4,5,6-pentakisphosphate-2-kinase (IPK1). This kinase plays a conservative role in the response to phosphate deficiency in both plants and fungi (30). Furthermore, because IPK1 is associated with nuclear mRNA export and polarized cell growth in *Schizosaccharomyces pombe* (31), and its knockout leads to disrupted mitochondrial function and pathogenicity in *Candida albicans* (32), IPK1 and InsP_6_ are supposed to be essential in different fungal cellular processes. The further downstream PP-InsPs have recently been demonstrated to act as important signal molecules involved in phosphate signaling machinery, stabilizing pathogenicity, and balancing glycolysis and respiration (28, 29, 33, 34). However, the function of IPK1 and InsPs in white-rot fungi has not been elucidated.

*Trametes* species are a unique genus that is widespread and acts as a great carbon recycler in forest ecosystems (35). *Trametes hirsuta* AH28-2 harbors at least 29 ligninolytic enzyme genes, including 12 LiPs, 6 MnPs, 5 VPs, and 6 Lacs, and at least 30 CAZyme genes (36). In this study, we conducted a systematic analysis of the strain morphology and physiological changes, as well as signaling pathway discrepancies, between *T. hirsuta* AH28-2 cultures grown in the absence of lignin signals and with exposure to lignin monomers. We identified that the InsPs pathway can differentially respond to distinct types of lignin signals. The downstream Zn_2_Cys_6_ transcription factors coordinately regulated the expression of lignocellulosic enzymes to enable wood degradation in white-rot fungi. Furthermore, the fungal DNMT1b contributed to the activation of signal transduction in response to InsPs and lignin monomer compounds, helping the fungus better adapt to and utilize wood in its environment.

## MATERIALS AND METHODS

### Fungal strains and culture

*T. hirsuta* AH28-2 was successively subcultured on compound potato dextrose agar (cPDA; per liter, filtrate of 200 g boiled potato, 20 g glucose, 3 g KH_2_PO_4_, 1.5 g MgSO_4_·7H_2_O, 0.05 g vitamin B1, 15 g agar, and with or without 1 mM lignin monomer compounds or derivatives) plates at 28°C. Three independent *T. hirsuta* AH28-2 clones with similar phenotypic characteristics were used to perform parallel, successive subcultures. Each clone was subcultured after 9 days of culture on cPDA plates in the dark to form three independent *D1–D10* or *T1–T10* culture series (Fig. S1A). Subcultured clones were stored in 20% glycerol (vol/vol) at −80°C for use in the same batch experiments. Minimum carbon source culture medium comprising per liter: 0.1 g (NH_4_)SO_4_, 0.2 g MgSO_4_·7H_2_O, 0.2 g Na_2_HPO_4_·12H_2_O, 0.4 g KH_2_PO_4_, 0.05 g VB_1_, 1 mg FeSO_4_·7H_2_O, 1 mg CaCl_2_, with or without 1.5% agar and 10 g of different carbon sources. To improve the visualization of the fungal colonies growing on cellulose medium, the medium was supplemented with 40 mg/L Remazol Brilliant Blue R. The XH medium (per liter, 15 g cellobiose, 1 g peptone, 1.5 g DL-asparagine, 0.1 g Na_2_HPO_4_, 1 g KH_2_PO_4_, 0.5 g MgSO_4_·7H_2_O, 0.01 g CaCl_2_, 1 mg FeSO_4_·7H_2_O, 27.5 mg adenine, 0.05 mg vitamin B1, and 2 mg CuSO_4_·7H_2_O) was used for its liquid culture as previously described (37). The poplar culture medium only contains 3 g dry poplar powder per 160 mL ddH_2_O.

### Optical and scanning electron microscopy analysis

Six actively growing *T. hirsuta* AH28-2 blocks (5 mm in diameter) on the plates were inoculated into liquid XH medium and continuously shaken at 120 rpm in the dark for 4 days. After homogenization, primary cultures (5 mL) were inoculated into 160 mL of poplar medium. After culturing at 120 rpm for 5 or 40 days, some wood was harvested and observed under an optical microscope (Motic’s AE2000). After 40 days of culture, the wood samples were washed three times with PBS buffer and then dried in an oven to constant weight. The dried samples were ground and sputter-coated with gold. The surface morphology was observed using a scanning electron microscope (Hitachi S-4800).

### Lignocellulose-degrading enzyme activity assay

The activities of cellulase and xylanase were estimated using the dinitrosalicylic acid method. One unit of enzyme activity was defined as the amount of enzyme releasing 1 µM reducing sugar per hour. The activities of *β*-glucosidase were estimated using the *p*-nitrophenol matrix method. One unit of enzyme activity was defined as the amount of enzyme releasing 1 µM *p*-nitrophenol per hour. The laccase activity was determined using guaiacol as the substrate, as previously described (37). The activities of MnP and LiP were measured using Mn^2+^ ions and veratrol as substrates, respectively.

### Compositional analysis of poplar wood

Compositions of poplar wood were determined before and after degradation by *T. hirsuta* AH28-2. Lignin, cellulose, and hemicellulose concentrations of the wood samples were determined by the protocol described in the NREL report using two-stage acid hydrolysis. Briefly, 72% (vol/vol) sulfuric acid was added to the wood sample and hydrolyzed at 30°C for 1 h. Samples were diluted to 4% (vol/vol) and autoclaved for 1 h. After the hydrolyzed sample was cooled to room temperature, vacuum filtration was performed using a pre-weighed sand core funnel. The core funnel and insoluble matter were dried at 105°C until a constant weight was reached. The filtrate was used to determine acid lignin, cellulose, and hemicellulose concentrations. The concentration of acid-soluble lignin was determined by UV-visible spectrophotometry (UNICO UV-2100), and cellulose and hemicellulose contents were determined by high-performance liquid chromatography (Agilent 1260 Infinity).

### Determination of ergosterol content

Ergosterol is a good indicator of fungal biomass in solid substrates. Ergosterol was assayed by the HPLC method. Briefly, the wood and mycelium mixture samples were freeze-dried and then fully ground under liquid nitrogen. The weighed sample was resuspended in 20% (mass/vol) NaOH and saponified at 85°C for 2 h. After centrifugation, ergosterol was fully extracted with ethanol from the precipitate, and HPLC was performed to determine the concentration. For standards, ergosterol (Sigma) was dissolved in ethanol, and samples were analyzed in the same manner. Ergosterol content was expressed in micrograms per gram of dry mass of fungal wood.

### Transcriptomic analysis by RNA-Seq

Mycelia from different cultures, treated or untreated with 1 mM *o*-toluidine for 48 h, were grown in liquid culture using cellobiose as the carbon source, collected, and washed with PBS buffer. Total RNA was extracted using RNAiso Plus reagent (Takara, Japan) following the manufacturer’s instructions. Construction of libraries and sequencing with the Illumina HiSeq 2500 platform were performed by Shanghai Personal Biotechnology Co., Ltd. (Shanghai, China). The transcriptome data were analyzed based on *T. hirsuta* AH28-2 genome data (NCBI genome assembly ASM130462v1). Reads that aligned uniquely with the reference sequence were used for gene expression quantification by the reads per kilobase per million reads method. Differential expression analysis was performed with DESeq software by using cutoffs of an adjusted *P* value of 0.05 and a ≥2-fold change (Benjamini-Hochberg method). Three biological replicates were established for each treatment. The pathway enrichment analysis of differentially expressed genes (DEGs) was based on the KEGG database.

### Quantitative reverse transcription PCR analysis

The mycelia of *T. hirsuta* AH28-2 cultured in the liquid medium were withdrawn at the indicated times for total RNA extraction. Then, 1 µg of total RNA was used as the template for cDNA synthesis following the instructions of the PrimeScript RT kit (TaKaRa, Dalian, China). Quantitative reverse transcription PCR (qRT-PCR) was performed to analyze the transcript levels of target genes using an SYBR Green kit (TaKaRa) on a Roche LightCycler 96 Real-Time PCR System (Roche, Basel, Switzerland). The gene *gapdh* was used as a reference gene to normalize the qRT-PCR data. The 2*^-△△CT^* method was used to calculate the relative expression levels of each gene. Primer sequences are shown in Table S1.

### Intracellular ROS and ATP concentration quantification

After being grown for a specific time, the mycelium in the liquid medium was harvested and washed twice with PBS buffer. The cells were ground with glass beads, and the supernatant was collected by centrifugation. Intracellular ROS concentrations were measured using the fluorogenic probe DCFH-DA (Beyotime Biotech, Shanghai, China). Cytoplasmic ATP content was measured with the ATP detection kit (Beyotime Biotech, Shanghai, China).

### Annexin V and PI double staining

Cell apoptosis was assayed using an Annexin V-PI double staining kit according to the manufacturer’s instructions (Keygen Biotech, Nanjing, China). Briefly, mycelium was first washed three times with the PBS buffer, which was then incubated at 4°C for 20 min in 500 µL Annexin-V binding buffer to which 5 µL Annexin V-EGFP and 5 µL PI were added. The mycelium was washed three times with PBS buffer and then subjected to observation using a confocal microscope (Olympus, Tokyo, Japan) at 488 and 594 nm wavelengths.

### Purification of inositol polyphosphates by titanium dioxide pull-down

The extraction of inositol polyphosphates was performed according to Wilson et al*. *(38).* T. hirsuta* AH28-2 was grown in XH medium for 48 h with shaking at 28°C. The medium was centrifuged at 10,000 × *g* for 10 min at 4°C to harvest the mycelium. Pellets were washed once with PBS buffer and resuspended in 1 mL of perchloric acid solution (1 M perchloric acid and 5 mM EDTA). The mixture was thoroughly vortexed for 5 min at 4°C with acid-washed glass beads, followed by centrifuging at 15,000 × *g* for 5 min at 4°C. The TiO_2_ beads were pre-washed with perchloric acid solution, resuspended in 50 µL of perchloric acid solution, and added with the supernatant for incubation at 4°C for 20 min. Then, the mixture was centrifuged at 3,500 × *g* for 1 min at 4°C to harvest the TiO_2_ beads, followed by washing with 500 µL of 1 M perchloric acid solution once. After that, these beads were resuspended in 200 µL of 2.8% ammonium hydroxide and incubated for 5 min with a brief spin twice. Aliquots were concentrated by using a SpeedVac evaporator for 1–3 h at 40°C to remove ammonia.

### Purification of recombinant proteins

The full-length cDNA of *Thipk1* and Zn_2_Cys_6_ DNA-binding domain (DBD) sequence was amplified and inserted into the pET28a (+) vector at sites *Bam*HI and *Xho*I. *Escherichia coli* DH5*α* strain was used for plasmid construction, and *E. coli* BL21 (DE3) was used for protein expression. The positive clones were inoculated into LB medium with kanamycin and incubated at 220 rpm and 37°C until an OD_600_ of 0.6–0.8 was reached. Thereafter, isopropyl-*β*-D-thiogalactoside (0.1 mM final concentration) was added to the cell cultures for *Th*IPK1 and Zn_2_Cys_6_ DBD expression and incubated at 16°C for 16 h. Cells were harvested by centrifugation at 5,000 × *g* at 4°C for 10 min and lysed in cold Tris buffer (50 mM and 500 mM NaCl, pH 7.5) via sonication, followed by purification through Ni^2+^-NTA affinity chromatography (Novagen, Darmstadt, Germany). The Amicon Ultra Centrifugal Filter (Millipore) was used for protein concentration and buffer exchange with dialysis buffer (300 mM NaCl, 1 mM MgSO_4_, 1 mM DTT, 1% [vol/vol] glycerin, and 50 mM Tris–HCl at pH 7.5). The protein purity was estimated by SDS-PAGE, and the protein concentration was determined using the Bradford method.

### *Th*IPK1 activity assay

InsP_5_ (1,3,4,5,6-Inositol pentaphosphate) was purchased from Cayman Chemical Company. *Th*IPK1 kinase activity was assessed using the Kinase-Glo Max luminescent kinase assay (Beyotime Biotech, Shanghai, China) following the manufacturer’s instructions. Kinase reactions were performed in 25 µL volumes on black 96-well plates at 28°C. The reaction mixture comprised 50 mM Tris (pH 7.5), 5 mM MgCl_2_, 50 mM NaCl, and 300 µM ATP. A volume of 25 µL of Kinase-Glo reagent was added to stop the reaction, and luminescence was measured after 20 min on a SpectraMax M5 instrument (Molecular Devices, San Jose, CA, USA). The rate of product formation versus InsP_5_ concentration was plotted and fitted to the Michaelis-Menten equation using nonlinear regression to determine *K_m_* and *V*_max_ (GraphPad Software). Synthetic products were separated and examined using PAGE as described above.

### Electrophoretic mobility shift assay

The electrophoretic mobility shift assays (EMSAs) were performed as described previously with minor modifications (39). The specific probes labeled with 6-carboxyfluorescein at the 5′ end were used to amplify the ATG upstream regions from −500 to 0 bp of *GME4803*, *GME7109*, *GME507*, *GME3358*, and *GME7667*, respectively.

### Phylogenetic analysis

Accession numbers of amino acid sequences used for the IPK1 phylogenetic tree and Zn_2_Cys_6_ transcription factors family phylogenetic tree construction are listed in Table S2. The sequences were aligned using MUSCLE in MEGAX72 and trimmed using NGPhylogeny.fr.73. MEGAX (ML using 500 bootstraps) was used to construct the maximum likelihood phylogenetic tree, and iTOL v674 was used for the modification of the phylogenetic tree.

### *T. hirsuta* AH28-2 genomic DNA extraction

Excess water was removed from the mycelium of *T. hirsuta* AH28-2 using a vacuum pump. Dried mycelium was rapidly ground into powder in liquid nitrogen. The genomic DNA was extracted using the CTAB method. The integrity of genomic DNA was determined using 0.8% agarose gel electrophoresis, and its purity was determined on the basis of the OD_260_/OD_280_ ratio.

### Bisulfite library construction and sequencing of *T. hirsuta* AH28-2

A total of 5 µg high-quality genomic DNA was used for bisulfite conversion using the EZ DNA Methylation-Gold Kit (Zymo Research), according to the manufacturer’s protocol, which converts unmethylated cytosines to uracil while leaving methylated cytosines unchanged. Libraries with fragment sizes of ∼400 bp for each sample (three biological replicates, a total of nine libraries) were prepared following the manufacturer’s protocol. High-throughput sequencing was performed using the Illumina sequencing platform (BGI, Shenzhen, China), and the sequencing read length was 150 bp paired-end reads. Raw reads were trimmed using Fastp to remove adapters and low-quality bases. Clean reads were aligned to the *T. hirsuta* AH28-2 genome using Bismark with default parameters. Duplicate reads were removed, and methylation calls in CG, CHG, and CHH contexts were extracted using the Bismark methylation extractor. Only sites with ≥5-fold coverage were retained for analysis. Further analyses, including data filtering, normalization, and visualization, were conducted in R using methylKit.

## RESULTS

### The lignocellulosic enzyme expression and wood degradation ability are lost in *T. hirsuta* during successive subcultures in the absence of lignocellulose

**Fig 1:**
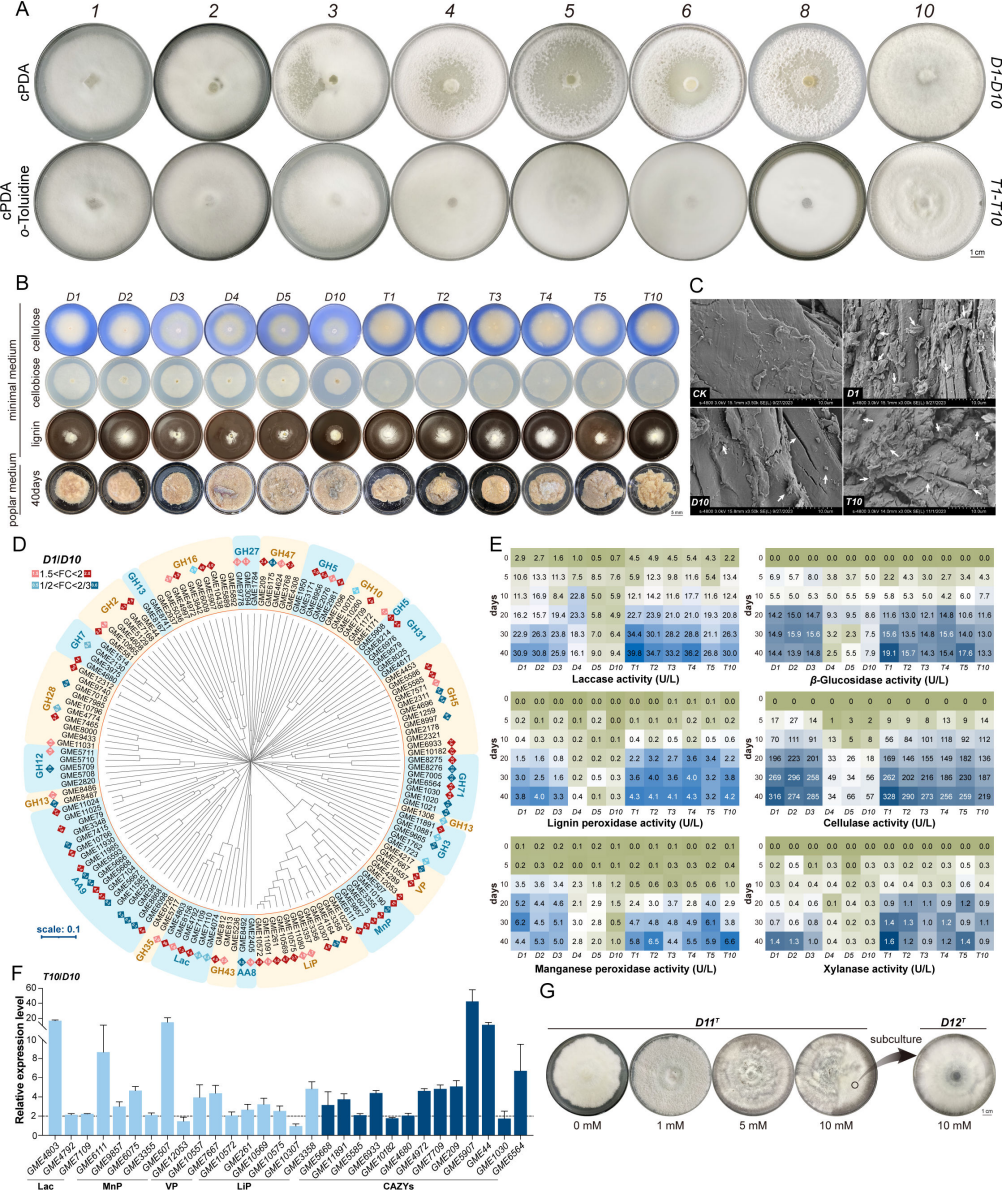
Effects of *o*-toluidine on lignocellulose degradation in *T. hirsuta* AH28-2. (**A**) The successive subcultures of *T. hirsuta* AH28-2 on cPDA medium. *T. hirsuta* AH28-2 was cultured on cPDA medium at 28°C for 9 days until the mycelium extended to the edge of the plate. A 1 cm^2^ mycelial plug was transferred to a new cPDA medium for growing. The cultures were sequentially named *D1*, *D2*, ..., and *D10* according to the number of subcultures. Similarly, these cultures successively subcultured on cPDA medium containing 1 mM *o*-toluidine were named *T1*, *T2*, ..., and *T10*. (**B**) The growth phenotypes of various cultures of *T. hirsuta* AH28-2 grown in minimal media containing only cellulose, hemicellulose, or alkaline lignin, as well as in poplar wood liquid medium. The cellulose medium was stained blue with Remazol Brilliant Blue R to enhance the visibility of the white mycelium. (**C**) Scanning electron microscope images of poplar wood degraded by various cultures of *T. hirsuta* AH28-2 after 40 days. (**D**) Phylogenetic and transcriptional level analysis of lignocellulosic enzymes in the *D1* and *D10* cultures. Red or blue squares indicate genes that are upregulated or downregulated in *D1* relative to *D10*, respectively. The numbers represent the fold change in expression. (**E**) Lignocellulosic enzyme activities of various cultures of *T. hirsuta* AH28-2 cultured in poplar wood medium at different time points. (**F**) qRT-PCR analysis showed that the transcriptional levels of lignocellulosic enzymes in *T10* were higher than those in *D10*. The selected genes are a subset of the differentially expressed genes between *D1* and *D10*. (**G**) The phenotype of *D10* culture growing in cPDA medium containing different concentrations of *o*-toluidine. *D10* was inoculated into cPDA containing 1, 5, or 10 mM *o*-toluidine and grown until hyphae touched the edge, which was called *D11^T^. D11^T^*-10 mM was subjected to another subculture called *D12^T^*.

*T. hirsuta* AH28-2 grown on polar wood was successively subcultured on cPDA plates for 10 generations (named *D1–D10*, respectively, Fig. S1A). Cultures *D3–D10* changed in colony morphology, including colony sectorization, impaired sporulation ability, and faster mycelial growth rate (Fig. 1A; Fig. S1B and C). The phenotype became more and more obvious with the passage of subcultures.

The growth rates of cultures *D1–D10* were comparable on agar plates when using xylan or glucose as carbon sources (Fig. S2A). In contrast, culture *D10* exhibited an increased capacity for utilizing starch as the sole carbon source. However, they displayed reduced abilities to grow on lignin, cellulose, and cellobiose compared to culture *D1* (Fig. 1B). When cultivated on poplar wood, cultures *D1–D3* showed a robust ability to colonize wood chips, forming a spherical structure entangled by hyphae (Fig. 1B; Fig. S2B) wrapped around the wood chips at 5 days post-inoculation (dpi) (Fig. S3A). However, the mycelia of cultures *D5–D10* only aggregated into clusters, forming pellets in between the wood chips (Fig. S2B and S3A). On 10 dpi, cultures from *D4* to *D10* exhibited gradual decline and mycelial dissolution. Based on microscope analysis and ergosterol content (Fig. 1B; Fig. S3A and B), culture *D10* almost died by 40 dpi. Wood surfaces in *D1–D3* displayed numerous random cracks on 40 dpi, while those inoculated in *D4–D10* remained smooth (Fig. 1C; Fig. S4A). In comparison to non-inoculated wood, culture *D1*-treated woods experienced reductions in lignin, cellulose, and hemicellulose contents by 10.81% ± 1.31%, 16.73% ± 1.62%, and 7.39% ± 1.10% after 40 dpi, respectively. However, minimal changes were observed in wood chips treated with culture *D10* (Fig. S4B).

Transcriptome analysis between *D1* and *D10* revealed that the expression of genes for lignocellulosic enzyme in culture *D1* was predominantly upregulated compared to culture *D10* (Fig. S5A). Specifically, six Lip, five MnP, three Lac, and three VP genes exhibited more than a twofold increase in expression (Fig. 1D; Fig. S5B; Table S2). For example, *lacA* (GME4803) and *lacB* (GME7109), previously reported to respond to aromatic monomers such as *o*-toluidine and guaiacol in *T. hirsuta* AH28-2 (9, 39), were upregulated by 86.28- and 4.84-fold, respectively (Fig. S5B). Additionally, at least 32 putative genes involved in lignocellulosic degradation pathways showed increased transcriptional levels, including seven AA9 (IPMO), three GH2 (GAL), one GH3 (BGL), eight GH5 (EG), one GH7 (CBH), and two GH47 (MAN) (Fig. 1D; Fig. S5A; Table S2). Representative lignocellulosic enzymes, including Lac, LiP, MnP, BGL, total cellulase, and xylanase, were chosen for activity determination in the supernatant of wood chip cultured *D1–D10*. At 40 dpi, the activities of Lac (30.92 ± 0.95 vs 9.40 ± 0.72 U L^−1^), LiP (3.76 ± 0.36 vs 0.25 ± 0.03 U L^−1^), and MnP (4.40 ± 0.26 vs 0.99 ± 0.21 U L^−1^) decreased dramatically in cultures *D1* and *D10*. The activities of BGL, cellulase, and XYL also exhibited significant downregulation in cultures *D4–D10* (Fig. 1E).

### Lignin monomer exposure maintains lignocellulosic enzyme secretion and wood degradation capacity during successive subcultures

Some lignin monomers (G-type: guaiacol, vanillic acid; S-type: syringic acid) and derivatives (*o*-toluidine) capable of inducing the expression of lignocellulosic enzymes in some white-rot fungi (9) were incorporated into the cPDA medium to assess their role in signal maintenance and effects on wood degradation capacity. Notably, *o*-toluidine at a final concentration of 1 mM effectively mitigated the decline in the ability of *D10* to degrade lignocellulose. *T. hirsuta* AH28-2 subcultured on cPDA plates with 1 mM *o*-toluidine for 10 generations (named *T1–T10*, respectively). The morphology of the cultures remained stable throughout subculturing. According to the growth profiles of strains on different carbon sources and poplar wood chips, along with scanning electron microscope (SEM) and wood component analyses, cultures *T1–T10* exhibited minor variations in both wood degradation capability and sporulation potential (Fig. 1A through C; Fig. S1 to S4). Their mycelia successfully colonized and wrapped the wood, resulting in irregular cracks appearing on its surface (Fig. 1C). However, unlike *D10*, the contents of lignin, cellulose, and hemicellulose in wood treated with culture *T10* decreased by 8.46% ± 1.05%, 6.71% ± 0.46%, and 3.21% ± 0.80%, respectively (Fig. S4B). The expressional and transcriptional levels of Lac, MnP, VP, LiP, and CAZymes remained consistent across cultures *T1–T10* (Fig. 1E and F). Interestingly, the compounds used above have been shown to restore the degenerated phenotype observed in *D10*, with *o*-toluidine having the best effect. When the final concentration of *o*-toluidine was elevated to 10 mM for one subculture, several degenerated phenotypic traits of culture *D10*, including colony morphology, sporulation ability, growth rate, as well as Lac and Lip synthesis, were partially restored (Fig. 1G; Fig. S6). These findings indicate that *T. hirsuta* AH28-2 exhibits a comparable response to lignin monomers or derivatives; exposure to aromatics significantly enhances signal transduction while preserving its ability for lignocellulose degradation during successive subcultures.

### Global changes at the transcriptomic level between cultures *D1*, *D10,* and *T10*

**Fig 2:**
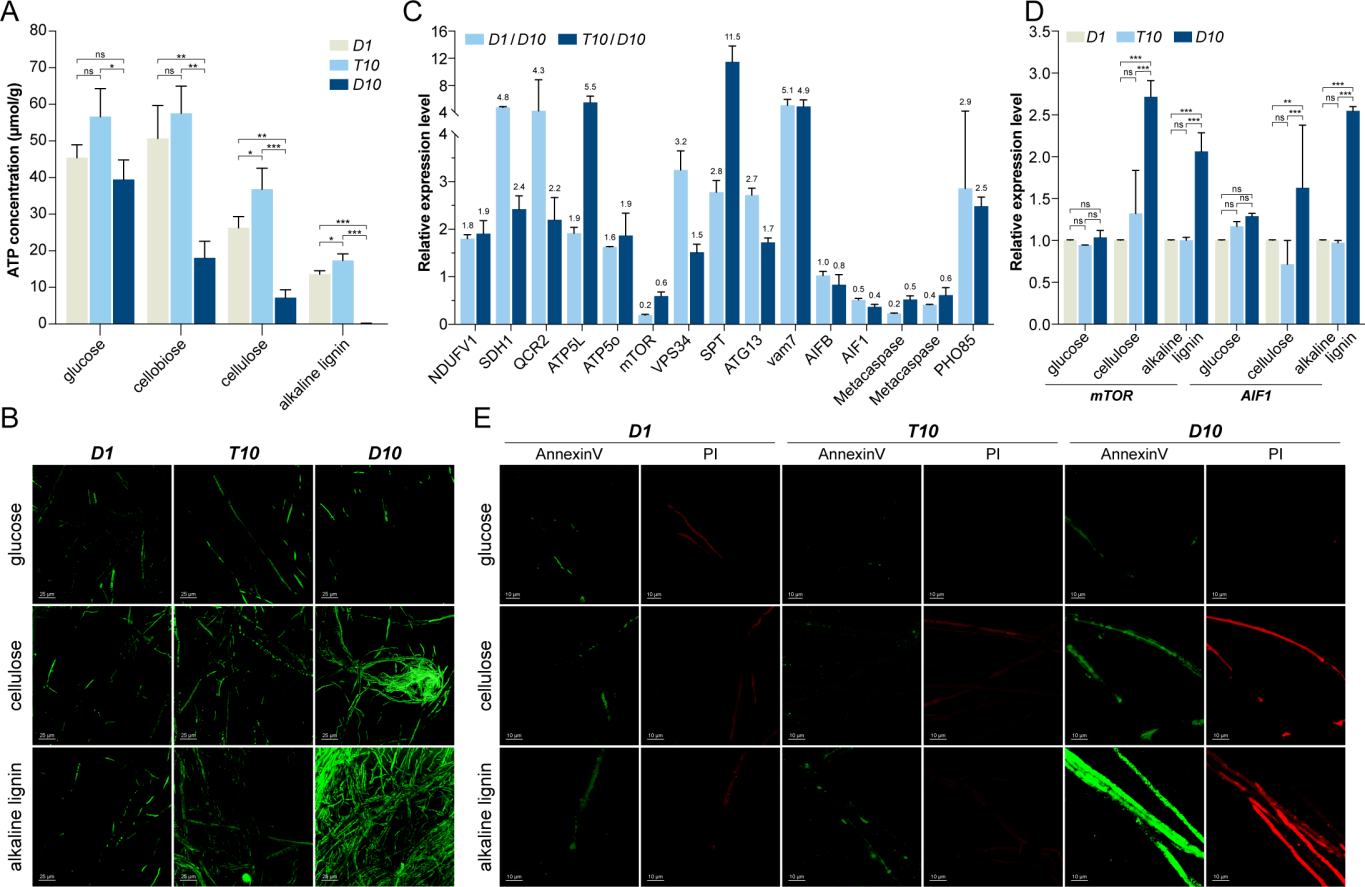
Physiological analysis of *D1*, *T10*, and *D10* cultures growing under different carbon sources. (**A**) Intracellular ATP concentrations. (**B**) Intracellular ROS levels. Scale bar, 25 µm. (**C**) The transcriptional levels of genes related to ETC, apoptosis, autophagy, and the cell cycle when using alkaline lignin as the carbon source. NDUFV1, NADH dehydrogenase (ubiquinone) flavoprotein subunits; SDH1, succinate dehydrogenase; QCR2, ubiquinol-cytochrome c reductase subunit 2; ATP5L and ATP5O, ATPase subunit; mTOR, mammalian target of rapamycin; VPS34, phosphatidylinositol 3-kinase; SPT, serine palmitoyltransferase; ATG13, autophagy-related protein; Vam7, vacuolar morphogenesis 7; AIFB and AIF1, apoptosis-inducing factor; PHO85, cyclin-dependent kinase. (**D**) The transcriptional levels of the *mTOR* and *AIF1* genes. (**E**) Apoptosis levels of *D10* cultures after 3 days of growth. Scale bar, 10 µm. Solid or liquid medium with glucose, cellulose, cellobiose, or alkaline lignin was used. ***P < 0.001; **P < 0.01; *P < 0.05; and ns, not significant.

The transcriptome of *D1* was compared with that of *D10* in order to investigate the difference between them in terms of cellular physiology and gene transcriptional profiles. A total of 1,681 genes were upregulated, and 2,061 genes were downregulated. Consistent with strain growth capability on various carbon sources (Fig. 1B), upregulated genes involved in energy metabolism, including the electron transport chain (ETC), were observed in culture *D1* (Fig. S7A; Table S3). The reduced growth rate and carbon utilization efficiency observed in *D10* may be associated with its lower intracellular ATP levels than *D1* when cultured with cellobiose, cellulose, or alkaline lignin as carbon sources (Fig. 2A; Fig. S7B and C). Furthermore, a comprehensive reduction of glycolysis and oxidative phosphorylation pathways in *D10* was indicated by the downregulation of electron transport chain-related genes and increased sensitivity to carbonyl cyanide m-chlorophenylhydrazone (a type of oxidative phosphorylation uncoupler) (Fig. S7A and D). Additionally, differences in cellulose and alkaline lignin utilization led to maintaining low concentrations of ROS in *D1* but a burst of ROS in *D10* (Fig. 2B; Fig. S8). However, the ATP and ROS stress encountered for *D10* under different carbon sources did not significantly manifest within *T10* (Fig. 2A and B; Fig. S8).

The lack of energy and high oxidative stress resulted in the downregulation of cell cycle-promoting genes, such as *pho85* and *cdc4,* while genes involved in cell cycle arrests, like mitotic arrest deficiency 2 (*mad2*) and checkpoint kinase 1 (*chk1*), were increased in *D10* compared to *D1* and *T10*, as exemplified by using cellobiose or alkaline lignin as the carbon source (Fig. 2C; Fig. S7A) (40–43). Furthermore, qRT-PCR analysis and Annexin V–PI staining revealed an upregulation of genes related to apoptosis and increased levels of apoptosis in *D10* when subcultured in cellulose and alkaline lignin (Fig. 2D and E; Fig. S7A and S9A; Table S3). The autophagy level beneficial for physiological stabilization (44) was downregulated in *D10*. This was demonstrated by an increased *mTOR* expression along with decreased *atg13* and *vps34* transcripts (Fig. 2D; Table S3). The transcriptional levels of genes involved in energy sustain, cell cycle regulation, and apoptosis showed similar patterns between *T10* and *D1* subcultures (Fig. 2C and D). These results, in consequence, not only explain the difference between *D1* vs *D10* but also shed light on why *D1* or *T10* exhibited good growth while *D10* partially died after prolonged culture with a single carbon source medium or the wood chip substrate (Fig. S9B).

### *Th*IPK1 functions as an inositol-pentakisphosphate 2-kinase in response to lignin monomers and derivatives

**Fig 3:**
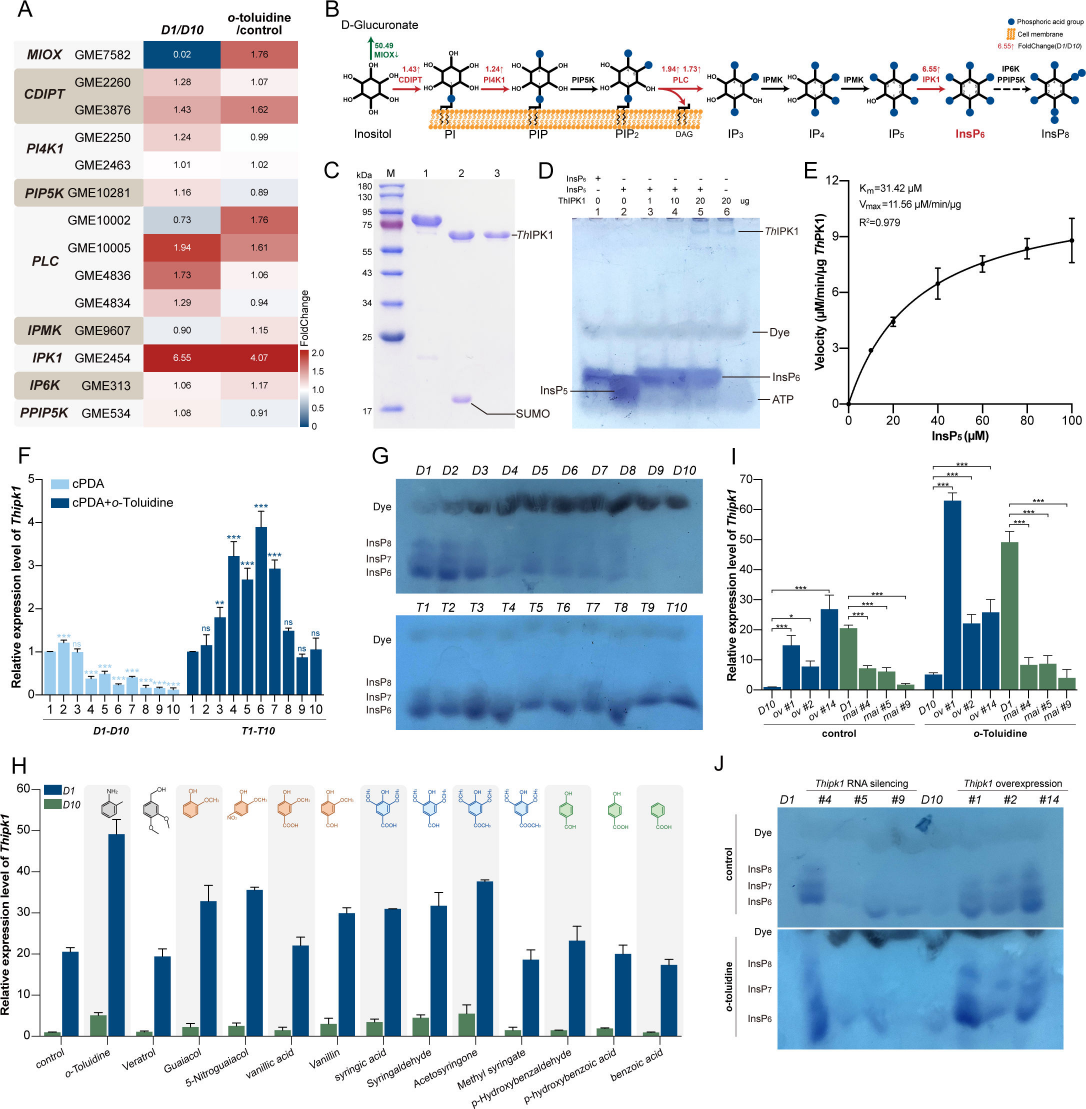
*Th*IPK1 responds to lignin monomer compounds and derivatives. (**A**) The fold change in transcription levels of genes related to the inositol polyphosphate pathway in groups *D1* vs *D10* and *o*-toluidine vs control (culture *D1* with or without *o*-toluidine treatment). (**B**) Cartoon diagram of the inositol polyphosphate biosynthetic pathways of *T. hirsuta* AH28-2, with spheres representing phosphate groups. Red or green texts indicate the gene names and fold changes of upregulated or downregulated genes in *D1* relative to *D10*, respectively. (**C**) Heterologous expression and purification of *Th*IPK1. Lane M represents the protein marker; lanes 1–3 represent purified SUMO-*Th*IPK1, the product of SUMO-*Th*IPK1 digested by ULP1, and purified *Th*IPK1, respectively. (**D**) PAGE characterization of the products catalyzed by *Th*IPK1. Lane 1 is the InsP_6_ standard; lanes 2–5 represent the products of InsP_5_ reacting with varying amounts of *Th*IPK1 for 10 min; lane 6 is *Th*IPK1 without InsP_5_. (**E**) The enzyme kinetics of *Th*IPK1 were determined by nonlinear regression according to the Michaelis-Menten model with InsP_5_ as substrate. (**F**) Transcriptional levels of *Thipk1* in various cultures of *T. hirsuta* AH28-2. (**G**) Characterization of intracellular InsP_6_ and PP-InsPs contents in various cultures of *T. hirsuta* AH28-2. (**H**) Transcriptional levels of *Thipk1* in *D1* and *D10* cultures after treatment with various lignin monomer compounds and derivatives for 48 h. (**I**) Verification of *Thipk1* transcriptional levels in *Thipk1*-silenced (or -overexpressed) transformants of culture *D1* (or *D10*). (**J**) Characterization of intracellular InsP_6_ and PP-InsPs contents in *Thipk1*-silenced and -overexpressed *T. hirsuta* AH28-2 transformants. *D1* or *T1* was set as a control in panels **F** and **I**, and one-way ANOVA tests with uncorrected Fisher’s LSD *post hoc* tests show the individual *P* values (***P < 0.001; **P < 0.01; *P < 0.05; and ns, not significant).

The addition of *o*-toluidine enabled the maintenance of signal sensing for lignocellulosic enzyme expression, thus culture *D1* was subjected to *o*-toluidine treatment for transcriptomic analysis (*o*-toluidine vs control group). This analysis was then comparatively combined with the transcriptome data from cultures *D1* and *D10* (*D1* vs *D10* group) to unravel the signaling pathways implicated in lignin perception. A total of 336 DEGs were identified in both groups, with 90 genes being upregulated in *D1* (Fig. S10A). KEGG enrichment analysis revealed a stronger correlation for the InsPs pathway compared to other enhanced signal transduction pathways (Pearson correlation of 0.74, Fig. S10B), indicating its significant involvement in lignin monomer response. The InsPs pathway consists of nine genes responsible for catalyzing the conversion of inositol to InsP_6_, which can further undergo phosphorylation to produce inositol pyrophosphate PP-InsP_7_ and PP-InsIP_8_ (Fig. 3A and B) (29). Among these genes, *GME2454* (referred to as *Thipk*1) was identified as the core gene that responds to *o*-toluidine. It showed significant differential expression in both groups (*D1* vs *D10* and *o*-toluidine vs control groups) with relatively high fold changes. Sequence alignment and phylogeny analysis suggested that *Th*IPK1 shared less than 31.9% sequence identity with IPK1 derived from other eukaryotes but possessed the conserved catalytic core amino acids across all species (Fig. S10C through E) (45). Biochemical characterization of heterologously expressed *Th*IPK1 in *Escherichia coli* BL21 (Fig. 3C) demonstrated its ability to catalyze the synthesis of InsP_6_ using InsP_5_ and ATP as substrates (Fig. 3D), with a *K_m_* of 31.42 µM and a *V*_max_ of 11.56 µM/min/µg (Fig. 3E), suggesting its function as an inositol-pentakisphosphate 2-kinase.

qRT-PCR analysis revealed a decrease in the transcriptional levels of *Thipk1* from *D1* to *D10*, while they remained stable in subcultures from *T1* to *T10* (Fig. 3F). Subsequently, total InsPs were extracted from the strains using the TiO_2_ method and separated by polyacrylamide gel electrophoresis (38). As expected, the concentrations of InsP_6_ in *D10* exhibited a reduction of 74% compared to *D1*, whereas they showed similarity within *T10* compared with cultures *D1* and *T1*, respectively (Fig. 3G; Fig. S10F). To verify whether *o*-toluidine and other lignin monomers and derivatives directly stimulated *Thipk1* expression, different G-type, S-type, and H-type lignin monomers were employed for treating cultures *D1* and *D10*. The transcriptional levels of *Thipk1,* as well as the contents of InsP_6_, were significantly upregulated in culture *D1* relative to culture *D10* when treated with *o*-toluidine or most of the guaiacyl and syringyl monomers but not of the *p*-phenyl monomers. This indicated differential responses of InsP_6_ signaling across various lignin monomers or derivatives (Fig. 3H; Fig. S11). Some S-type lignin monomers (syringic acid and acetosyringone) slightly increased the expression level of *Thipk1* and InsP_6_ in culture *D10* but still maintained them at a relatively low level (Fig. 3H; Fig. S11), suggesting that certain lignin monomers and derivatives can directly stimulate *Thipk1* expression.

*Thipk1-*silenced transformants of culture *D1* and the *Thipk1-*overexpressed transformants of culture *D10* were generated, and three randomly selected transformants from each group were chosen for verification by genomic PCR and qRT-PCR (Fig. 3I; Fig. S12A). Upon inoculation on phosphate-deficient or glycerophosphate plates, the growth rate of the cultures decreased after *Thipk1* silencing (Fig. S12B and C). Conversely, an opposite phenotype was observed upon *Thipk1* overexpression, suggesting that *Th*IPK1 was also involved in the response of basidiomycetes to phosphate stress, similar to *Saccharomyces cerevisiae* (29). Compared to culture *D10*, the intracellular InsP_6_ and PP-InsP_s_ concentrations increased by more than fivefold in the *Thipk1-*overexpressed transformants. However, the concentrations substantially decreased in the *Thipk1-*silenced transformants (Fig. 3J; Fig. S12D). A contrasting transcriptional profile was observed for myo-inositol oxygenase (*miox*) while phospholipase C (*plc*) exhibited a similar profile as that of *Thipk1* (Fig. S12E and F). It can be concluded that the InsPs synthesis pathway, particularly involving *Th*IPK1 and its downstream products InsP_6_ and PP-InsIP_s_, plays critical roles in *T. hirusta* AH28-2 during certain lignin monomer or derivative responses.

### *Th*IPK1 and its downstream products transmit signals to trigger the expression of lignocellulosic enzymes

**Fig 4:**
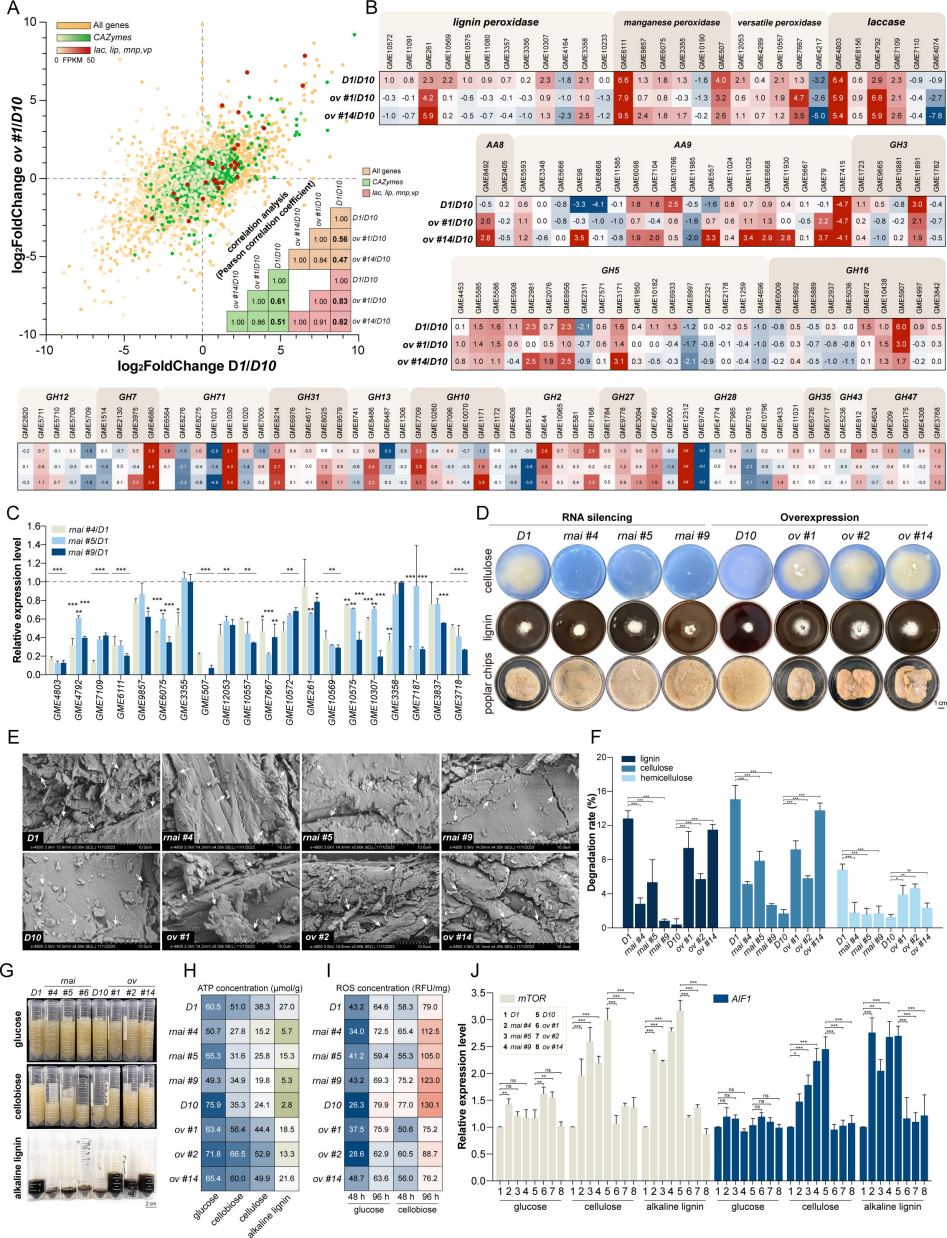
*Th*IPK1 regulates the degradation of lignocellulose by *T. hirsuta* AH28-2. (**A**) The scatter plots show the correlation of the expression fold change of all genes in *T. hirsuta* AH28-2 between *ov #1* vs *D10* (*Y*-axis) and *D1* vs *D10* (*X*-axis). The Pearson correlation coefficients of all genes, CAZyme genes, and lignin degradation enzyme genes in each of the three groups of *D1* vs *D10*, *ov #1* vs *D10,* and *ov #14* vs *D10* are marked in the triangle graphs, respectively. (**B**) The expression folds of lignocellulolytic enzyme genes. The red or green box indicates upregulated or downregulated expression in *D1*, *ov #1,* or *ov #14* compared with *D10*, respectively, and the numbers represent log_2 _fold change. (**C**) Some lignocellulolytic enzyme genes are significantly downregulated in the *Thipk1*-silenced transformants compared to *D1*. The gene of each transformant was analyzed with that of *D1* by a two-sided Student’s *t*-test (***P < 0.001; **P < 0.01; and *P < 0.05). (**D**) The growth phenotypes of *D1*, *D10*, *Thipk1*-silenced, and -overexpressed transformants grown in minimal media containing cellulose or alkaline lignin, as well as in poplar wood liquid medium. The cellulose medium was stained blue with Remazol Brilliant Blue R to enhance the visibility of the white mycelium. (**E**) SEM images of poplar wood degraded by *D1*, *D10*, *Thipk1*-silenced, and -overexpressed transformants after 40 days. (**F**) The degradation rate of lignocellulose in poplar wood after 40 days of treatment with *D1*, *D10*, *Thipk1*-silenced, and -overexpressed transformants (***P < 0.001; **P < 0.01; and *P < 0.05). The “degradation rate” indicates the reduction of lignin, cellulose, or hemicellulose after cultivating each strain. (**G–I**) Biomass (**G**), intracellular ATP concentrations (**H**), and intracellular ROS concentrations (**I**) of various *T. hirsuta* AH28-2 cultures after 7 days of growth in a liquid medium with different carbon sources. (**J**) Transcriptional levels of *mTOR* and *AIF1* genes in various *T. hirsuta* AH28-2 cultures grown in liquid medium containing different carbon sources (***P < 0.001; **P < 0.01; and *P < 0.05).

To investigate whether *Th*IPK1 and its downstream products serve as upstream signals for initiating the expression of lignocellulosic enzymes, a transcriptomic analysis was conducted comparing two *Thipk1* overexpression transformants with *D10* (Fig. S13), which were then further compared with the *D1* vs *D10* group. As representatively demonstrated by the comparison between *ov #1* vs *D10* group and *D1* vs *D10* group (Fig. 4A), for both overexpression transformants, there was a relatively high correlation among all genes (Pearson correlation of 0.56 or 0.47, respectively). The colony phenotype and sporulation of culture *D1*, along with the *Thipk1*-overexpressed transformants, and *D10*, along with the *Thipk1*-silenced transformants, exhibited a high level of consistency (Fig. S14). Interestingly, the transcriptional levels of lignocellulosic enzymes that were downregulated in *D10* compared to *D1* were nearly restored by *Thipk1* overexpression (Fig. 4A and B), resulting in a restored wood degradation ability (Fig. 4D through F; Fig. S15 and S16). Representative genes were chosen for qRT-PCR analysis in *Thipk1*-silenced transformants (Fig. 4C). All of these genes showed downregulation relative to *D1*, leading to a decreased wood degradation ability (Fig. 4D through F; Fig. S15 and S16). These results suggest that *Th*IPK1 transmits signals and regulates the transcription of most lignocellulosic enzyme genes to confer the strain with the ability to degrade wood chips.

The growth rates of the aforementioned transformants were also compared under different carbon sources. The results demonstrated that *Thipk1* overexpression effectively enhanced the culture’s capacity to utilize lignin, cellulose, and cellobiose, which was manifested in a faster cellobiose utilization rate (Fig. S17A and B), increased biomass (Fig. 4G; Fig. S17C), elevated intracellular ATP concentration (Fig. 4H), and reduced ROS concentration (Fig. 4I; Fig. S17D) in *Thipk1*-overexpressed transformants when compared to *D10*.

Transcriptome results further indicated that the expression patterns of genes related to apoptosis, autophagy, and cell cycle in *ov #1* and *ov #14* transformants resembled those observed in cultures *D1* rather than *D10* (Fig. S17E). In addition, qRT-PCR analysis of the *mTOR* and *AIF1* genes, as well as the Annexin V-PI staining results, suggested that an increase in *Thipk1* expression level reduced cell apoptosis when *D10* used cellulose and alkaline lignin as carbon sources (Fig. 4J; Fig. S18). Therefore, overexpression of *Thipk1* could restore colony morphology and physiological changes caused by strain degeneration, indicating potential downstream targets for *Th*IPK1. On the contrary, a decrease in *Thipk1* expression level led to increased apoptosis levels in the silenced transformants when grown on cellulose and alkaline lignin (Fig. 4J; Fig. S18), which explained the gradual death of mycelia observed when growing on wood (Fig. S16A through C).

### Four Zn_2_Cys_6_ transcription factors differentially regulate the transcription of lignocellulolytic enzyme genes in response to *Th*IPK1

**Fig 5:**
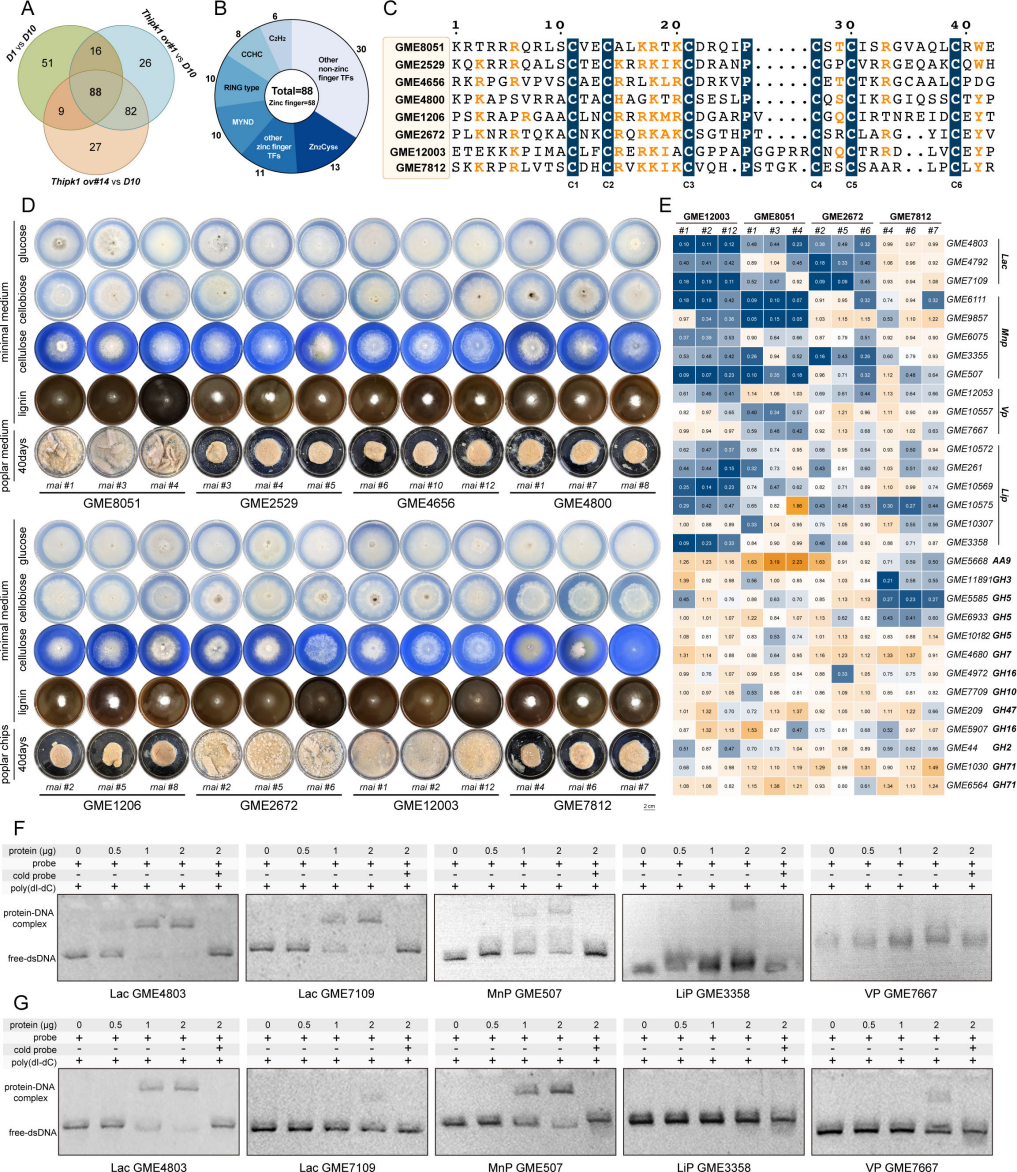
Characterization of the Zn_2_Cys_6_ transcription factor that regulates the expression of lignocellulolytic enzyme genes in response to *Th*IPK1. (**A**) The Venn diagram shows the number of differentially expressed genes that overlap between *D1* vs *D10*, *ov #1* vs *D10*, and *ov #14* vs *D10*. The false discovery rate is ≤0.05, and the fold change is >1.5. (**B**) Types of commonly differentially expressed transcription factors. The numbers indicate the number of transcription factors of this type. (**C**) Multiple sequence alignment of eight Zn_2_Cys_6_ protein DBDs. White letters indicate commonly conserved amino acids. (**D**) The growth phenotypes of different types of Zn_2_Cys_6_ transcription factor-silenced transformants in minimal medium with different carbon sources and liquid poplar medium. (**E**) Transcriptional analysis of lignocellulolytic enzyme genes in *GME12003*, *GME8051*, *GME2672*, and *GME7812*-silenced transformants. The yellow or blue box indicates up- or downregulated expression, respectively, and the numbers indicate the fold change relative to culture *D1*. (**F and G**) Interaction of GME12003 (**F**) and GME8051 (**G**) DBD with the promoter sequences (−500 bp upstream of ATG) of Lac *GME4803*, *GME7109*, MnP *GME507*, LiP *GME3358,* and VP *GME7667*.

A transcription factor (TF) enrichment analysis was performed to elucidate the mechanism underlying the function of *Th*IPK1 in regulating lignocellulosic enzymes. The expression profile of transcription factors showed moderate conservation based on the comparative transcriptomic analysis (Pearson correlation of 0.43) (Fig. S19A). In comparison to culture *D10*, a total of 88 transcription factors were found to be upregulated in both *D1* and *Thipk1*-overexpressed transformants (Fig. 5A; Table S5). Most of them belonged to the zinc finger family (Fig. 5B), with the Zn_2_Cys_6_-type being the most abundant (13). Among these, eight Zn_2_Cys_6_ transcription factors with the highest fold change at the transcription level were chosen for further investigation (Fig. S19B). Sequence alignment analysis revealed that although they shared low sequence identity among themselves (less than 26%), they all possessed a conserved Zn_2_Cys_6_-type DNA binding domain (Fig. 5C; Fig. S19C and D).

The RNA silencing of the eight TFs was carried out in culture *D1,* resulting in the successful construction of positive transformants (Fig. S20). These transformants were cultivated on different carbon sources and poplar substrates to investigate their regulatory role in lignocellulose degradation. The growth phenotype of the *GME12003*-silenced transformants closely resembled that of culture *D10* and *Thipk1*-silenced transformants, as their hyphae exhibited limited colonization ability on poplar. After 40 days of cultivation, the mycelia essentially underwent autolysis and died, indicating a reduced capacity for wood degradation compared to *D1* (Fig. 5D; Fig. S21). Furthermore, both the ligninolytic enzyme activities in the supernatant and their corresponding transcriptional levels were all significantly downregulated upon *GME12003* silencing in culture *D1*, leading to a substantial decrease in wood degradation ability (Fig. 5E; Fig. S22 and S23). However, CAZyme genes such as *GME11891* (GH3) and *GME5585* (GH5) remained largely unaffected, in agreement with the fact that they displayed similar colony diameters when grown on a single carbon source medium of cellulose (Fig. 5D).

Another two transcription factors, GME8051 and GME2672, showed regulation of Mnp and Lac expression but not Lip expression, resulting in weakened mycelial colonization on lignin along with decreased wood degradation abilities (Fig. 5D and E; Fig. S21 to S23). Conversely, *GME7812*-silenced transformants exhibited no change in growth rate when cultured on lignin or poplar but showed a significant decrease in a medium with cellulose as the sole carbon source (Fig. 5D). This was also supported by the decline in the transcription level and activity of cellulases, suggesting that GME7812 might be involved in cellulase gene transcription (Fig. 5E; Fig. S22 and S23).

The DBDs of GME12003 and GME8051 were heterologously expressed in *E. coli* and purified (Fig. S24). EMSAs revealed that the DBD of GME12003 exhibited binding affinity toward the promoter sequences (−500 to –0 bp) of *lacA*, *lacB*, *GME507* (MnP), and *GME3358* (Lip), while no binding was observed with respect to *GME7667* (Vp) (Fig. 5F). Similarly, EMSA experiments conducted with the DBD of GME8051 confirmed its direct transcriptional activity on Lac, MnP, and VP genes but not on LiP genes (Fig. 5G). These results provide conclusive evidence for the differential regulation exerted by Zn_2_Cys_6_ TFs on downstream lignocellulosic enzymes.

### Conservative evolution of IPK1 in white-rot fungi

**Fig 6:**
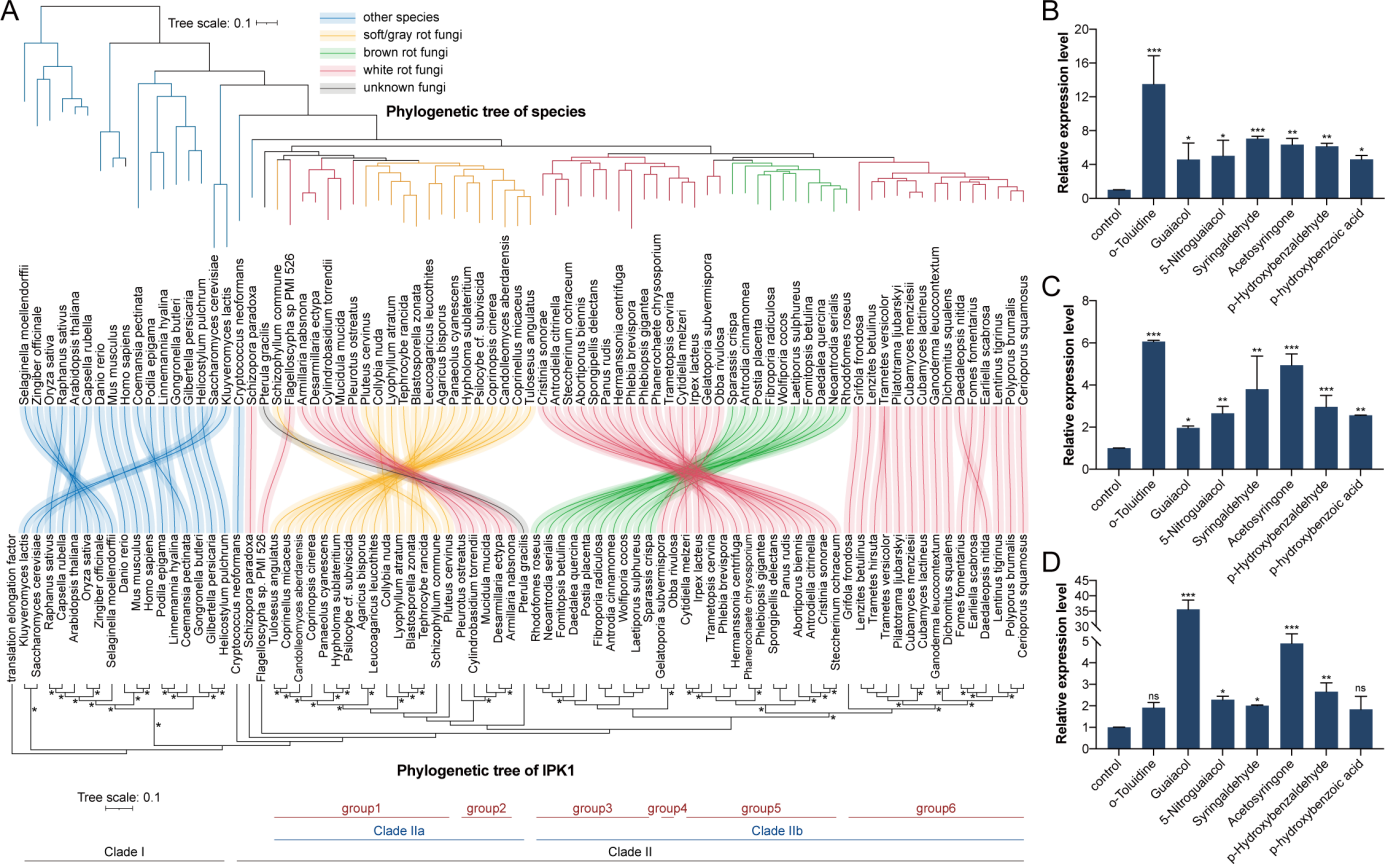
Conservation analysis of IPK1 in white rot fungi. (**A**) Phylogenetic tree analysis of the genome and IPK1 gene of some species. The species tree is drawn based on the genome analysis of the species. The red line indicates white-rot fungi, yellow indicates grass (soft)-rot fungi, green indicates brown-rot fungi, and blue indicates other species. Asterisk marks branches with >70% bootstrap support. (**B–D**) The transcription levels of the *ipk1* gene in *Trametes versicolor* (**B**), *Pleurotus ostreatus* (**C**), and *Schizophyllum commune* (**D**) after 48 h of treatment with lignin monomer compounds and derivatives.

The phylogeny relationships of a total of 81 IPK1 were analyzed, including 17 non-basidiomycete and 64 basidiomycete species (Table S2). A maximum likelihood phylogenomic tree was constructed to show the relationships (Fig. 6A). Similarly, the genomes of 80 species were also subjected to the same method for constructing their phylogenetic relationships. In comparison with other species, IPK1 from basidiomycetes was classified into Clade II, which further branches into two subclades. Specifically, Clade IIa consists of Agaricales, while Clade IIb comprises Polyporales. This evolutionary relationship was also observed in the genome phylogenetic tree.

Clade IIa could be further divided into two groups, with Group 1 consisting of wood (grass or gray) rot fungi that lack a complete lignin-degrading enzyme system and thrive on substrates such as straw and grass. White-rot fungi were clustered in Group 2, which included *Pleurotus ostreatus*. CladIIb was divided into Groups 3–6, encompassing brown-rot fungi and white-rot fungi, respectively. These findings demonstrated the correlation between the evolutionary relationship of *Th*IPK1 and the nutritional type of fungi, which could not be inferred from the genomic evolution alone. Notably, within Clade IIb, white-rot fungi in Groups 4, 5, and 6 showed a closer evolutionary relationship in IPK1 despite having distantly related genomes compared to brown-rot fungi (Group 3). Therefore, it is speculated that the function of IPK1 in *T. hirsuta* AH28-2 may be conserved in other white-rot fungi.

The white-rot fungi from different evolutionary clades, including *P. ostreatus*, *Trametes versicolor,* and gray-rot fungi *S. commune*, were selected for studying the response of IPK1 to different lignin monomers and derivatives. Similar to *T. hirsuta* AH28-2 (Fig. S25A and B), *T. versicolor Tvipk1* exhibited a rapid response of *IPK1* transcript level to *o*-toluidine exposure (Fig. 6B), resulting in a significant increase in laccase enzyme activity by 34.66-fold (Fig. S25C). In addition to G-type and S-type lignin monomers, *Tvipk1* also responded to H-type lignin monomers, leading to a notable enhancement in laccase enzyme activity (Fig. 6B; Fig. S25C). A similar phenomenon was observed in *P. ostreatus* (Fig. 6C; Fig. S24D). Apart from guaiacol and acetosyringone*, S. commune* displayed a weak response toward other lignin monomers and derivatives (Fig. 6D). Although there was a significant increase in laccase enzyme activity, the overall levels of enzyme activity remained relatively low (Fig. S25E).

### The pattern and function of DNA methylation in the regulation of wood degradation ability

**Fig 7:**
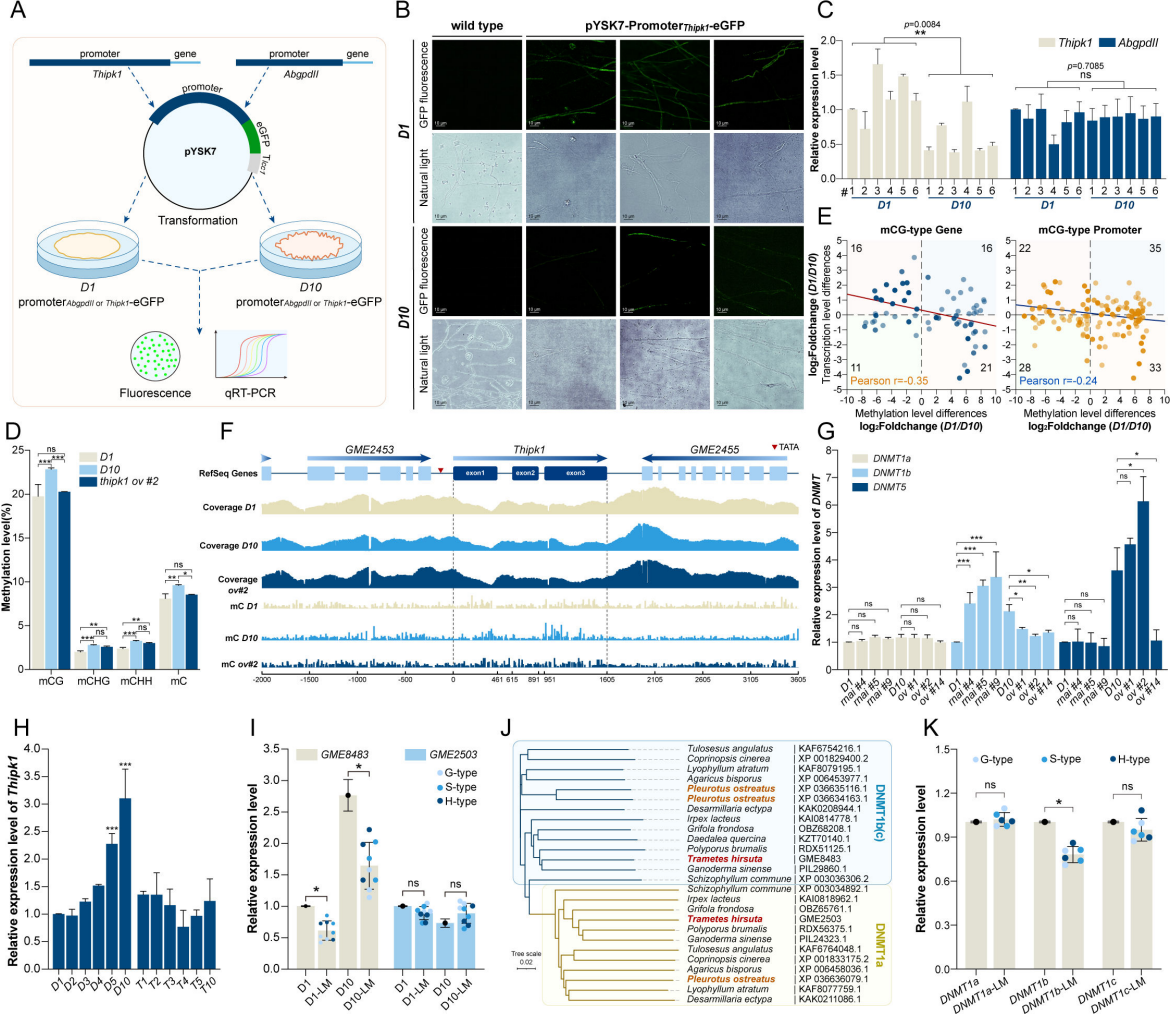
DNMT1b responds to lignin monomer compounds and derivatives. (**A**) Schematic diagram of the experiment to verify the transcription level of the *Thipk1* promoter in *T. hirsuta* AH28-2 *D1* and *D10* cultures. The −2,000 bp upstream of the *Thipk1* gene was identified as the promoter. The promoter-eGFP-terminator (terminator from the *Coprinopsis cinerea lcc1*) cassette was inserted into the pYSK7 vector and transformed into *D1* and *D10* cultures, respectively. The intensity of eGFP expression was detected by fluorescence intensity and transcription levels. The *AbgpdII* promoter from *Agaricus bisporus* was used as a control. (**B**) Fluorescence intensity of *D1* and *D10* cultures expressing eGFP using the *Thipk1* promoter. (**C**) The transcription levels of *eGFP* in the *D1* and *D10* cultures were driven by the *Thipk1* and *AbgpdII* promoters, respectively (**P < 0.01; ns, not significant). (**D**) The methylation rates of different methylation types across the whole genome for *T. hirsuta* AH28-2 *D1*, *D10*, and *ov #2* (***P < 0.001; **P < 0.01; *P < 0.05; and ns, not significant). (**E**) Correlation between the fold change in regional cytosine methylation rate (*X*-axis) and transcriptome expression difference (*Y*-axis) of *D1* vs *D10* mCG genomic differentially methylated region-related genes (blue) or promoters (orange) in the KEGG pathway. The numbers at the edges represent the number of genes located within each quadrant. (**F**) The coverage and methylation rates within 2,000 bp upstream and downstream of the *Thipk1* gene in *T. hirsuta* AH28-2 *D1*, *D10*, and *ov #2*. (**G**) The transcription levels of DNA methyltransferases *DNMT1a* and *DNMT1b* in *T. hirsuta* AH28-2 *D1*, *D10*, and *Thipk1*-silenced and -overexpressed transformants. (**H**) *DNMT1b* transcription levels in *T. hirsuta* AH28-2 *D1 –D10* and *T1 –T10* cultures (***P < 0.001). (**I**) The transcription levels of *DNMT1a* and *DNMT1b* in *T. hirsuta* AH28-2 *D1* and *D10* cultures after 48 h of treatment with different lignin monomer compounds (*P < 0.05; ns, not significant). (**J**) Phylogenetic tree analysis of DNMT1 in white-rot fungi. (**K**) The transcription levels of *DNMT1a*, *DNMT1b,* and *DNMT1c* in *P. ostreatus* after 48 h of treatment with different lignin monomer compounds.

Alterations in fungal genome methylation have significant effects on phenotype (46). Due to the GC- and CpG-rich sequences in the *Thipk1* promoter (Fig. S26A), to verify whether the silencing of *Thipk1* in *D1* during subculture is caused by methylation, we first constructed a plasmid to assess the strength of the *Thipk1* promoter in *D1* and *D10*. The sequence from −2,000 to 0 bp upstream of *Thipk1* was selected as the promoter, joined with *egfp* as the reporter gene. The plasmid was transformed into both *D1* and *D10* (Fig. 7A). Positive transformants were screened after verification by genomic PCR (Fig. S26B). The same procedure was performed using the *gpdII* promoter from *Agaricus bisporus* as a control (47). Randomly selected three positive clones from each group revealed that the fluorescence intensity of *D10-Thipk1-egfp* was significantly lower than that of *D1-Thipk1-egfp* (Fig. 7B), whereas no difference was observed when using *gpdII* as the reporter promoter (Fig. S26C). qRT-PCR results showed that the *egfp* transcription level in the *D1-Thipk1-egfp* group was significantly higher than in the D*10-Thipk1-egfp* group (Fig. 7C).

Whole-genome bisulfite sequencing results showed that compared to *D1*, *D10* exhibited increases in mCG, mCHG, and mCHH methylation rates by 3.08%, 0.79%, and 0.87%, respectively. This indicates mCG as the predominant differential methylation modification. Interestingly, the *Thipk1*-overexpressed transformant *ov #2* showed decreases in mCG, mCHG, and mCHH methylation rates by 2.558%, 0.21%, and 0.24%, respectively, compared to *D10* (Fig. 7D). The changes in methylation rates of entire transcriptional units, repeat, and CpG region also showed a similar trend (Fig. S27A). Moreover, the methylation fold change of all mCG/mCHG/mCHH-related genes or promoters within the genomic differentially methylated region in *D1* or *ov #1* relative to *D10* exhibited a high positive correlation, with only a few genes displaying different methylation patterns (Fig. S27B). KEGG enrichment analysis revealed that the correlated genes were predominantly involved in cellular metabolic pathways, including autophagy, cell cycle, lipid metabolism, etc., which was consistent with the aforementioned transcriptome results (Fig. S27C). However, the methylation fold changes of all mCG/mCHG/mCHH-related genes in *D1* relative to *D10* were negatively correlated with the transcriptional levels (Fig. 7E; Fig. S27D). Overexpression of *Thipk1* in *D10* restored the methylation and transcriptional levels of most genes (Fig. S27E). Thus, *Th*IPK1 modulates the methylation patterns of genes in *D10*, consequently influencing the expression of these genes. Interestingly, the methylation rate of the *Thipk1* gene sequence in *D10* increased compared to *D1*, while it remained consistent with that of *D1* after overexpression of *Thipk1* (Fig. 7F; Fig. S28A). Furthermore, the 5mC modification of transcription factor genes, including *GME12003*, *GME8051*, and *GME2672*, was analyzed. Compared to *D1*, their 5mC modification levels in *D10* significantly increased but were restored after *Thipk1* overexpression (Fig. S28B).

Further analysis revealed that *T. hirsuta* AH28-2 contains two DNMT1 (GME2503 and GME8483 are DNMT1a and DNMT1b, respectively), one DNMT2 (GME3676), and one DNMT5 (GME3814) (Fig. S28C). Apart from DNMT2, which is involved in tRNA-Asp methylation, DNMT1 and DNMT5 are extensively involved in CG methylation modifications of the fungal genome (48). Therefore, we speculated that the DNMT involved in methylation regulation was expressed at a high level in culture *D10* and was affected by *Th*IPK1. The transcriptional levels of *DNMT1a*, *DNMT1b*, and *DNMT5* in *D1*, *D10*, and other transformants were analyzed. The results showed that *DNMT1b* and *DNMT5* transcripts were higher in *D10* than *D1*, and the transcription of *DNMT1b* was affected by *Th*IPK1 (Fig. 7G).

### Regulation of white-rot fungus DNMT1b by lignin monomer compounds

Our results suggest that DNMT1b is the reason for *Thipk1* silencing during successive subcultures. qRT-PCR analysis showed that the expression level of DNMT1b continuously increased during the *D1* successive subculture, but there was no significant difference during the *T1* successive subculture (Fig. 7H). Furthermore, when *D10* was continuously cultured in media containing different concentrations of *o*-toluidine, which partially restored the phenotype of *D10* (Fig. 1F), the transcriptional level of *Thipk1* continuously increased while the *DNMT1b* decreased. There was no significant difference in the expression of DNMT1a (Fig. S29A). When cultures *D1* and *D10* were directly exposed to *o*-toluidine, *DNMT1b* was slightly downregulated, as shown by qRT-PCR (Fig. S29B). We further treated them with different types of lignin monomers, and the results showed that *DNMT1b* was downregulated in both *D1* and *D10* (Fig. 7I; Fig. S29C and D), indicating that DNMT1b was downregulated in response to lignin monomer in *T. hirsuta* AH28-2, which may be related to the activation of *Thipk1*.

To verify whether DNMT1 of other types of white-rot fungi also has a similar ability to respond to lignin monomers, we first conducted a phylogenetic analysis of DNMT1 of several white-rot fungi (Fig. 7J). Compared with the high consistency of DNMT1a (>30%), the consistency of DNMT1b in various species is relatively low and distributed on different branches. Different from *T. hirsuta*, the genome of *P. ostreatus* contains three types of DNMT1. The extra one is DNMT1c, which is highly consistent with DNMT1b. Interestingly, after *P. ostreatus* was treated with lignin monomers, the expression levels of DNMT1b and DNMT1c decreased to varying degrees, while DNMT1a showed no difference (Fig. 7K; Fig. S29E). The above results indicate that DNMT1 in white-rot fungi exhibits a conserved response to lignin monomers.

## DISCUSSION

The regulation of cellulose and lignin degradation processes by white-rot fungi has long been considered as two relatively independent processes. *Trametes* species prefer to degrade lignin and hemicellulose rather than cellulose when colonizing softwood, while they degrade lignin, cellulose, and hemicellulose simultaneously when colonizing hardwood (49), suggesting a potential consistency of *Trametes* in regulating the degradation of lignocellulosic components. We chose successive subculturing as the method to study the relationship between *Trametes’* regulation of cellulose, hemicellulose, and lignin degradation. The results showed that various ligninolytic enzymes, including Lac, MnP, Lip, and Vp, as well as various CAZymes involved in the degradation of cellulose and hemicellulose, were downregulated. The decrease in the strain’s ability to degrade cellulose and lignin seems to be accompanied by synchronous changes during the successive subculturing. We obtained a consistent conclusion through the phenotypes of strains growing on cellulose, alkaline lignin, and poplar at different generations. These results suggest that the regulation of cellulose and lignin degradation by *Trametes* may be coordinated through a common network. This was further confirmed by the fact that the strain could sustain lignocellulose degradation when successively subcultured on cPDA supplemented with *o*-toluidine.

White-rot fungi can produce a series of small aromatic compounds during the degradation of lignin (16). Research suggests that this type of compound plays an important role in the process of lignin degradation, including acting as electron transmitters and stimulating fungi to secrete ligninolytic enzymes (50). Although the specific mechanism has not been elucidated, it is believed that it only affects the degradation of lignin by the strain, with less effect on cellulose and hemicellulose degradation. We found that *o*-toluidine, guaiacol, etc., as a class of lignin monomer derivatives, can not only maintain the expression of ligninolytic enzymes but also inhibit the downregulated expression of cellulose and hemicellulose-degrading enzymes. This indicates a profound interconnection existing within the three regulatory networks. In addition, this class of aromatic compounds can maintain or reactivate the degradation of lignocellulose and the expression of lignocellulolytic enzyme by *T. hirsuta* AH28-2.

The InsP signaling pathway is widely present in animals, plants, fungi, and bacteria (30, 32, 51). It involves many pathways, including phosphate signal transduction, osmotic stress response, and regulation of glycolysis (34). We characterized the catalytic properties of IPK1, a key protein in the InsP pathway, in Basidiomycetes for the first time. The results indicate that *Th*IPK1 not only possesses conserved functions involved in responding to phosphate stress in strains but also exhibits a unique function in regulating fungal lignocellulolytic enzyme gene expression in response to lignin monomers, distinct from animals and plants. Silencing and overexpression of *Thipk1* significantly altered the expression levels of multiple enzyme systems, including lignin, cellulose, and hemicellulose degradation enzymes. This not only indicates that *Trametes* has a common regulatory network in regulating the degradation of lignin, cellulose, and hemicellulose but also shows that *Th*IPK1 plays an important role in this process. Furthermore, our results suggest that IPK1 is more highly conserved among white-rot fungi, and IPK1 homologs from the white-rot (or gray-rot) fungi *T. versicolor*, *P. ostreatus*, and *S. commune* respond to lignin monomer, consistent with *T. hirsuta* AH28-2. This conservation implies that IPK1 may play a conserved role in sensing lignin-derived signals and regulating lignocellulolytic enzyme expression in white-rot fungi. However, the functional role of IPK1 in basidiomycetes remains largely unknown. IPK1 in *C. neoformans* is an important gene that maintains virulence. Multiple fungal virulence-related genes of *ipk1Δ*, including laccase and urease, were significantly reduced. Further *kcs1* knockout also achieved the same effect, indicating that the key signaling molecule in regulating laccase in *C. neoformans* is PP-IP_5_ (28). PP-InsPs are signaling molecules generated by further phosphorylation of IP_6_ by IP6K and PPIP5K, including 1PP-InsP_5_, 5PP-InsP_5_, and 1,5PP_2_-IP_4_(29). Our results indicate that in *Thipk1*-silenced and overexpressed transformants, besides significant differences in InsP_6_ levels, there were synchronous changes in PP-InsPs. Therefore, these findings indicate that InsP_6_ and PP-InsPs may collectively participate in the regulation of lignocellulolytic enzymes. As an upstream synthetic gene, in addition to affecting the content of InsP_6_, IPK1 can also partially regulate the content of PP-InsPs (28). However, it is worth noting that after *Thipk1* silencing and overexpression, the magnitude of change in InsP_6_ levels is greater than that of PP-InsPs. Additionally, *Th*IPK1, as a key response protein, directly catalyzes the expression of InsP_6_, while IP6K and PPIP5K have not been found to respond to *o*-toluidine effectively. Therefore, we speculate that InsP_6_, as an important signaling molecule, regulates the differential expression of lignocellulolytic enzymes. However, the potential role of PP-InsPs should not be overlooked. Recent reports indicate that PP-InsPs, rather than InsP_6_, play a predominant role in maintaining fungal virulence and *Arabidopsis thaliana* response to phosphate signaling (29, 34, 52).

Overexpression of *Thipk1* in *D10* can effectively regulate the up- or downregulated lignocellulolytic enzyme genes caused by successive subculturing back to the *D1* level. We also obtained the same conclusion by RNA silencing of *Thipk1* in culture *D1*. Due to the diversity of downstream target genes, we speculate that *Th*IPK1 achieves differential regulation of various lignocellulolytic enzymes by controlling multiple transcription factors. Zn_2_Cys_6_, which specifically exists in fungi, is the most upregulated transcription factor. Many studies have also shown its involvement in regulating the expression of lignocellulolytic enzymes. Our results indicate that the silencing of *GME12003* effectively downregulates the expression of *lac*, *lip*, *mnp*, and *vp*. The DBD of GME12003 can directly bind to the promoter regions (from −500 to 0 bp) of *lacA*, *lacB*, *GME507* (Mnp), and *GME3358* (Lip). Similarly, we found that GME8051 and GME2672 also have similar functions, but they specifically participate in regulating laccase and manganese peroxidase. This indicates that multiple transcription factors under the *Th*IPK1 regulatory network cooperate to regulate ligninolytic enzymes differentially. Although it has been reported that Zn_2_Cys_6_-type transcription factors regulate fungal phosphate response (53), cellulose-degrading enzymes (54), and fungal pathogenicity (55), there are few reports on the regulation of ligninolytic enzymes. Our previous results demonstrated that TH8421 (GME8421) and TH4300 (GME4300) are directly involved in the expression of laccases *lacA* and *lacB* in response to *o*-toluidine by forming heterodimers (9). Here, we characterized that TH8421 may directly regulate the differential expression of laccases by responding to *Th*IPK1. Not only that, but we also found that silencing *GME7812* resulted in defects in the growth of the strain when using cellulose as a carbon source, while other types of carbon sources did not affect growth. Similar to our results, the Zn_2_Cys_6_-type Roc1 of *Schizophyllum commune* was identified as a key transcription factor that regulates cellulose degradation-related genes. The Roc1 knockout strain resulted in the inability to utilize cellulose and the downregulation of multiple cellulose-degrading enzymes, including LPMO (56). The Zn_2_Cys_6_ transcription factor PoxCxrA in *Penicillium oxalicum* also plays a similar function (57). As wood degrades, various types of lignocellulolytic enzymes synergistically collaborate at different stages (58). We proposed that *Th*IPK1 may regulate multiple specific transcription factors by regulating the concentration of InsP_6_ (PP-InsPs) to achieve controllable expression of different types of degradative enzymes.

The lower intracellular InsP_6_/PP-InsPs of *D10* resulted in the strain’s inability to effectively utilize lignin and cellulose, which led to lower intracellular ATP concentration and higher ROS levels when the strain grew on these carbon sources. It induced cell cycle arrest, apoptosis, and downregulated autophagy. PP-InsP_7_ is an important intracellular ATP concentration sensor in yeast. It increases intracellular ATP concentration by inhibiting glycolysis and promoting respiration (34). In addition, it also plays an important regulatory role in lipid synthesis and other pathways, which reflects that PP-InsP_7_ is at the center of cellular carbon metabolism (34). Not only that, PP-InsP_7_ synthesized by mammalian IP6K2 can bind and activate the protein kinase CK2 to trigger a signaling pathway promoting apoptosis (59). In HT-29 cells, InsP_6_ induces autophagy by inhibiting the mTOR pathway (60). In addition, InsP_6_ has also been reported to be involved in cell cycle regulation and apoptosis (61, 62). Thus, InsP_6_/PP-InsPs under the regulation of *Th*IPK1 may globally regulate the process of fungal degradation of lignocellulose to help fungi better adapt to the environment.

Reversible DNA methylation is an important way for fungi to respond to changes in the external environment, especially the strain degeneration caused by successive subculturing (63). The *Thipk1* promoter of culture *D10* has a higher m5C methylation level compared to *D1*. We speculate that this may be the reason for the gradual decrease in *Thipk1* expression level during passage. Comparatively, there is no significant difference in DNA methylation in the promoter regions of *ip6k* and *ppip5k*. Similarly, DNA methylation of the inositol-3-phosphate synthase (*Isyna1*) promoter, a key gene in the InsPs synthesis pathway, can effectively downregulate the expression of Isyna1 and the InsPs synthesis pathway (64). This implies that DNA methylation of the synthase gene may be a mechanism of regulation of the InsPs pathway. Unfortunately, we still do not have a clearer understanding. Interestingly, our results showed that after overexpression of *Thipk1* in *D10*, the total intracellular methylation/*Thipk1* promoter methylation decreased significantly, reaching levels similar to *D1*. This indicates that an increase in InsP_6_/PP-InsPs concentrations can reduce intracellular methylation levels and also illustrates the feedback regulation of *Thipk1* by InsP_6_/PP-InsPs in DNA methylation. This can be used to explain why *Thipk1* was not silenced during the successive subculturing of the strain in cPDA medium with *o*-toluidine. Furthermore, we found that the increase in InsP_6_/PP-InsPs levels reduced the DNA methylation levels of the Zn_2_Cys_6_ transcription factor and KEGG pathway genes, and this was correlated with their transcription levels. We speculate that this may be a way for IPK1 to regulate target genes. As an anticancer molecule, InsP_6_ can regulate the CpG DNA methylation of promoters for multiple genes, including *p16*, *MLH1*, and *COX-2*, and eliminate the carcinogenic effects of ethylnitrosourea on mouse lung cells (65). Furthermore, IP6K1 is an endogenous regulator of JMJD2C and H3K9 methylation, leading to defects in spermatogenesis and abnormal heterochromatin formation (66). However, the role of InsP_6_/PP-InsPs in DNA methylation remains unexplained. Therefore, we speculate that InsP_6_/PP-InsPs regulate the global DNA methylation modification network in *T. hirsuta* AH28-2, enabling precise and dynamic control of lignocellulolytic gene expression in response to environmental signals.

**Fig 8:**
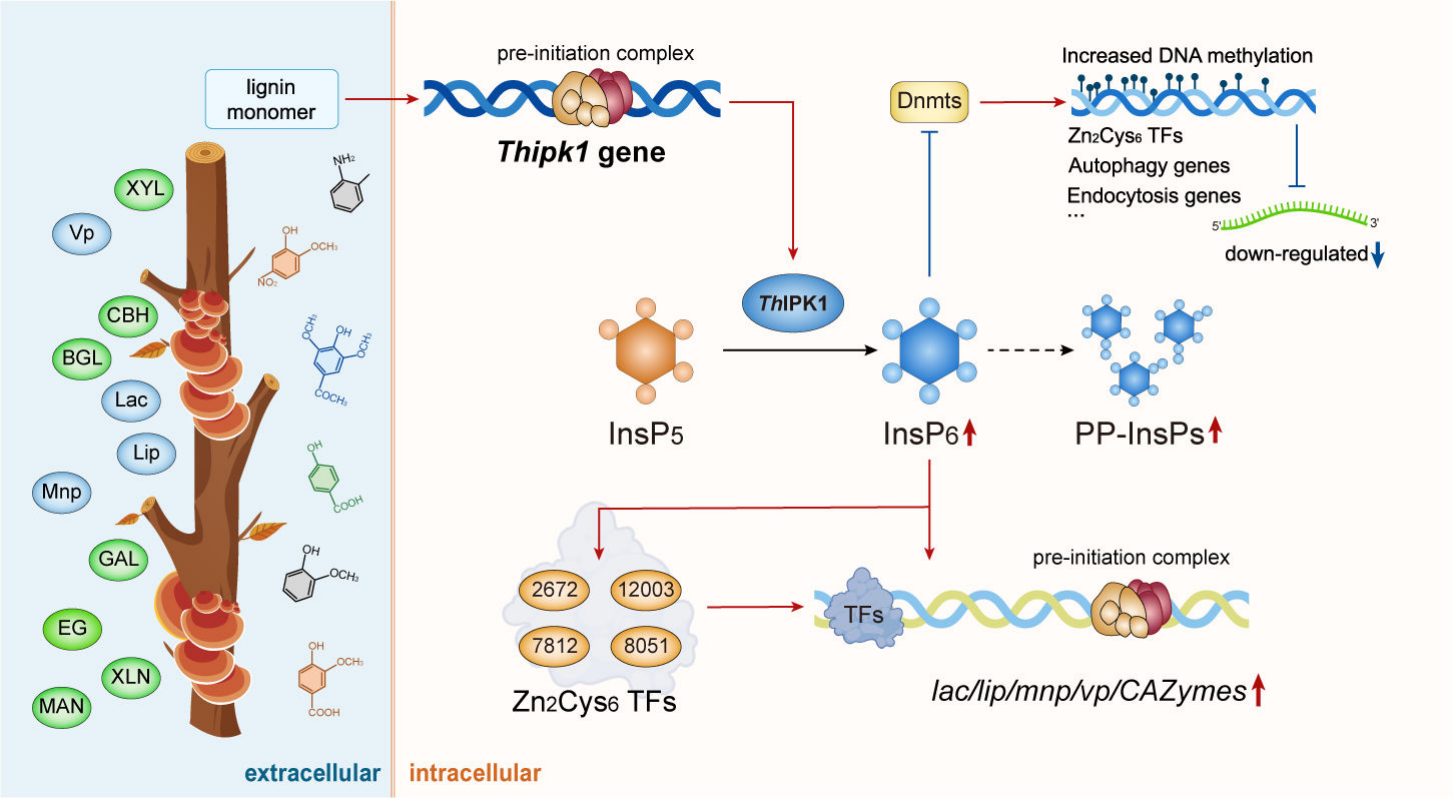
A schematic diagram of *Th*IPK1 regulating the lignocellulose degradation mechanism in *T. hirsuta* AH28-2 in response to lignin monomers. Red indicates activation/upregulation, and blue indicates inhibition/downregulation. Lignin monomers include G-type, S-type, and H-type, which have been studied.

In this study, we found that *Th*IPK1 directly and differentially responds to most types of lignin monomer compounds, further regulating a well-combined expression of lignocellulolytic enzymes in *T. hirsuta* AH28-2. Through successive subculturing, we have demonstrated that *T. hirsuta* possesses a unified regulatory network for the degradation of lignin, cellulose, and hemicellulose. The response of IPK1 is relatively conservative among white-rot fungi. These results have enriched our understanding of how white-rot fungi regulate the degradation process of lignocellulose. Furthermore, we found that *Th*IPK1 upregulates intracellular levels of InsP_6_/PP-InsPs in *T. hirsuta* AH28-2, which in turn differentially regulates the expression of lignocellulolytic enzymes, facilitating fungal growth on wood as a carbon source. Genes involved in autophagy and cell cycle maintenance were also upregulated, contributing to improved fungal fitness. Notably, a large number of Zn_2_Cys_6_ transcription factors were induced, among which GME12003, GME 8051, and GME2672 were identified as key regulators responding to InsP_6_/PP-InsPs-mediated differential modulation of lignocellulolytic enzymes. In addition, InsP_6_/PP-InsPs may exert global regulatory effects through DNA methylation, influencing the expression of *Thipk1*, transcription factors, and other functional genes (Fig. 8). These findings underscore the dynamic and multilayered regulatory strategies employed by the fungus in response to environmental signals.

## Data Availability

Sequencing data will be available at Sequence Read Archive (PRJNA1213972, PRJNA1214041, and PRJNA1214043). All data needed to evaluate the conclusions in the paper are presented in the paper and/or the supplemental material. The address of the *Thipk1* phylogenetic tree is https://itol.embl.de/tree/10423324120273621744686274. The address of the species phylogenetic tree is https://itol.embl.de/tree/10423324118456791725004596.

## SUPPLEMENTAL MATERIAL

Supplemental material mbio.01243-25-s0001.pdf Fig. S1 to S29; Tables S1 to S5.

ASM does not own the copyrights to Supplemental Material that may be linked to, or accessed through, an article. The authors have granted ASM a non-exclusive, world-wide license to publish the Supplemental Material files. Please contact the corresponding author directly for reuse.
