# Supplementary material for: ThIPK1 regulates lignocellulolytic enzyme expression during wood degradation in white-rot fungi
Source: mBio. 2025 Aug 18;16(9):e01243-25. doi: 10.1128/mbio.01243-25 (PMC12421882; doi:10.1128/mbio.01243-25)
Supplement: Supplemental material — Fig. S1 to S29; Tables S1 to S5. [file mbio.01243-25-s0001.pdf]

Supplementary material

*Th*IPK1 regulates lignocellulolytic enzyme expression during wood degradation in white-rot fungi

Xinlei Zhang[1,2,3], Rong Zhu[1,2,3], Diao Ying[1,2,3], Chengkai Wang[1,2,3], Shenglong Liu[1,2,3], Ursula Kües[4], Rong Jia[1,2,3], Yazhong Xiao[1,2,3], Zemin Fang[1,2,3*], Juanjuan Liu[1,2,3*]

[1] School of Life Sciences, Anhui University, 230601 Hefei, Anhui, China

[2] Anhui Key Laboratory of Biocatalysis and Modern Biomanufacturing, 230601 Hefei, Anhui, China

[3] Anhui Provincial Engineering Technology Research Center of Microorganisms and Biocatalysis, 230601 Hefei, Anhui, China

[4] Büsgen-Institut, Molecular Wood Biotechnology and Technical Mycology and Goettingen Center for Molecular Biosciences (GZMB), University of Goettingen, Büsgenweg 2, 37077, Goettingen, Germany

*Corresponding authors

Supplementary Figures

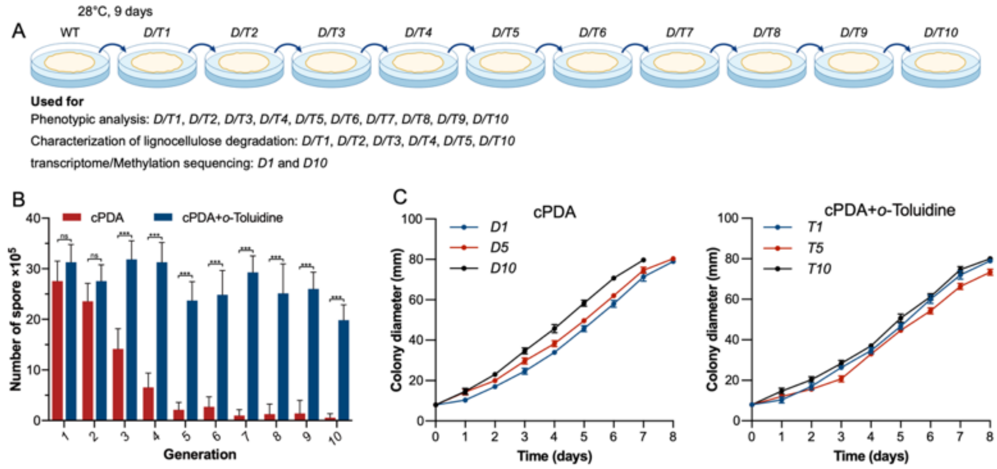

Fig. S1 Characterization of different *T. hirsuta* AH28-2 cultures on cPDA plates. **(A)** Schematic diagram of successive subculture of *T. hirsuta* AH28-2. **(B)** The number of oidia of *D1~D10* and *T1~T10* cultures after 9 d of growth (90 mm petri dishes). **(C)** The growth rate of *D1~D10* and *T1~T10* cultures.

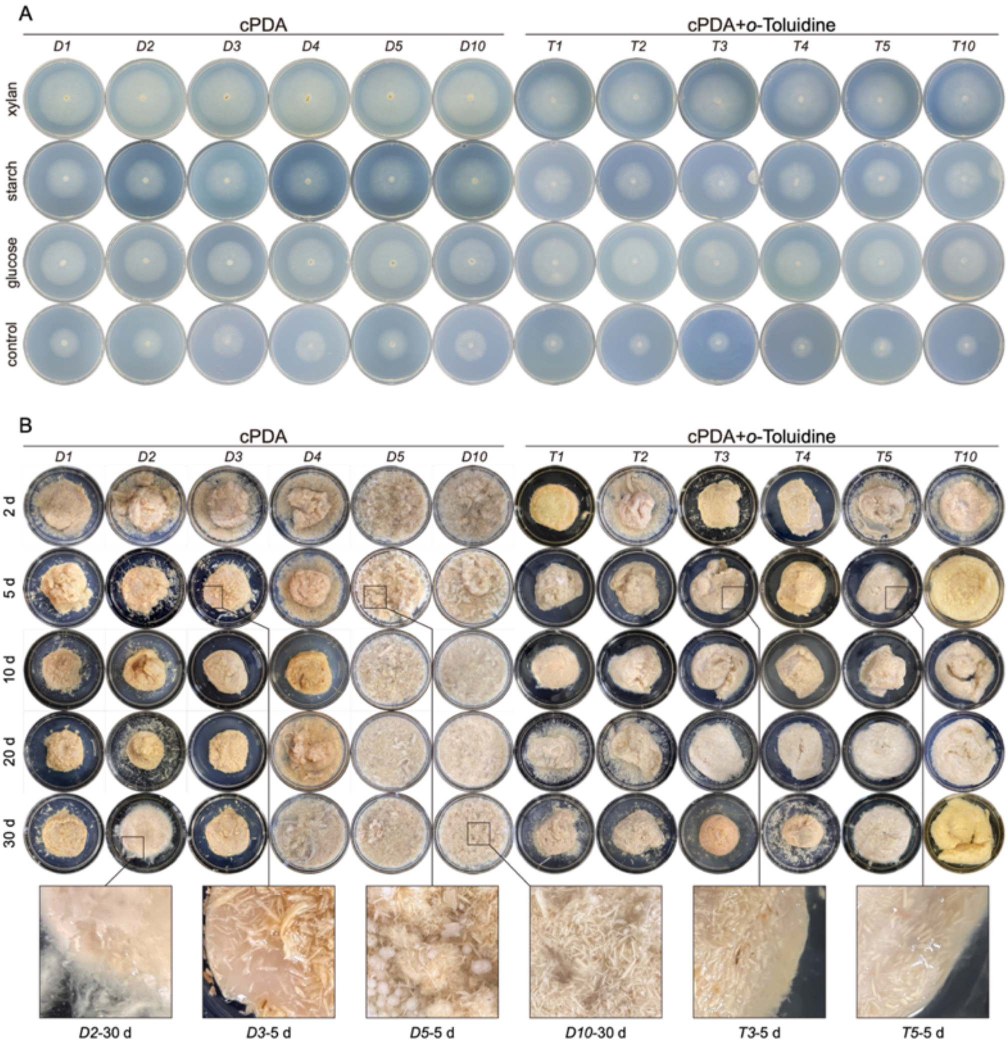

Fig. S2 Growth phenotypes of *T. hirsuta* AH28-2 *D1~D10* and *T1~T10* cultures on various carbon sources. (A) Cultures were grown in a minimal medium with xylan, starch, or glucose as a carbon source for 6 d. All cultures were grown from spot-inoculated oidia suspensions at 28°C. Controls without any carbon source were grown for 3 d. **(B)** Cultures were grown in a liquid medium containing poplar wood chips. Enlarged images can show the morphological differences between different cultures.

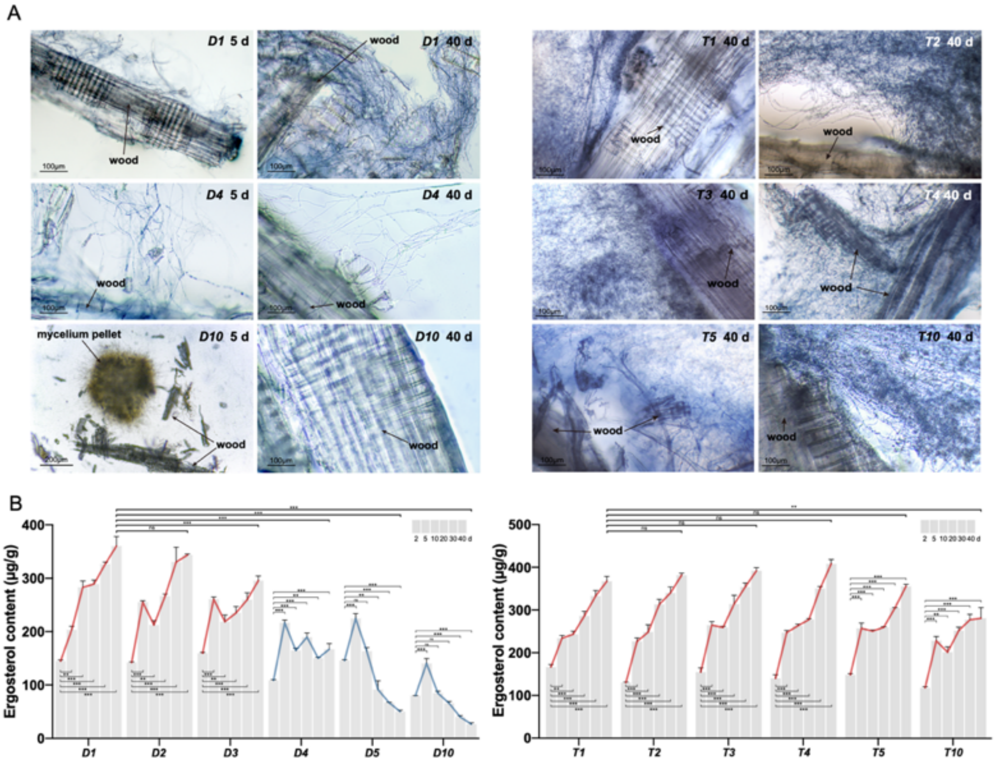

Fig. S3 Phenotypes (A) and ergosterol contents (B) of different *T. hirsuta* AH28-2 cultures growing on poplar. (A) Optical microscope images. The strains appeared blue following staining. Scale bar, 100 μm. **(B)** Cultures were grown for 40 d. The ergosterol content was positively correlated with the fungal mycelial biomass.

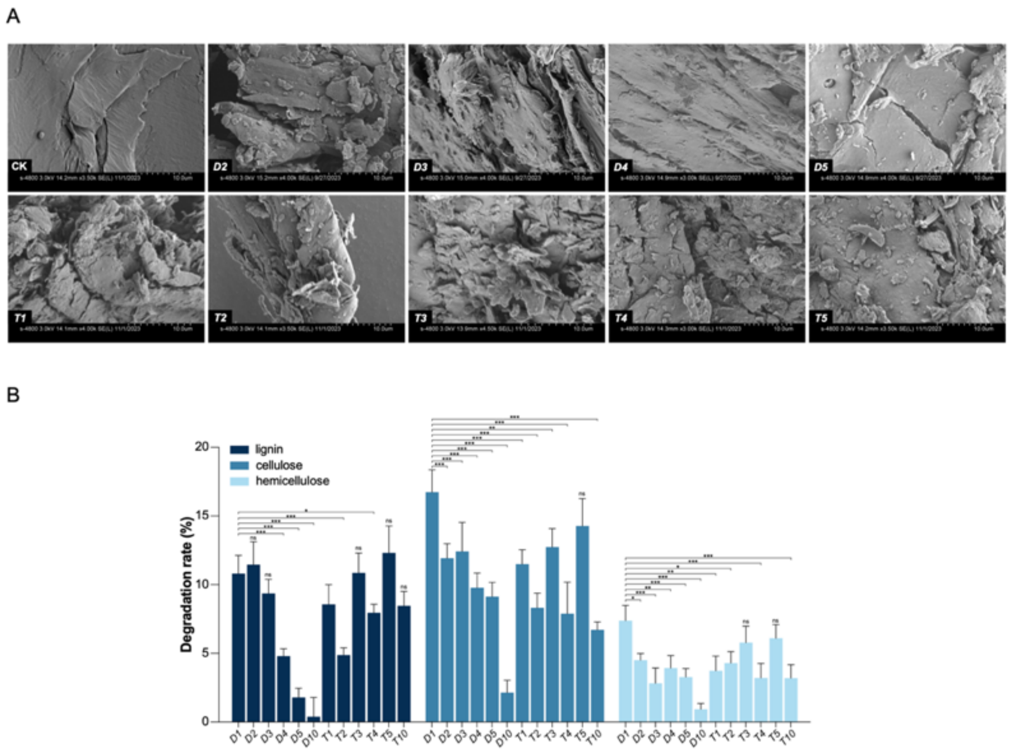

Fig. S4 Characterization of lignocellulose degradation by different *T. hirsuta* AH28-2 cultures. **(A)** SEM images of poplar wood after 40 d of treatment. Scale bar, 10 μm. **(B)** The degradation rate of lignocellulose in poplar wood after 40 d of treatment. The “degradation rate” represented the extent of reduction in lignin, cellulose, or hemicellulose after cultivation relative to their respective initial amounts in the non-inoculated poplar medium.

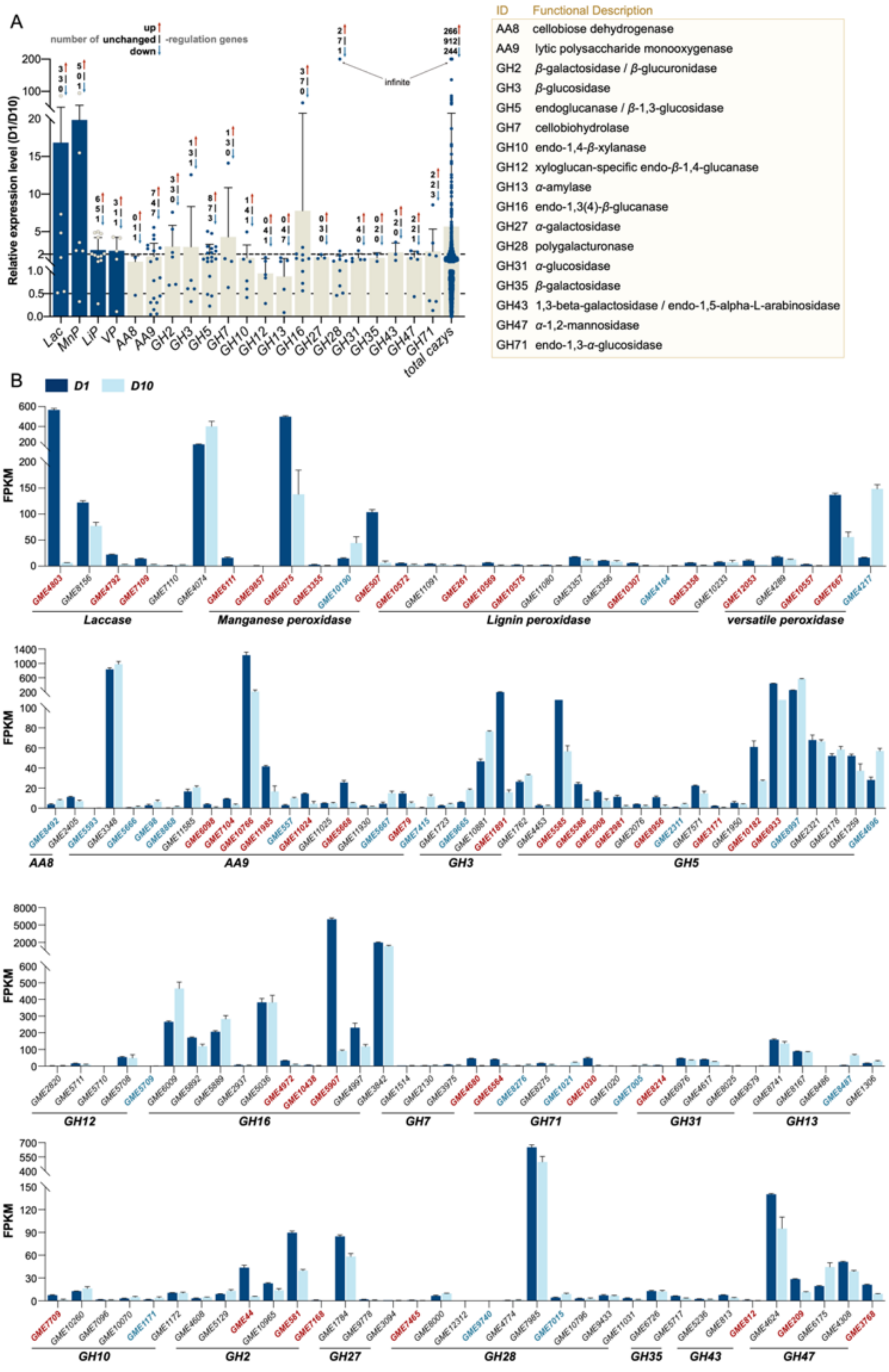

Fig. S5 Transcriptional analysis of lignocellulosic enzyme genes in *T. hirsuta* AH28-2 *D1* and *D10* cultures. (A) Transcriptional fold changes and functional annotation of putative lignocellulolytic enzyme genes between cultures *D1* and *D10*. **(B)** FPKM of putative lignocellulolytic enzyme genes between cultures *D1* and *D10*.

Red and blue represent the up-regulation and down-regulation of expression in *D1*, respectively.

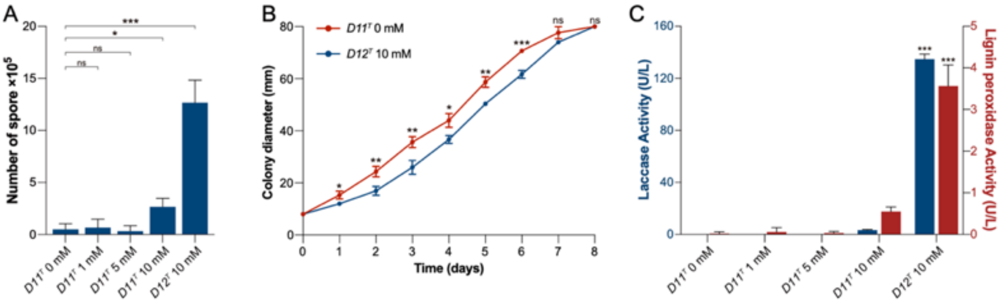

Fig. S6 Restoration of the phenotypes of culture *D10* following treatment with *o*-toluidine. **(A)** The number of oidia after 9 d of growth on cPDA plates (90 mm). **(B)** The growth rate of $D11^T$ and $D12^T$ cultures. **(C)** Lac and LiP activities in XH medium with cellobiose as carbon source.

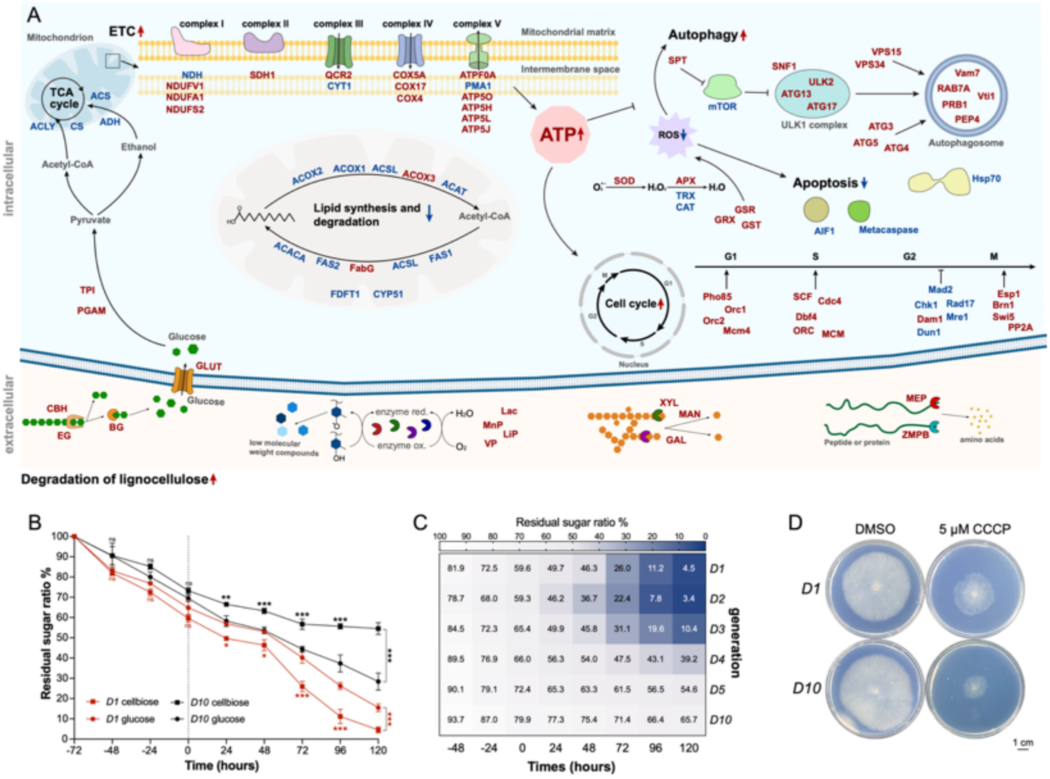

Fig. S7 Differences in carbon source utilization and physiological metabolism between *T. hirsuta* AH28-2 *D1* and *D10* cultures. (A) Model diagram of transcriptome analysis. Red-labeled genes, compounds, and pathways indicate up-regulation in the *D1* culture, while green-labeled ones indicate down-regulation. **CBH**, cellobiohydrolase; **EG**, endo-glucanase; **BG**, *β*-glucosidase; **Lac**, laccase; **MnP**, manganese peroxidase; **LiP**, lignin peroxidase; **VP**, versatile peroxidase; **XYL**, *β*-xylosidase; **GAL**, galactosidase; **MAN**, *β*-mannanase; **MEP**, metalloprotease; **ZMPB**, zinc metalloprotease; **GLUT**, glucose transporter; **PGAM**, phosphoglycerate mutase; **TPI**, triosephosphate isomerase; **CS**, citrate synthase; **ADH**, alcohol dehydrogenase; **ACS**, acetyl-CoA synthetase; **ACLY**, ATP citrate lyase; **ETC**, electron transport chain; **NDH**, NADH dehydrogenase; **NDUFV1**, **NDUFA1**, and **NDUFS2**: NADH dehydrogenase (ubiquinone) flavoprotein subunits; **SDH1**, succinate dehydrogenase; **QCR2**, ubiquinol-cytochrome c reductase subunit 2; **CYT1**, ubiquinol-cytochrome c oxidoreductase; **COX5A**, **COX17**, and **COX4**, cytochrome c oxidase subunit; **PMA1**, plasma membrane ATPase 1; **ATPF0A**, **ATP5O**, **ATP5H**, **ATP5L**, and **ATP5J**, ATPase subunit; **SPT**, serine palmitoyltransferase; **mTOR**, mammalian target of rapamycin; **SNF1**, carbon catabolite-derepressing protein kinase; **ULK2**, serine/threonine-protein kinase; **ATG13**, **ATG17**, **ATG3**, **ATG5**, and **ATG4**,

autophagy-related protein; **VPS34**, phosphatidylinositol 3-kinase; **VPS15**, Phosphoinositide 3-kinase regulatory subunit 4; **Vam7**, vacuolar morphogenesis 7; **PRB1**, proline rich protein BstNI subfamily 1; **PEP4**, saccharopepsin; **Vti1**, vesicle transport through interaction with t-SNAREs 1; **SOD**, superoxide dismutase; **APX**, ascorbate peroxidase; **TRX**, thioredoxin; **CAT**, catalase; **GST**, glutathione S-transferase; **GSR**, glutathione reductase; **GRX**, glutaredoxin; **AIF1**, apoptosis-inducing factor; **Hsp70**, heat shock protein 70; **Orc1** and **Orc2**, origin recognition complex subunit; **Mcm4**, minichromosome maintenance complex component 4; The remaining proteins are associated with the cell cycle. **(B)** Sugar utilization efficiency of cultures *D1* and *D10* grown in minimal liquid medium with glucose or cellobiose as carbon source. The 0 h time point refers to the strain being homogenized, inoculated into the culture medium, and incubated for 72 h. **(C)** Sugar consumption rates of cultures *D1~D10* grown in a minimal liquid medium with cellobiose as the carbon source. **(D)** Phenotypes of cultures *D1* and *D10* grown in a minimal medium with glucose as the carbon source. CCCP at a final concentration of 5 μM was added, with DMSO as the control.

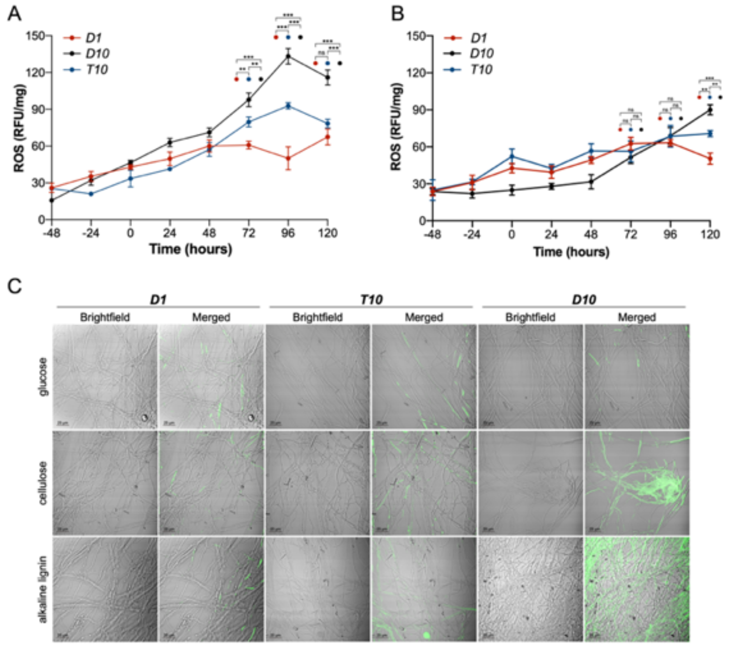

Fig. S8 Intracellular ROS in *T. hirsuta* AH28-2 *D1*, *D10*, and *T10* cultures under different carbon sources. (A and **B)** ROS concentrations of cultures grown in a liquid medium with cellobiose (A) or glucose (B) as the carbon source. **(C)** ROS levels indicated by staining with DCFH-DA and imaged under a confocal laser scanning microscope. Scale bar, 10 μm.

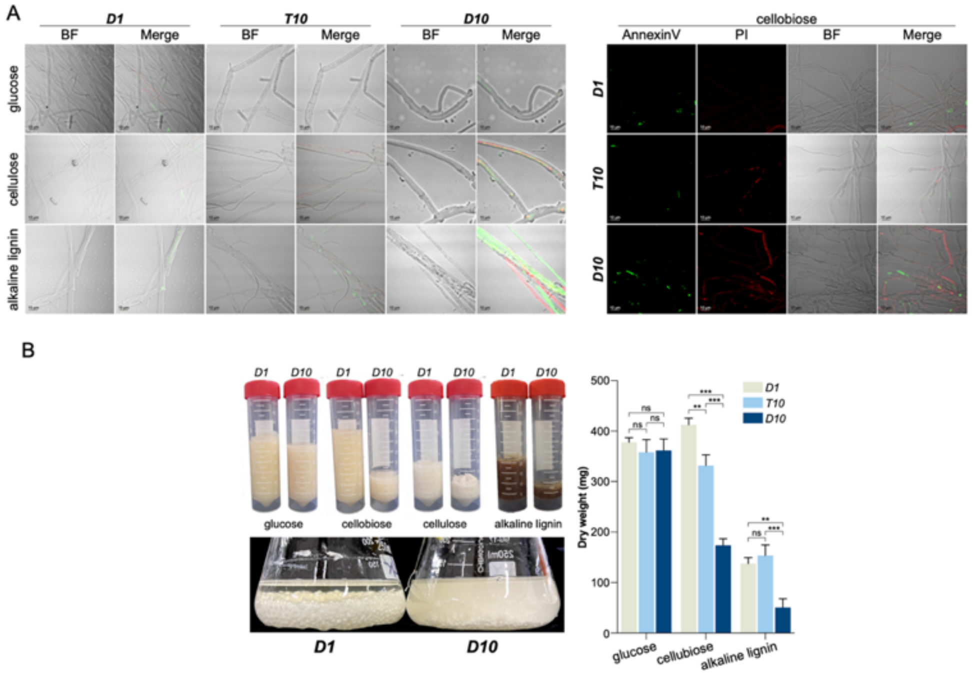

Fig. S9 Apoptosis levels and biomasses of *T. hirsuta* AH28-2 *D1*, *D10*, and *T10* cultures under different carbon sources. (A) Mycelia of different cultures grown on a solid medium containing various carbon sources were co-stained with Annexin V–PI and imaged under a confocal laser scanning microscope. Scale bar, 10 µm. **(B)** Biomasses of different cultures after 7 d of growth in a liquid medium with various carbon sources. The mycelium of *T. hirsuta* AH28-2 can adsorb water-insoluble cellulose particles, which precludes the use of dry weight as a reliable method for comparing biomass differences. In the cellulose medium, the *D1* culture exhibited a stronger ability to adsorb cellulose particles and showed higher biomass compared to *D10*.

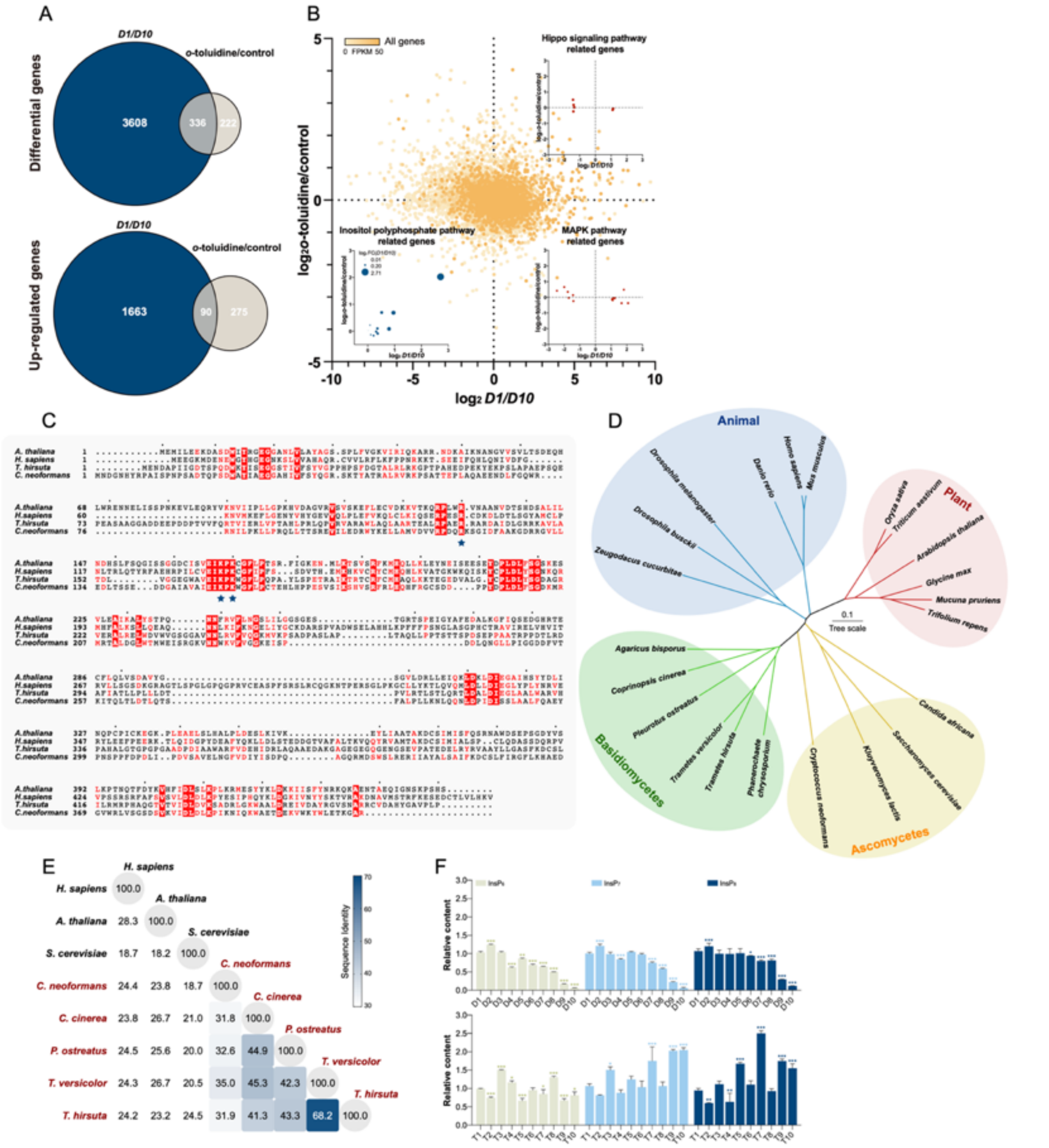

Fig. S10 Identification and analysis of key genes in the transcriptome. (A) The Venn diagram shows the number of differentially expressed genes that overlap between the *D1* vs. *D10* group and the *o*-toluidine vs. control group. The false discovery rate (FDR) is ≤ 0.05. The absolute $\log_2$ fold change (abs($\log_2$FC)) is ≥ 1.0, with upregulated genes having a $\log_2$FC ≥ 1. **(B)** The scatter plots show the correlation of differential expression folds of all genes in the *D1* vs. *D10* group (X-axis) and *o*-toluidine vs. control group (Y-axis). The correlation analysis between the two groups of genes in the Hippo signaling pathway, Inositol polyphosphate pathway, and MAPK pathway is also shown. **(C)** The multiple sequence alignment of the potential IPK1 from *T. hirsuta* AH28-2 and the characterized IPK1 from *Arabidopsis thaliana*, *Homo sapiens*, and *Cryptococcus neoformans*. The asterisks mark the core catalytic motifs of IPK1. **(D)** Phylogenetic tree analysis of IPK1 from different

species. The blue box represents animals, red represents plants, yellow represents ascomycetes, and green represents basidiomycetes. **(E)** The IPK1 from *T. hirsuta* AH28-2 shares over 30% sequence identity with basidiomycota (red) but shows a lower identity to sequences from other species (black). **(F)** Grayscale analysis of intracellular $InsP_6$ and PP-InsPs contents in *T. hirsuta* AH28-2 *D1~D10* and *T1~T10* cultures.

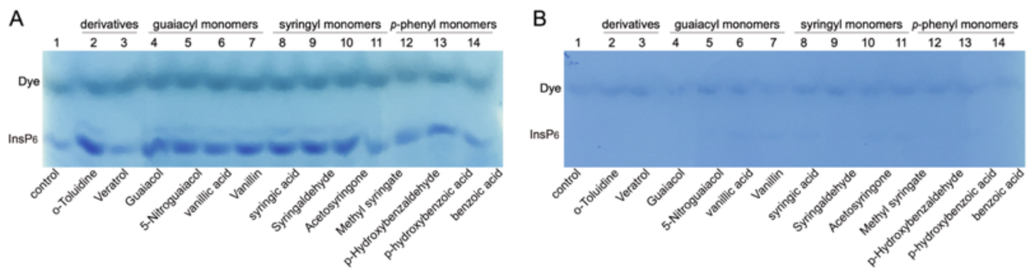

Fig. S11 Effects of lignin monomer compounds and derivatives on the contents of $InsP_6$ and PP-InsPs in *T. hirsuta* AH28-2. Characterization of intracellular $InsP_6$ and PP-InsPs contents in *D1* (A) and *D10* (B) cultures after 48 h of treatment with different types of lignin monomer compounds and derivatives. Intracellular $InsP_6$ and PP-InsPs were adsorbed on TiO_2 and then separated and characterized using PAGE.

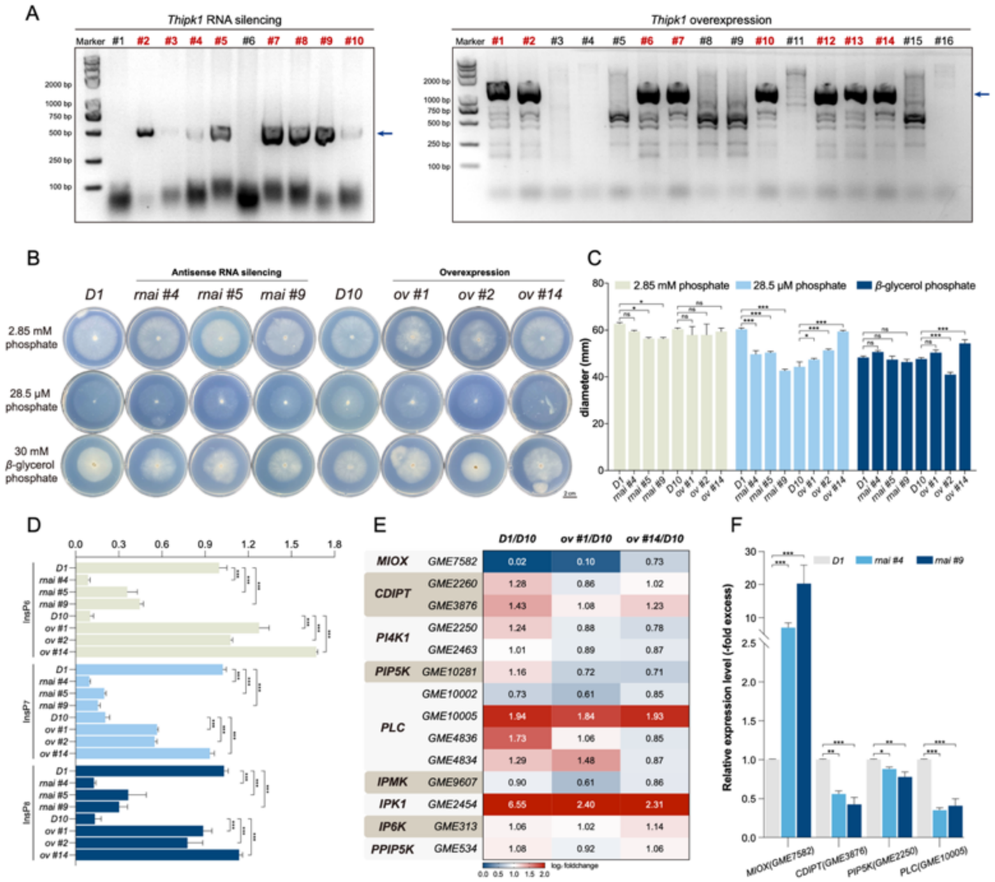

Fig. S12 Construction and verification of *T. hirsuta* AH28-2 *Thipk1* silencing and overexpression strains. (A) The genomic PCR verification of *Thipk1*-silenced and overexpressed transformants. No specific bands were found in the genomic PCR of the wild-type strain. The blue arrow shows the specific target band, which was verified by sequencing. **(B)** Phenotypes of *T. hirsuta* AH28-2 *D1*, *D10*, and *Thipk1*-silenced and -overexpressed strains in minimal solid medium containing 2.85 mM phosphate, 28.5 μM phosphate, and 30 mM *β*-glycerol phosphate, respectively. All cultures were grown from spot-inoculated oidia suspensions at 28°C for 6 d. **(C)** Diameter statistics in (B). **(D)** Grayscale analysis of intracellular $InsP_6$ and PP-InsPs concentrations in *T. hirsuta* AH28-2 *D1*, *D10*, and *Thipk1*-silenced and -overexpressed cultures. **(E)** Transcriptomic analysis revealed the effect of *Thipk1* overexpression on the expression levels of genes involved in the inositol polyphosphate pathway. (F) qRT-PCR analysis revealed the effect of *Thipk1* silencing on the expression levels of some genes in the inositol polyphosphate pathway.

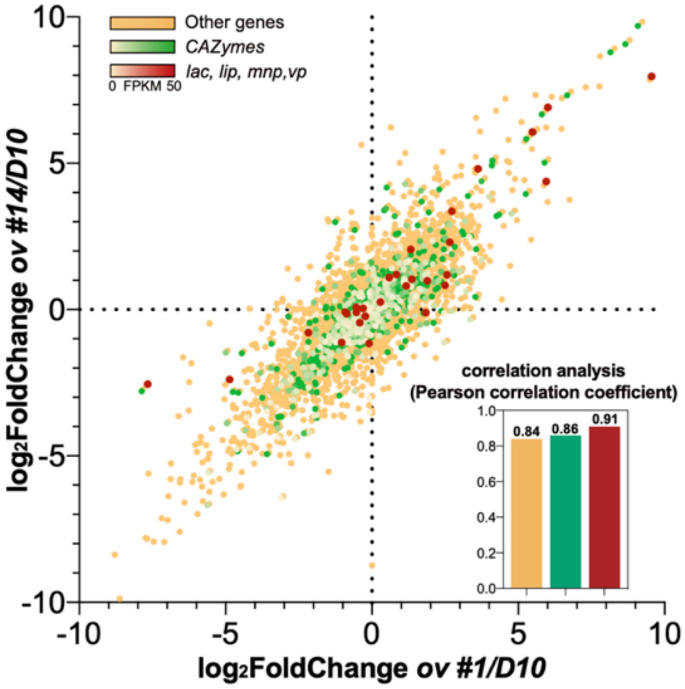

Fig. S13 Correlation analysis of transcriptomes of *Thipk1*-overexpressed transformants *ov #1* and *ov #14*. The scatter plots show the correlation of the expression levels of all genes in the *ov#1* vs. *D10* group (X-axis) and *ov#14* vs. *D10* (Y-axis). The Pearson correlation coefficients of all genes (yellow), CAZymes genes (green), and lignocellulosic enzyme genes (red) between the two different overexpression transformants were 0.84, 0.86, and 0.91, respectively.

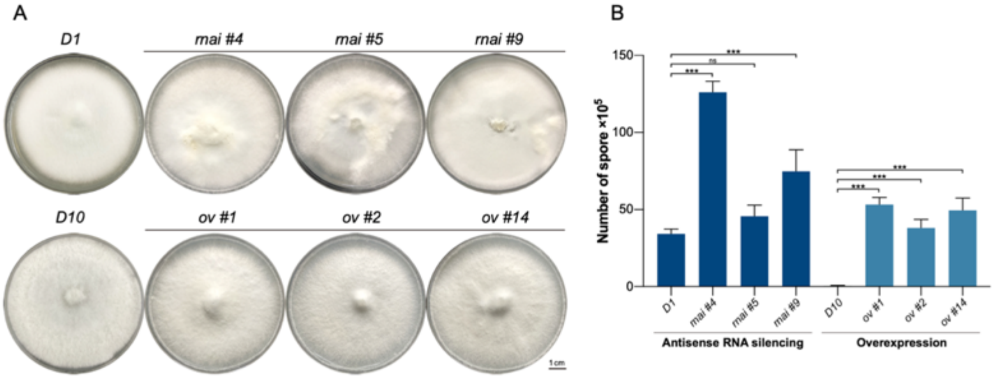

Fig. S14 Phenotypic analysis of *Thipk1*-silenced and -overexpressed transformants on cPDA plates. Colony morphology (A) and the number of oidia (B) of *T. hirsuta* AH28-2 *D1*, *D10*, and *Thipk1*-silenced and -overexpressed transformants after 9 d of growth on cPDA plates (90 mm).

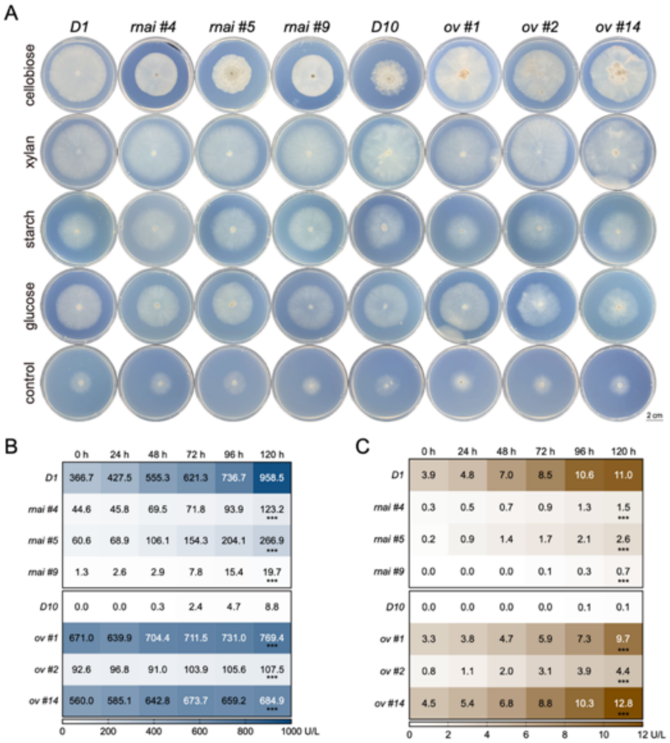

Fig. S15 Phenotypic analysis of *Thipk1*-silenced and -overexpressed transformants on plates with different carbon sources. (A) Growth phenotypes of *T. hirsuta* AH28-2 *D1*, *D10*, and *Thipk1*-silenced and -overexpressed transformants in a minimal medium with cellulose, cellobiose, lignin, xylan, starch, or glucose as a carbon source for 6 d. All cultures were grown from spot-inoculated oidia suspensions at 28°C. Controls without any carbon source were grown for 3 d. **(B and C)** Lac (B) and LiP (C) activities of different *T. hirsuta* AH28-2 cultures in XH medium with cellobiose as a carbon source (U/L).

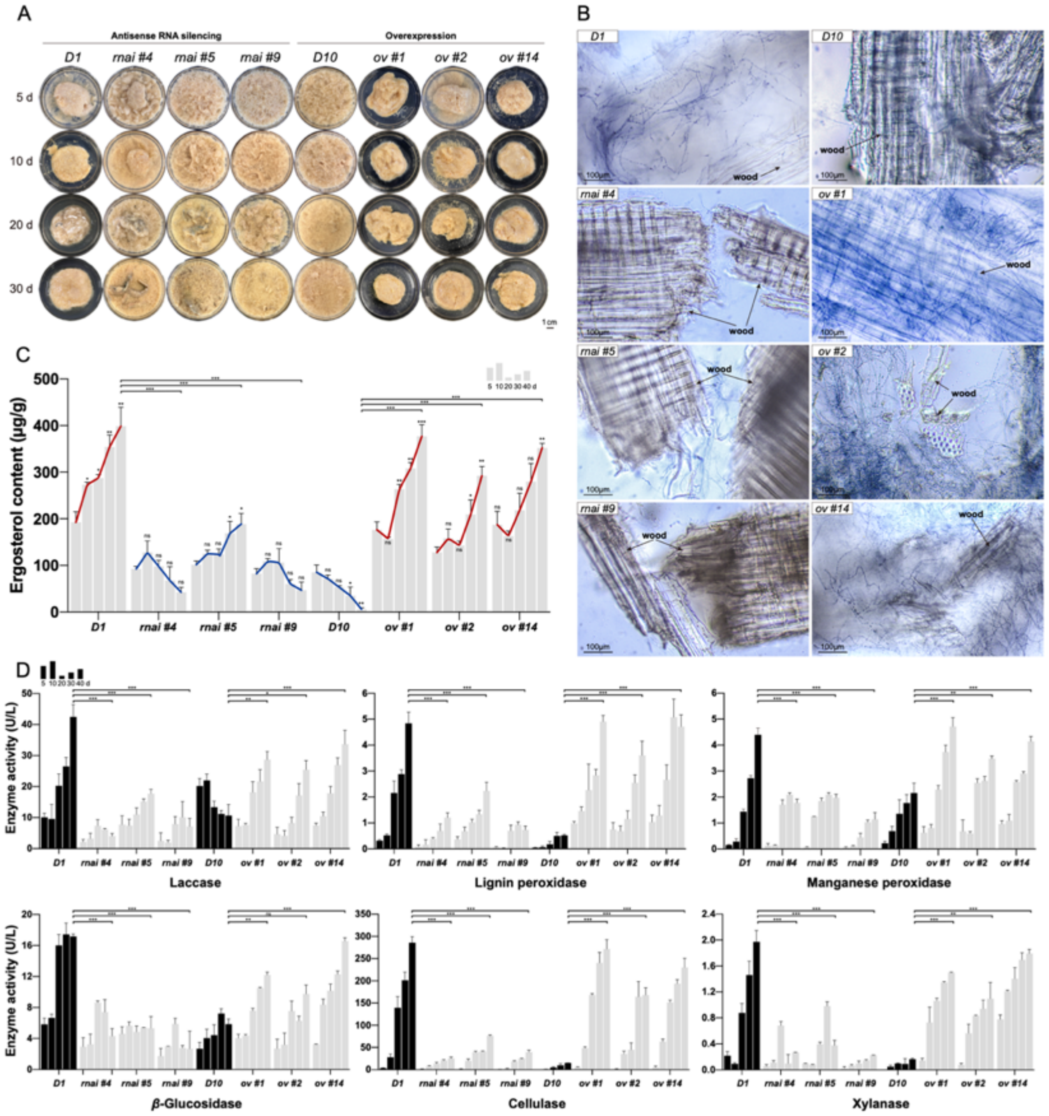

Fig. S16 Phenotypic analysis of *Thipk1*-silenced and -overexpressed transformants growing on poplar. **(A)** Growth phenotypes of *T. hirsuta* AH28-2 *D1*, *D10*, and *Thipk1*-silenced and -overexpressed transformants cultured in liquid medium containing poplar at different time points. **(B)** Optical microscope image analysis. The strains appear blue following staining. Scale bar, 100 μm. **(C)** The ergosterol content of different cultures grown on poplar wood at different time points. The ergosterol content is positively correlated with the fungal mycelial biomass. **(D)** Characterization of the activities of various lignocellulolytic enzymes after cultivation for 40 d.

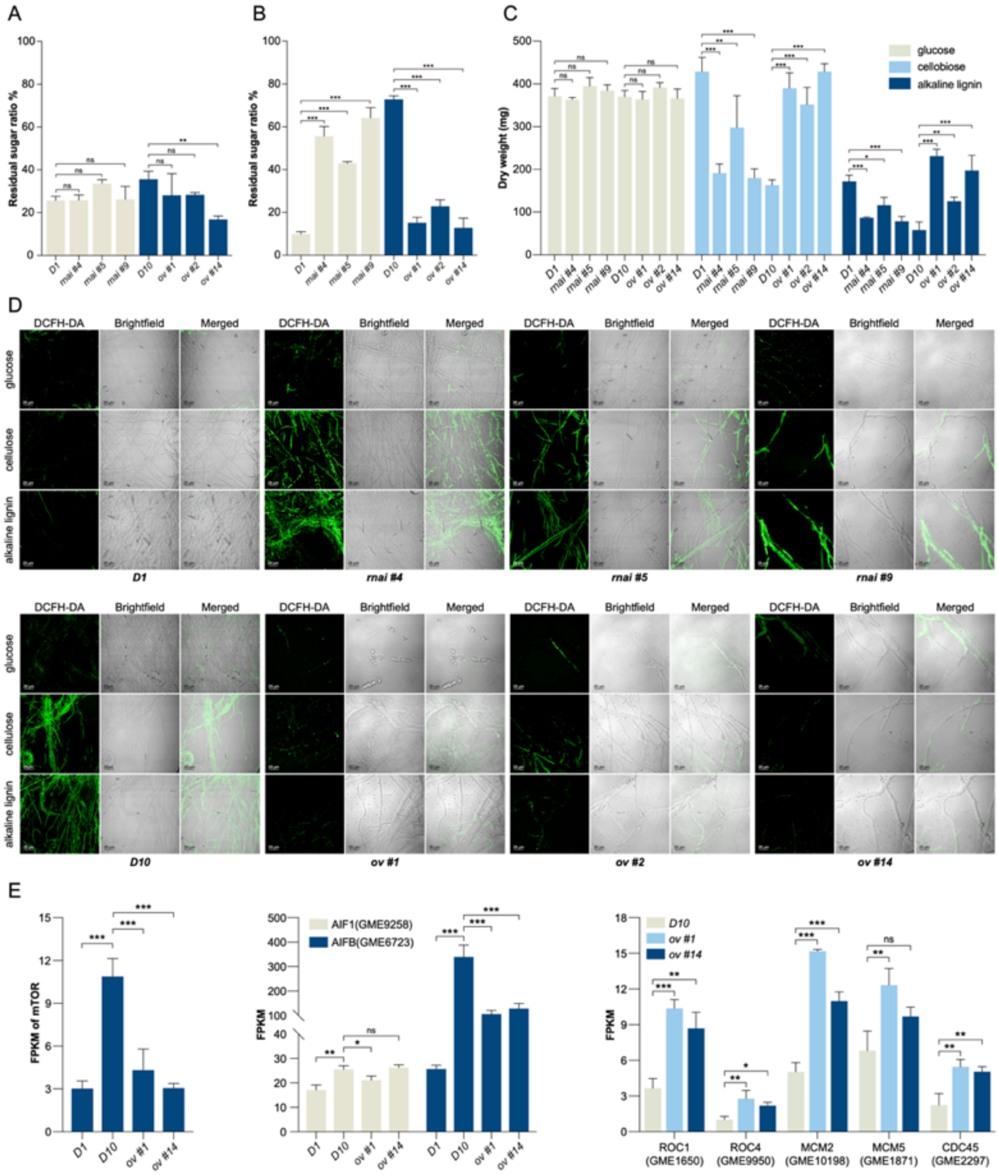

Fig. S17 The physiological metabolism and gene expression of *Thipk1*-silenced and -overexpressed transformants grown under different carbon sources. (A and **B)** Residual reducing sugar content of *T. hirsuta* AH28-2 *D1*, *D10*, and *Thipk1*-silenced and -overexpressed transformants after 120 h of cultivation in the liquid medium with glucose (A) and cellobiose (B) as carbon sources, respectively. **(C)** The mycelial dry weight after 120 h of cultivation in the liquid medium with glucose, cellobiose, and alkaline lignin as carbon sources. **(D)** ROS levels indicated by staining with DCFH-DA and imaged under a confocal laser scanning microscope. Scale bar, 10 μm. (**E**) Transcriptomic analysis revealed the effect of *Thipk1* overexpression on the expression levels of *mTOR*, apoptosis-inducing factor (*AIF*), and several cell cycle-related genes (*ROC*, *MCM,* and *CDC45*).

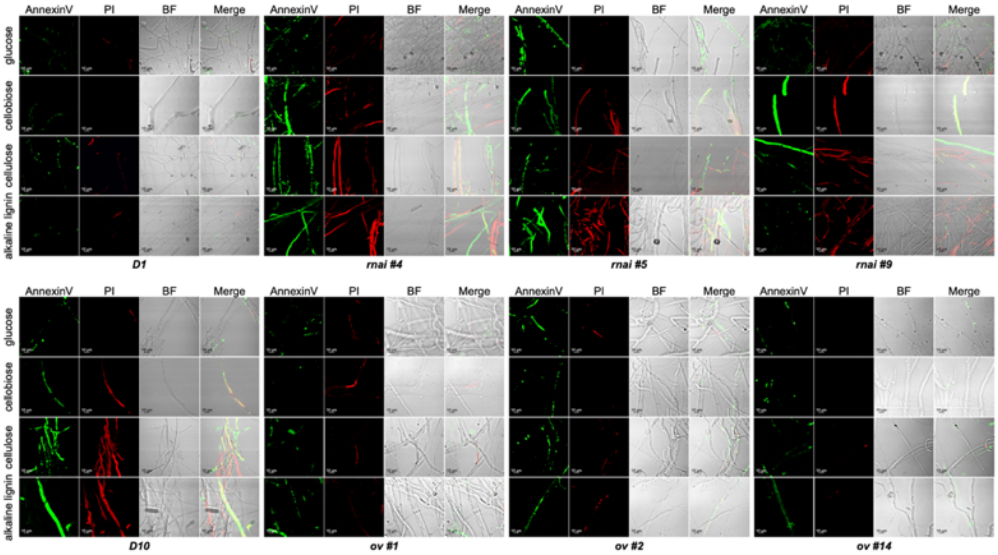

Fig. S18 Apoptosis levels of *Thipk1*-silenced and -overexpressed transformants under different carbon sources. Mycelia of *T. hirsuta* AH28-2 *D1*, *D10*, and *Thipk1*-silenced and -overexpressed transformants grown on a solid medium with glucose, cellobiose, or alkaline lignin as a carbon source were co-stained with Annexin V–PI and imaged under a confocal laser scanning microscope. Scale bar, 10 μm.

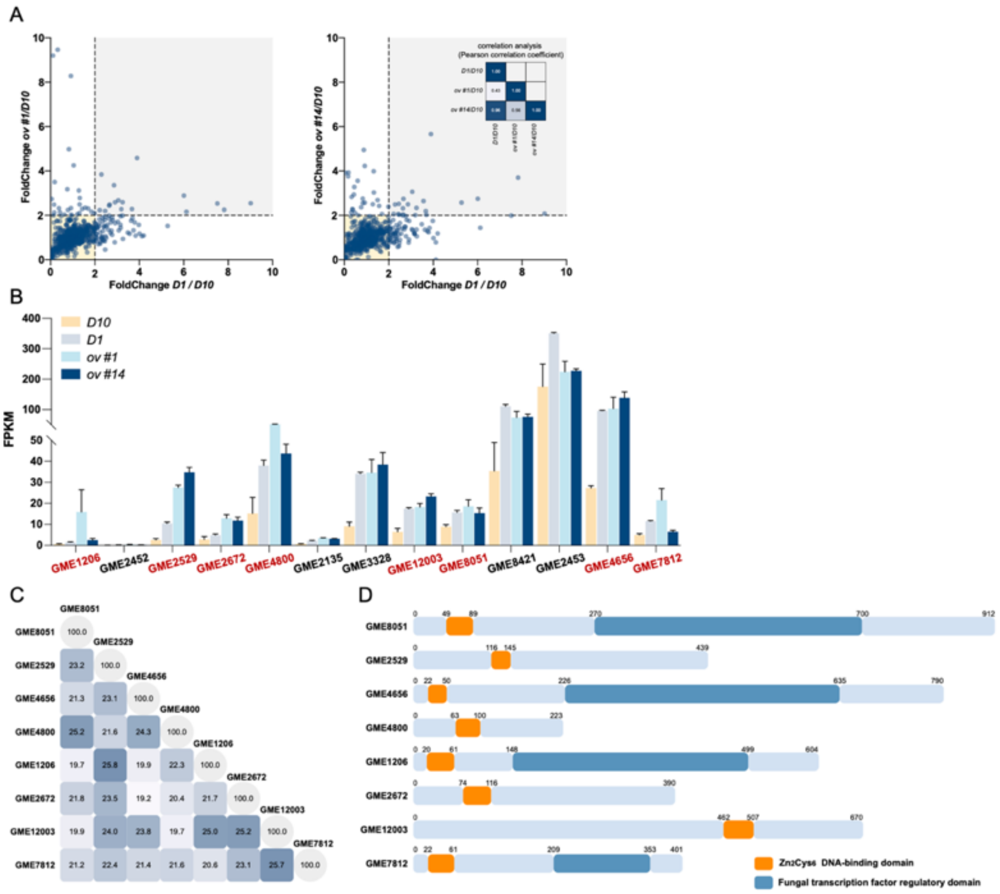

Fig. S19 Transcription factor enrichment analysis of *T. hirsuta* AH28-2 in response to *Thipk1* overexpression. (A) The scatter plots show the correlation of the expression levels of all transcription factor genes between the *D1* vs. *D10* group (X-axis) and the *ov #1* (or *ov #14*) vs. *D10* group (Y-axis). The gray area indicates that the transcription levels of this transcription factor were up-regulated by more than twofold in the *D1* and *Thipk1*-overexpressed transformants compared to *D10*. **(B)** FPKM of 13 Zn_2Cys_6 transcription factors in *T. hirsuta* AH28-2 *D1*, *D10*, and *Thipk1*-overexpressed transformants *ov #1* and *ov #14*. Eight Zn_2Cys_6 transcription factors in red were selected for further research. **(C)** Comparison of sequence identities among eight Zn_2Cys_6 transcription factors. **(D)** Analysis of conserved domains of eight Zn_2Cys_6 transcription factors. The orange region represents the Zn_2Cys_6 DBD, and the dark blue region represents the fungal regulatory domain. The numbers indicate the location of the domain within the protein.

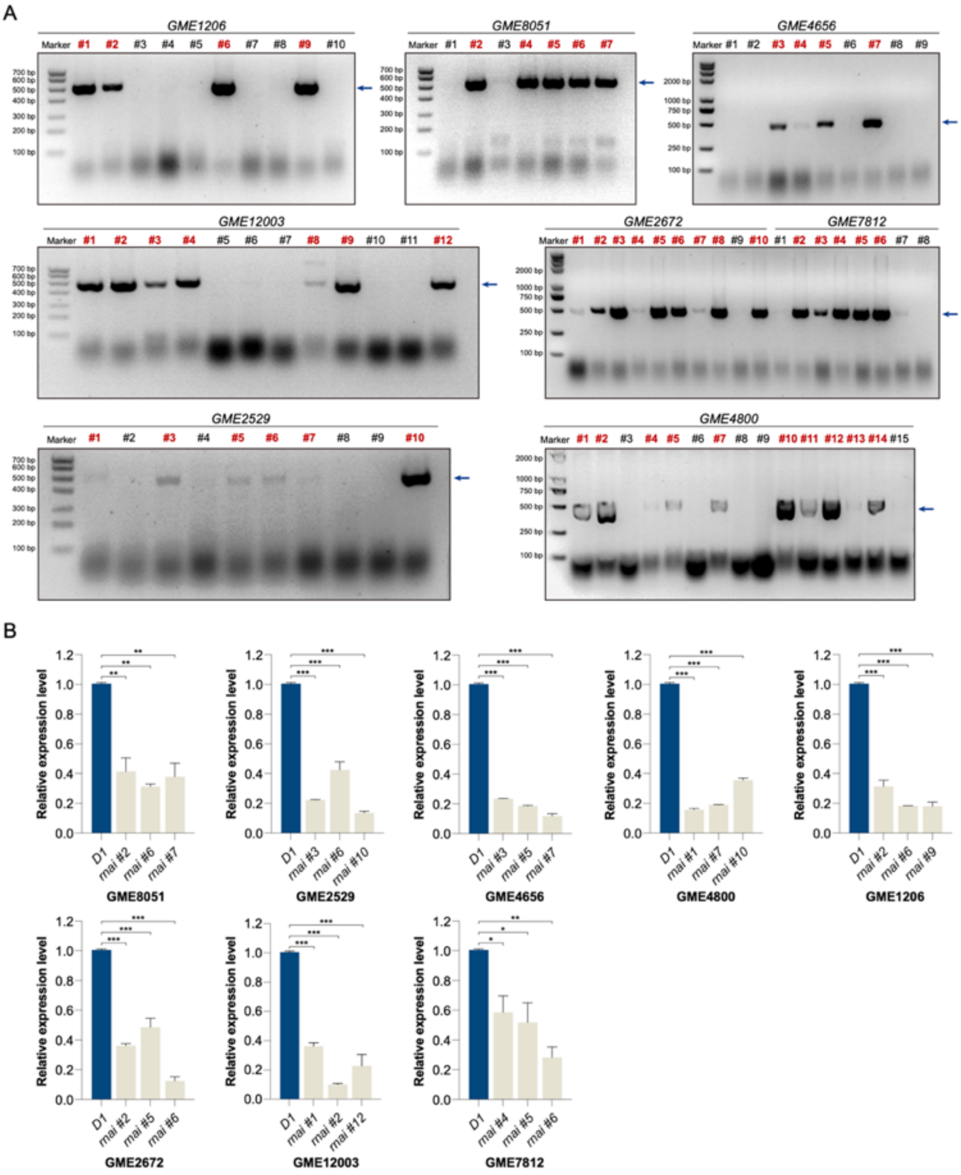

Fig. S20 Verification of Zn_2Cys_6 transcription factor-silenced transformants. (A) The genomic PCR verification. The blue arrow shows the specific target band, which was verified by sequencing. **(B)** qRT-PCR analysis of the down-regulation of target gene transcription levels in Zn_2Cys_6 transcription factor-silenced transformants.

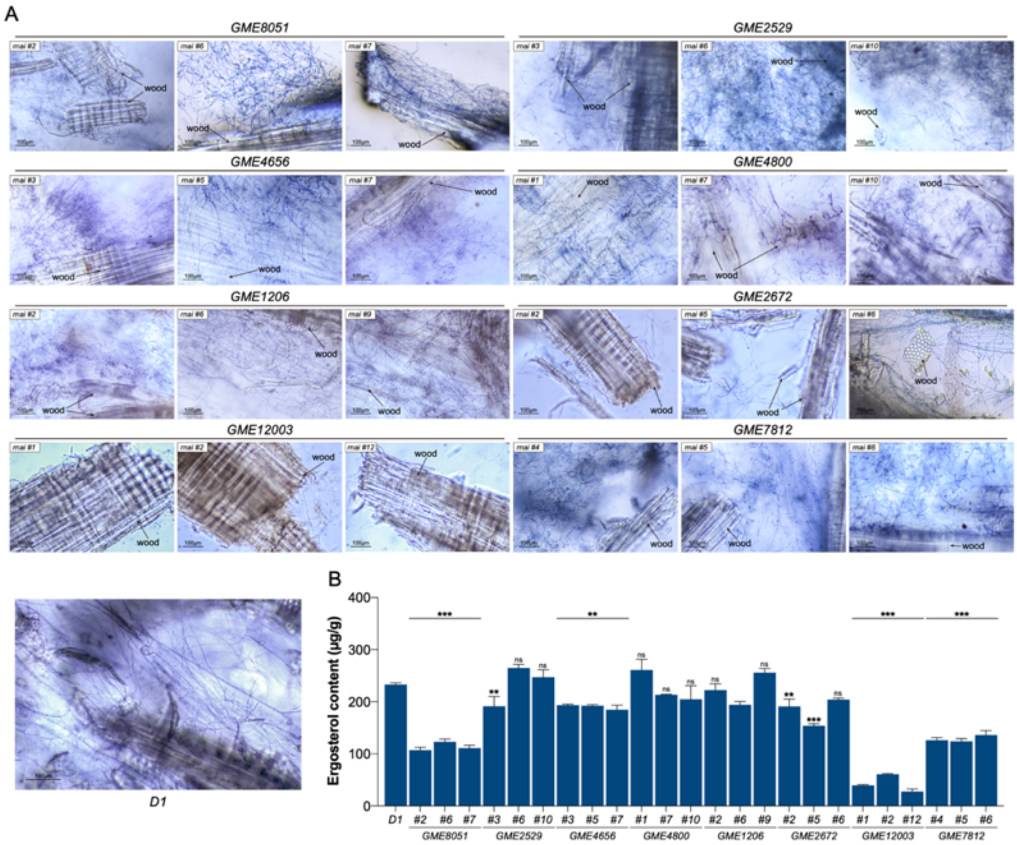

Fig. S21 Phenotypes of different Zn_2Cys_6 genes silencing transformants growing on poplar. (A) Optical microscope images of different Zn_2Cys_6 genes silencing transformants grown on poplar wood. The strains appear blue after staining. Scale bar, 100 μm. **(B)** The bar chart shows the ergosterol content of different Zn_2Cys_6 genes silencing strains grown on poplar wood for 40 d.

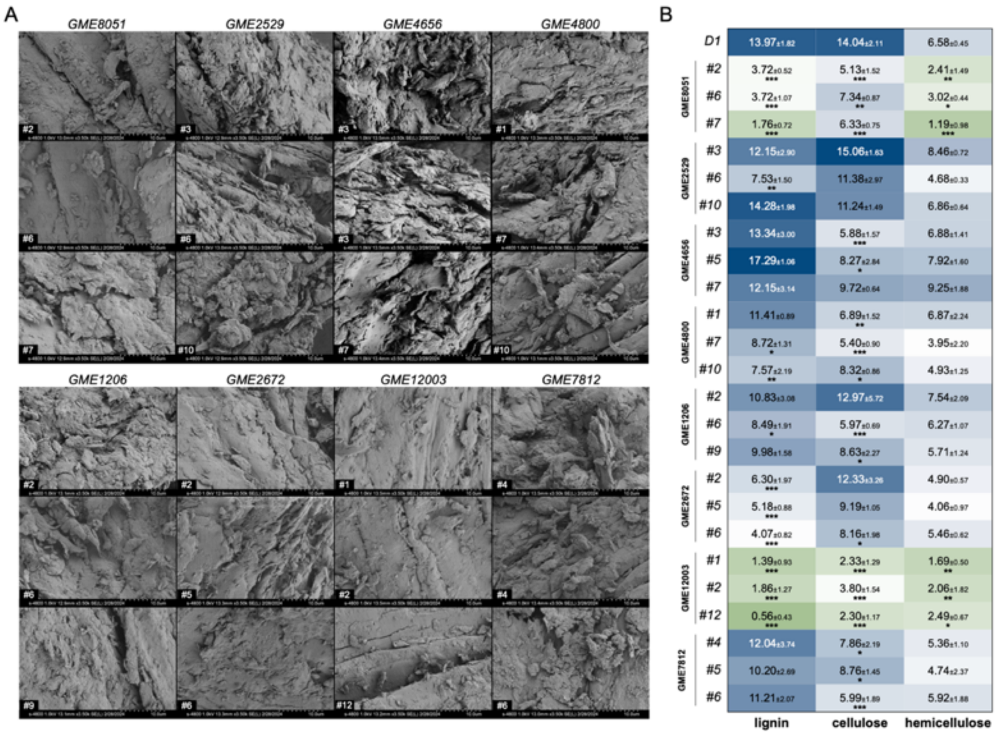

Fig. S22 Characterization of the ability of Zn_2Cys_6 gene-silenced transformants to degrade poplar. (A) SEM images of poplar wood after 40 d of treatment with *T. hirsuta* AH28-2 *D1* and Zn_2Cys_6 gene-silenced transformants. Scale bar, 10 µm. **(B)** The degradation rate of lignocellulose in poplar wood after 40 d of treatment. The "degradation rate" represented the extent of reduction in lignin, cellulose, or hemicellulose after cultivation relative to their respective initial amounts in the non-inoculated poplar medium.

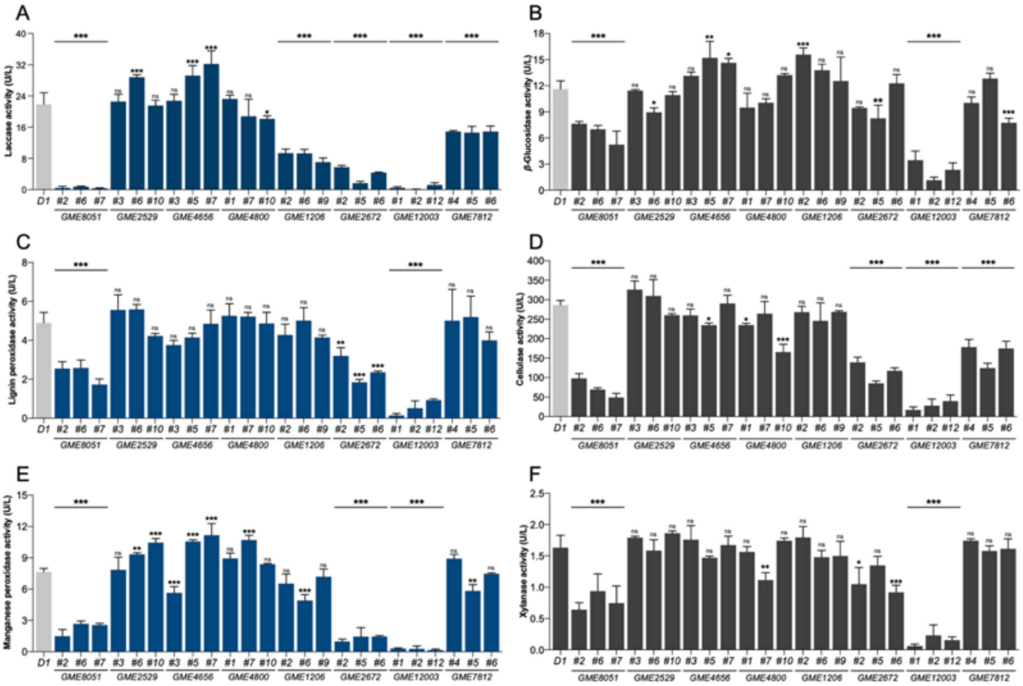

Fig. S23 lignocellulolytic enzyme activities of Zn_2Cys_6-silenced transformants grown on poplar wood. The activities of laccase (B), β-glucosidase (C), lignin peroxidase (D), cellulase (E), manganese peroxidase (F) and xylanase (G) were detected in the *D1* and Zn_2Cys_6 gene-silenced transformants after 40 d of cultivation in liquid poplar wood medium.

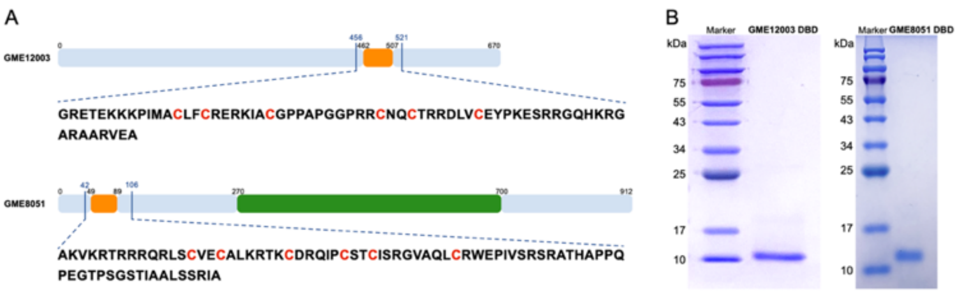

Fig. S24 Heterologous expression and purification of the Zn_2Cys_6 transcription factor GME12003 and GME8051 DBD. (A) GME12003 and GME8051 DBD sequences. The red cysteines indicate the conserved cysteine motif of Zn_2Cys_6. **(B)** The purified GME12003 and GME8051 DBD proteins.

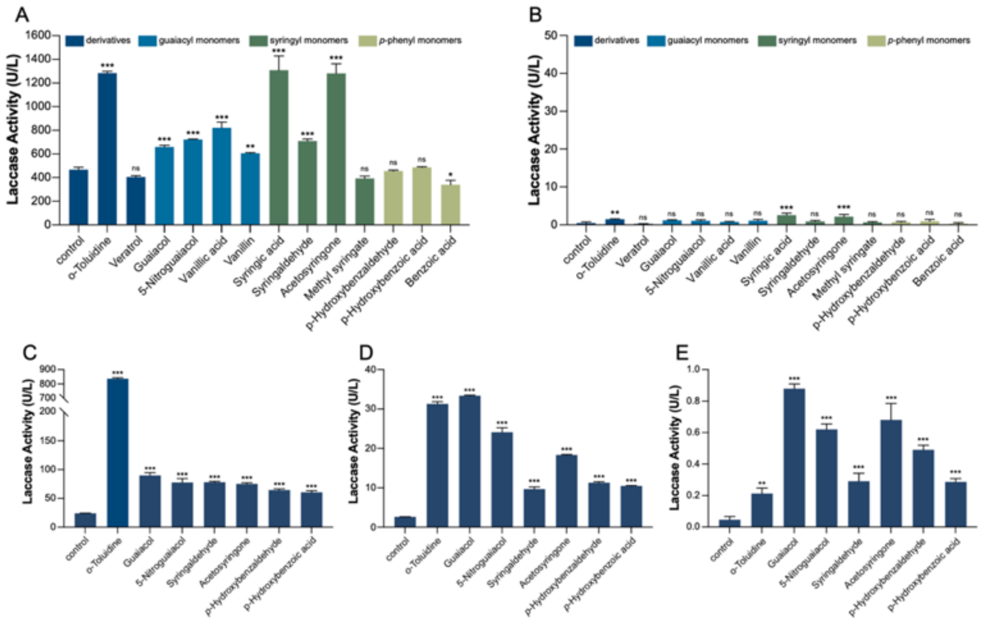

Fig. S25 Effects of different lignin monomer compounds and derivatives on laccase production in *T. hirsuta* AH28-2. (A and B) Laccase activity of *T. hirsuta* AH28-2 *D1* (A) and *D10* (B) cultures after 48 h of treatment. **(C-E)** Determination of laccase activity of *Trametes versicolor* (C), *Pleurotus ostreatus* (D), and *Schizophyllum commune* (E) after treatment for 48 h.

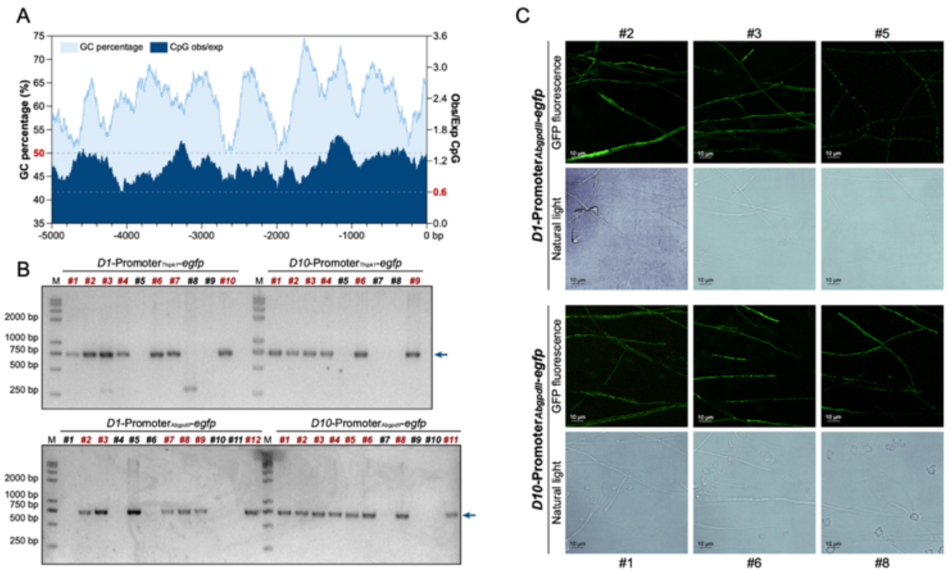

Fig. S26 Verification of the transcriptional strength of *Thipk1* promoter in cultures *D1* and *D10*. (A) GC percentage and the ratio of observed (Obs) to expected (Exp) CpG dinucleotides in 5, 000 bp upstream of the *Thipk1* start codon (ATG). GC percentage is defined as the ratio of G or C per 100 bp. Obs is the actual number of CpG dinucleotides in a given region of the genome. Exp is the number of CpG dinucleotides expected based on the frequency of C and G nucleotides in the surrounding sequence. A CpG island is typically defined as a region where the Obs/Exp ratio is greater than a certain threshold (often 0.6 or higher) and a GC percentage higher than 50%. **(B)** The *Thipk1* and *AbgpdII* promoter reporter clones were verified by genomic PCR using *egfp* primers. The blue arrows indicate the specific target bands, which were verified by sequencing. **(C)** eGFP fluorescence intensity of the *AbgpdII* promoter transcriptional activity reporter clones under laser confocal microscopy. Scale bar, 10 μm.

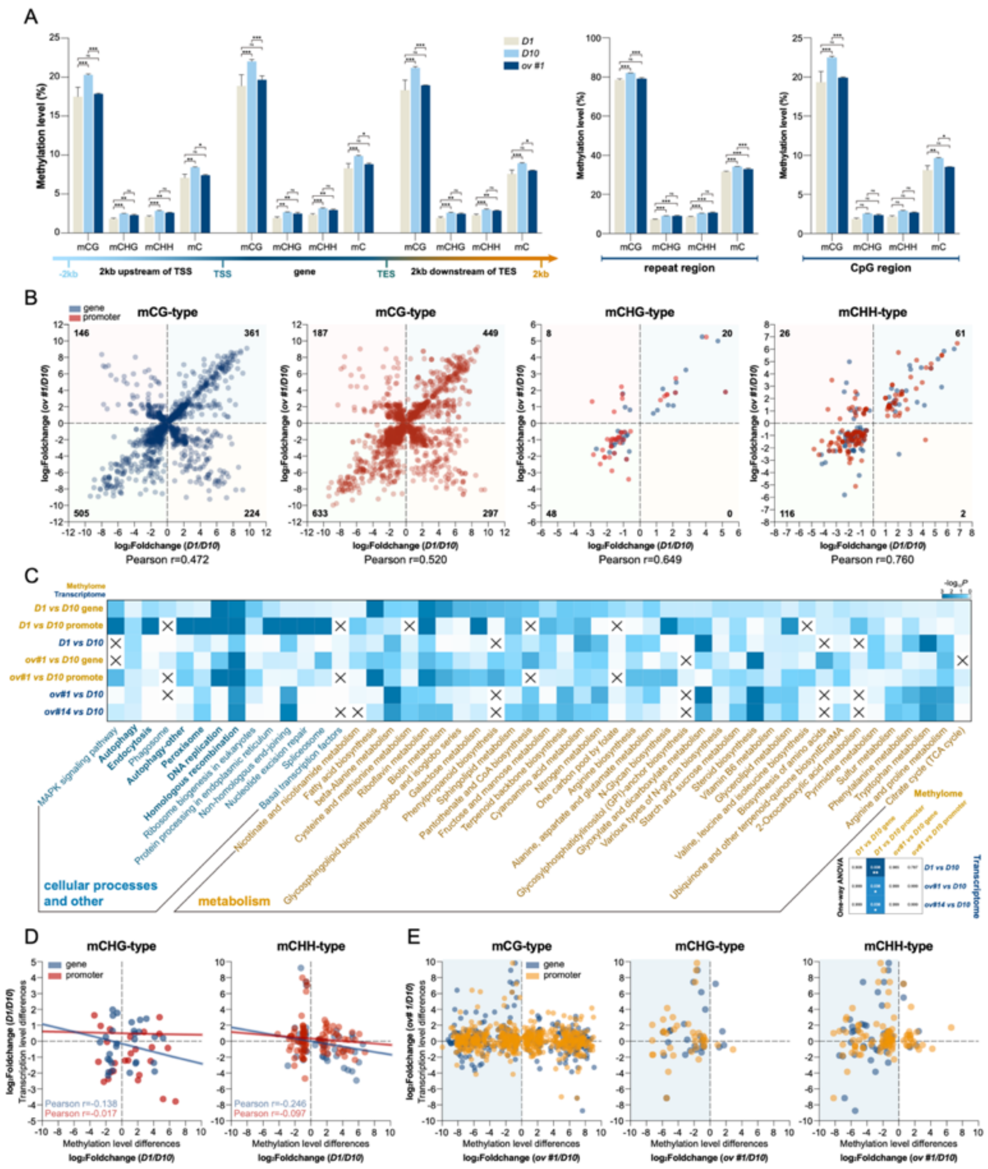

Fig. S27 *Th*ipk1 affects the cytosine methylation pattern of the *T. hirsuta* AH28-2 genome. (A) Cytosine methylation rates in the promoter (2000 bp upstream of the start codon), genes, terminator (2000 bp downstream of the stop codon), repeat, and CpG regions of *T. hirsuta* AH28-2 *D1*, *D10*, and *ov #1* genomes. **(B)** The scatter plots show the correlation of the regional cytosine methylation rate fold change of all mCG/mCHG/mCHH-related genes (blue) or promoters (red) within the gDMR in the *D1* vs *D10* group (X-axis) and *ov #1* vs *D10* (Y-axis). The gene numbers located within each quadrant. The Pearson correlation coefficient for each result was calculated. **(C)** KEGG enrichment analysis of the top 50 mCG-related genes with the gDMR in the *D1* vs. *D10* group, and the *P* values of these KEGGs in other methylomes and transcriptomes. “×” indicates that the KEGG pathway is not enriched

in this group. Each point represents the -logP value corresponding to the pathway enrichment in a specific omics dataset, and the variance across groups is evaluated using one-way ANOVA. **(D)** Correlation between the fold change in regional cytosine methylation rate (X-axis) and transcriptome expression difference (Y-axis) of *D1* vs. *D10* mCHG/mCHH-related genes (blue) or promoters (red) within the gDMR in the KEGG pathway. **(E)** Correlation between the fold change in regional cytosine methylation rate (X-axis) and transcriptome expression difference (Y-axis) of *ov #1* vs. *D10* mCHG/mCHH-related genes (blue) or promoters (orange) within the gDMR in the KEGG pathway.

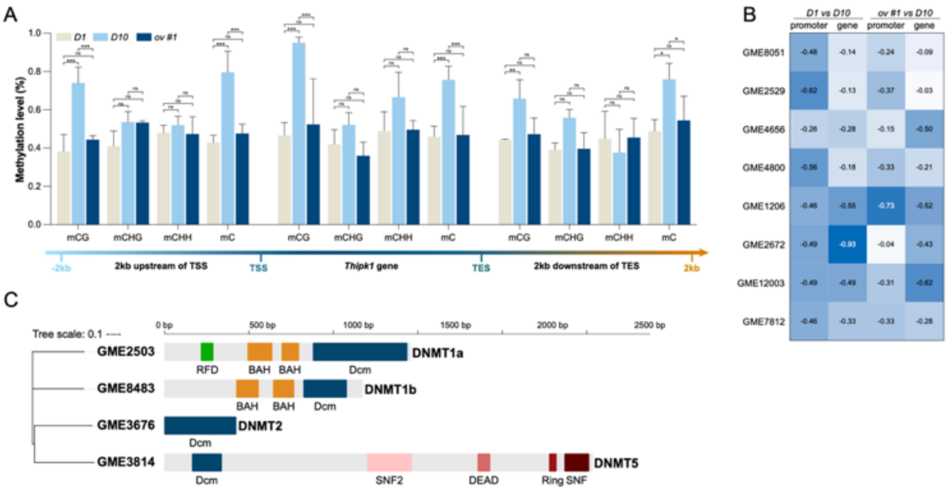

Fig. S28 *T. hirsuta* AH28-2 DNA methyltransferase and genomic DNA methylation patterns. (A) Cytosine methylation rates in the promoter (2000 bp upstream of the start codon), intragenic and terminator (2000 bp downstream of the stop codon) regions of the *Thipk1* gene in *T. hirsuta* AH28-2 *D1*, *D10,* and *ov*#1 cultures. **(B)** Phylogenetic tree analysis and conserved domain structure of DNA methyltransferase of transcription factors. **(C)** Phylogenetic tree analysis and conserved domain structure of DNA methyltransferase of *T. hirsuta* AH28-2. **RFD**, replication foci domain; **BAH**, bromo-adjacent homology; **Dcm**, DNA-cytosine methyltransferase.

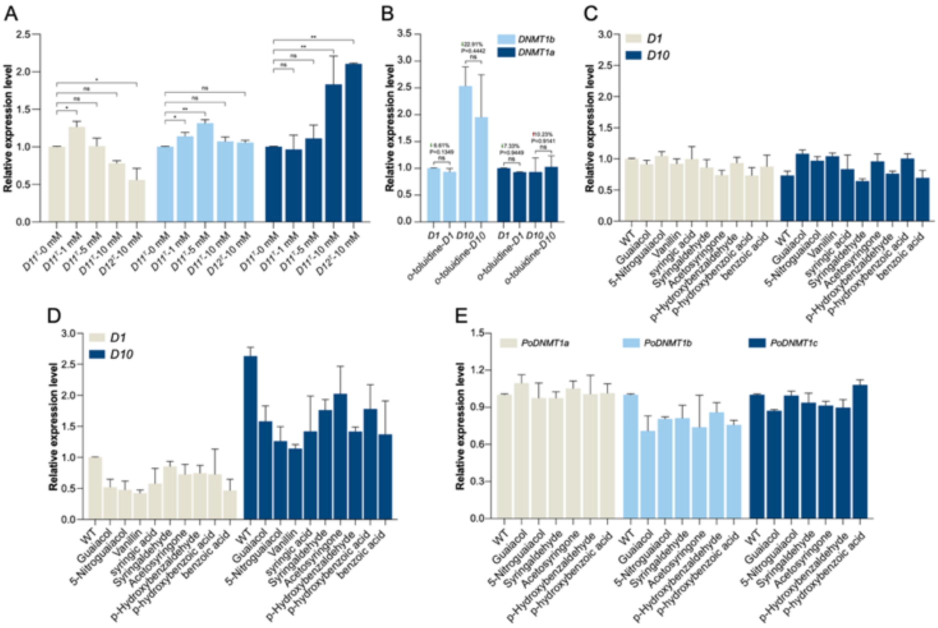

Fig. S29 *DNMT1* responds to lignin monomer compounds. (A) *DNMT1a*, *DNMT1b*, and *Thipk1* transcript levels of *T. hirsuta* AH28-2 *D11*^{T} and *D12*^{T} cultures. **(B)** The transcriptional levels of *DNMT1a* and *DNMT1b* in *T. hirsuta* AH28-2 *D1* and *D10* cultures after 24 h of exposure to *o*-toluidine. **(C and D)** Transcription levels of *DNMT1a* (C) and *DNMT1b* (D) in *T. hirsuta* AH28-2 *D1* and *D10* after exposure to lignin monomers. (E) Transcription levels of *DNMT1a*, *DNMT1b,* and *DNMT1c* in *P. ostreatus* after exposure to lignin monomers.

Supplementary Tables

Table S1. Primers used in this study. The silencing or overexpression vectors, protein heterologous expression vectors and primers for qRT-PCR are shown in the following table.

Name	Sequence	Intention
F-*gapdh*-q	GCCGCTTCAAGGGCAAAGTC	qRT-PCR primer of *gapdh*
R-*gapdh*-q	TGTAGTCGGCACCAACGGA	qRT-PCR primer of *gapdh*
F-*Thipk1*-q	CGGTCGAGATCAAGCCGAAA	qRT-PCR primer of *Thipk1*
R-*Thipk1*-q	GTCTTCTTCAGTTGCGCGTG	qRT-PCR primer of *Thipk1*
F-*GME4803*-q	CCAACCACACGTTCAGCATC	qRT-PCR primer of *GME4803*-Lac
R-*GME4803*-q	ATCCTGGTCGGCATTCAACA	qRT-PCR primer of *GME4803*-Lac
F-*GME4972*-q	CATTACCGACGCCGCCATT	qRT-PCR primer of *GME4972*-Lac
R-*GME4972*-q	AGTTGGTGCCTGCCTGGAA	qRT-PCR primer of *GME4972*-Lac
F-*GME7109*-q	TTTCCCGCCTAACGCAGACA	qRT-PCR primer of *GME7109*-Lac
R-*GME7109*-q	ATCGCACGAGAGCGAGACAA	qRT-PCR primer of *GME7109*-Lac
F-*GME6111*-q	GTGGCAGTCCTTCGTCAACAA	qRT-PCR primer of *GME6111*-MnP
R-*GME6111*-q	TCGGAGCAGTCGATGAGGTC	qRT-PCR primer of *GME6111*-MnP
F-*GME9857*-q	TGTTCGACACGCAGTTCTTCAT	qRT-PCR primer of *GME9857*-MnP
R-*GME9857*-q	TGGAGCTTCGCCTGGTTGT	qRT-PCR primer of *GME9857*-MnP
F-*GME6075*-q	ACGGCTCCATCGCCATCTT	qRT-PCR primer of *GME6075*-MnP
R-*GME6075*-q	TCCGCAGTGGTGAGGTTGT	qRT-PCR primer of *GME6075*-MnP
F-*GME3355*-q	GCGACGACATCCAGAAGAACC	qRT-PCR primer of *GME3355*-MnP
R-*GME3355*-q	GTTGGCGTGGAAGTTGGTCTC	qRT-PCR primer of *GME3355*-MnP
F-*GME507*-q	CCAGGTCTTCATCGAGGTCC	qRT-PCR primer of *GME507*-MnP
R-*GME507*-q	GTTCACGAATGACTGCCACTC	qRT-PCR primer of *GME507*-MnP
F-*GME7667*-q	TCTCCAGTCCGACTTCCTCATC	qRT-PCR primer of *GME7667*-VP
R-*GME7667*-q	CACCGTCTGCTCGAACTTCTG	qRT-PCR primer of *GME7667*-VP
F-*GME12053*-q	GCTATCGGCATCTCGTCTACAC	qRT-PCR primer of *GME12053*-VP
R-*GME12053*-q	CTCGTCCACTCCAACATTCTCC	qRT-PCR primer of *GME12053*-VP
F-*GME10557*-q	GCTCCTCAAGGGCACACTCT	qRT-PCR primer of *GME10557*-VP
R-*GME10557*-q	GCCATCGCCTTCGTGAACTT	qRT-PCR primer of *GME10557*-VP
F-*GME10572*-q	ATCTCCAGCAGAACCTCTTCCA	qRT-PCR primer of *GME10572*-LiP
R-*GME10572*-q	ACTTGCCCTGTGCCTCCAT	qRT-PCR primer of *GME10572*-LiP
F-*GME261*-q	GTTCGTCTTCCAGGTGCTCAC	qRT-PCR primer of *GME261*-LiP
R-*GME261*-q	GCCTGCTCGATGTCGTTGT	qRT-PCR primer of *GME261*-LiP
F-*GME10569*-q	GCAAGGCAAGTTCGGTGGT	qRT-PCR primer of *GME10569*-LiP
R-*GME10569*-q	TGGAGGAAGTCAGCGTGTGA	qRT-PCR primer of *GME10569*-LiP
F-*GME10575*-q	GACGGCTCGATCTCAATCTTCC	qRT-PCR primer of *GME10575*-LiP
R-*GME10575*-q	TCAGCGTGCGAGAGGTTGT	qRT-PCR primer of *GME10575*-LiP
F-*GME10307*-q	TGACCTTCCACGACGCCAT	qRT-PCR primer of *GME10307*-LiP
R-*GME10307*-q	TGAAGTCAGCAACGCCAATGT	qRT-PCR primer of *GME10307*-LiP
F-*GME3358*-q	CGGTGTTGCTGACTTCATCCA	qRT-PCR primer of *GME3358*-LiP
R-*GME3358*-q	CAGGCTGAGTCGCATCCTTG	qRT-PCR primer of *GME3358*-LiP
F-*GME5668*-q	ATCACTGGCGGAGGTAGCACAA	qRT-PCR primer of *GME5668*-AA9
R-*GME5668*-q	CGTTGCACGAGAAGACCGAAGG	qRT-PCR primer of *GME5668*-AA9
F-*GME11891*-q	ATGGCGAGGTGCGATATGGA	qRT-PCR primer of *GME11891*-GH3
R-*GME11891*-q	TGATGCGGTCAGCGTTATGTC	qRT-PCR primer of *GME11891*-GH3
F-*GME5585*-q	ACGAGAAGAAGCGGCAGTGG	qRT-PCR primer of *GME5585*-GH3
R-*GME5585*-q	GCTCAGGCGGTCCTTGTTGT	qRT-PCR primer of *GME5585*-GH3
F-*GME6933*-q	TGAACAGGTCGTCGCAGTACA	qRT-PCR primer of *GME6933*-GH5
R-*GME6933*-q	GCGTCGGTAGAGCCAGAGTT	qRT-PCR primer of *GME6933*-GH5
F-*GME10182*-q	AGCACGGTGACGAAGGAGGA	qRT-PCR primer of *GME10182*-GH5
R-*GME10182*-q	CCGAGGTTCACGCCGTTGAT	qRT-PCR primer of *GME10182*-GH5
F-*GME4680*-q	CGCTCTACTTCGTCGAGATGG	qRT-PCR primer of *GME4680*-GH7

R-*GME4680*-q	CGCCGTTGATGAACTTGATGTC	qRT-PCR primer of *GME4680*-GH7
F-*GME4972*-q	ACCCATCCTCATCCTGCGACA	qRT-PCR primer of *GME4972*-GH16
R-*GME4972*-q	GCGTAGTTCGTGCCGTTGTTG	qRT-PCR primer of *GME4972*-GH16
F-*GME7709*-q	GACATCCGCATGACGCTTCCT	qRT-PCR primer of *GME7709*-GH10
R-*GME7709*-q	TCCCACAGAGTAACGCCCACA	qRT-PCR primer of *GME7709*-GH10
F-*GME209*-q	ACGCTGTCCGCAAGGTCAT	qRT-PCR primer of *GME209*-GH47
R-*GME209*-q	ATGGAACGGCTGCTCTGGTT	qRT-PCR primer of *GME209*-GH47
F-*GME5907*-q	GGTCCGCATCCAGAGCAACAA	qRT-PCR primer of *GME5907*-GH16
R-*GME5907*-q	GTTGGGCTCGATGTCGTTGGT	qRT-PCR primer of *GME5907*-GH16
F-*GME44*-q	CGCTGGACATATCTACGGCAAT	qRT-PCR primer of *GME44*-GH2
R-*GME44*-q	GACGGTGGAGTTGAAGGAGAAG	qRT-PCR primer of *GME44*-GH2
F-*GME1030*-q	ACCAACCAGTTCGCCAGCAG	qRT-PCR primer of *GME1030*-GH71
R-*GME1030*-q	ACGGCACGAAGTGGACCTTG	qRT-PCR primer of *GME1030*-GH71
F-*GME6564*-q	GGAGCACGCCCTTGGTATCAA	qRT-PCR primer of *GME6564*-GH71
R-*GME6564*-q	GTCGCAGAAGTCGCCACAGT	qRT-PCR primer of *GME6564*-GH71
F-*GME7187*-q	ACCTGGTTTCTGCACAACAAGT	qRT-PCR primer of *GME7187*-GH9
R-*GME7187*-q	AGCACCTCCACGATGACCTC	qRT-PCR primer of *GME7187*-GH9
F-*GME3837*-q	GGATGTGTAGCCTGTGGTGTC	qRT-PCR primer of *GME3837*-GH45
R-*GME3837*-q	CGGTGACCTTGACGACGATC	qRT-PCR primer of *GME3837*-GH45
F-*GME3718*-q	CGTCCTCGTCGTGAGAATGTC	qRT-PCR primer of *GME3718*-GH38
R-*GME3718*-q	GTTCGCCGTCATCGTCAATCT	qRT-PCR primer of *GME3718*-GH38
F-*GME8051*-q	TCCAGGTTTCTCGCACATCCC	qRT-PCR primer of *GME8051*-TF
R-*GME8051*-q	GGTCGCATTTCGTTCGCTTGA	qRT-PCR primer of *GME8051*-TF
F-*GME2529*-q	CCTCCAACAAGCAGCGGACA	qRT-PCR primer of *GME2529*-TF
R-*GME2529*-q	AAGGCGAGGTGCGTACAGTG	qRT-PCR primer of *GME2529*-TF
F-*GME4656*-q	GCCCGTCTCGTGTGCTGAAT	qRT-PCR primer of *GME4656*-TF
R-*GME4656*-q	TCCTCGACATCCGCTACTGCT	qRT-PCR primer of *GME4656*-TF
F-*GME4800*-q	CAGCAACACGCCCAACAACTC	qRT-PCR primer of *GME4800*-TF
R-*GME4800*-q	AGAGACTCGCTGCACCTGGT	qRT-PCR primer of *GME4800*-TF
F-*GME1206*-q	GGTGCTCGTCGCCAACTACTT	qRT-PCR primer of *GME1206*-TF
R-*GME1206*-q	CCGTCGAACTCCGCATCTTGT	qRT-PCR primer of *GME1206*-TF
F-*GME2672*-q	TTCGCTCAGGGCAGCTCTCA	qRT-PCR primer of *GME2672*-TF
R-*GME2672*-q	ACGAGGAAGCCGCCATTGTG	qRT-PCR primer of *GME2672*-TF
F-*GME12003*-q	ATGTGCGAGAGCAAGCAGGA	qRT-PCR primer of *GME12003*-TF
R-*GME12003*-q	AAGGGATGGACGGTTGAGGG	qRT-PCR primer of *GME12003*-TF
F-*GME7812*-q	ATCGTGCTCTCATGCGTGGA	qRT-PCR primer of *GME7812*-TF
R-*GME7812*-q	ATTGTGCGGCGGAGTTGCT	qRT-PCR primer of *GME7812*-TF
F-*GME10978*-q	TGTGGTTGAGGAGGAGATGA	qRT-PCR primer of *GME10978*-NDUFV1
R-*GME10978*-q	AGCGTCCTTCAGCGAGTCA	qRT-PCR primer of *GME10978*-NDUFV1
F-*GME11812*-q	CCGCCGCATTTGGTCTGAA	qRT-PCR primer of *GME11812*-SDH1
R-*GME11812*-q	CGTTCCTTCGCACTCGTCTT	qRT-PCR primer of *GME11812*-SDH1
F-*GME11737*-q	GCTGTCACCTTCGTCGTCAA	qRT-PCR primer of *GME11737*-QCR2
R-*GME11737*-q	ACCGCTTGTCCGTGCTCTTA	qRT-PCR primer of *GME11737*-QCR2
F-*GME9085*-q	TTGAGGCGGGCAAGAAGTTC	qRT-PCR primer of *GME9085*-ATP5L
R-*GME9085*-q	TCACGGAAGACAGCGAAGTTG	qRT-PCR primer of *GME9085*-ATP5L
F-*GME9830*-q	CTCACCAAGGTCCAGTCCGA	qRT-PCR primer of *GME9830*-ATP5o
R-*GME9830*-q	GAGAGCACGACGAACAGGTT	qRT-PCR primer of *GME9830*-ATP5o
F-*GME2973*-q	CGTCGTCCTCCACAGCATATTC	qRT-PCR primer of *GME2973*-mTOR
R-*GME2973*-q	TCCTGCGTGTTGACTATCTCCA	qRT-PCR primer of *GME2973*-mTOR
F-*GME11216*-q	CGAACGAGGAGTACCGCTACA	qRT-PCR primer of *GME11216*-SPT
R-*GME11216*-q	GCCGAGGCAGATTACAAGGAT	qRT-PCR primer of *GME11216*-SPT
F-*GME2842*-q	AGCAGGAGTCCAACAACTACCA	qRT-PCR primer of *GME2842*-ATG13
R-*GME2842*-q	GCACATAGTTCGGCGGCATT	qRT-PCR primer of *GME2842*-ATG13
F-*GME11418*-q	GCAGCAGACACAGATGGAACA	qRT-PCR primer of *GME11418*-Vam7
R-*GME11418*-q	TCCGCAACCTCCTGGTGAAT	qRT-PCR primer of *GME11418*-Vam7
F-*GME6723*-q	AGTAGGTGGCGGTGCTGTT	qRT-PCR primer of *GME6723*-AIFB
R-*GME6723*-q	GGTAGGTGGAGTTGACGAGGA	qRT-PCR primer of *GME6723*-AIFB

F-*GME9258*-q	CGTCTCGGCGACAAGATACAC	qRT-PCR primer of *GME9258*-AIF1
R-*GME9258*-q	TTGAACGAATGGAGAGCGGTAG	qRT-PCR primer of *GME9258*-AIF1
F-*GME6167*-q	CGGCAGTCGCTGTGTTATGAT	qRT-PCR primer of *GME6167*-Metacaspase
R-*GME6167*-q	ATGTCTGTGCTGGCATTGGAAT	qRT-PCR primer of *GME6167*-Metacaspase
F-*GME6436*-q	GGCTACAAGGCACGCAACAT	qRT-PCR primer of *GME6436*-Metacaspase
R-*GME6436*-q	ATCTCGTCTCCGTTCCTGTCTT	qRT-PCR primer of *GME6436*-Metacaspase
F-*GME7294*-q	ATCAACATCGCCTCGTCAAGT	qRT-PCR primer of *GME7294*-PHO85
R-*GME7294*-q	CGCACTTCGCCTTCTTCGT	qRT-PCR primer of *GME7294*-PHO85
F-*GME7582*-q	CGACGCCGAGAAGGACAAGAA	qRT-PCR primer of *GME7582*-MIOX
R-*GME7582*-q	TCGCCTCCCAGATGCCCATT	qRT-PCR primer of *GME7582*-MIOX
F-*GME3876*-q	CGTCCCGCTCGCAGTTATCAT	qRT-PCR primer of *GME3876*-CDIPT
R-*GME3876*-q	CGCCGTGTTGACCTTGCTGA	qRT-PCR primer of *GME3876*-CDIPT
F-*GME2250*-q	TCCTCCACGAGTGCCATGAGA	qRT-PCR primer of *GME2250*-PIP5K
R-*GME2250*-q	TATGCGGTGGTGTGGCTGTG	qRT-PCR primer of *GME2250*-PIP5K
F-*GME10005*-q	CGTCAACGAGCCCGAGCATA	qRT-PCR primer of *GME10005*-PLC
R-*GME10005*-q	TTCGAGAGCAGAACGCAGGT	qRT-PCR primer of *GME10005*-PLC
F-*GME2503*-q	GCGAACCAGAGGACTGCCATT	qRT-PCR primer of *GME2503*-*Th*DNMT1a
R-*GME2503*-q	CAGGAGCCGTTGTGCATCGT	qRT-PCR primer of *GME2503*-*Th*DNMT1a
F-*GME8483*-q	TCGCCAACCGACACCTTACC	qRT-PCR primer of *GME8483*-*Th*DNMT1b
R-*GME8483*-q	GCCGACGACCTTCAGATGCT	qRT-PCR primer of *GME8483*-*Th*DNMT1b
F-*GME3814*-q	CCGCTGCTTGCCTTGAACCT	qRT-PCR primer of *GME3814*-*Th*DNMT5
R-*GME3814*-q	CCGCTCGATGTACGCTTGCT	qRT-PCR primer of *GME3814*-*Th*DNMT5
F-ThIPK1	ggtggatccATGGAAAACGACGCGCCC	heterologous expression of ThIPK1
R-ThIPK1	gtgctcgagCTACGGCAGCGGCACAGC	heterologous expression of ThIPK1
F-ThIPK1-ov	ATGGAAAACGACGCGCCCAT	*Thipk1* overexpression vector
R-ThIPK1-ov	CTACGGCAGCGGCACAGCAC	*Thipk1* overexpression vector
F-ThIPK1-rani	GCAAGGGCCCCACGCCCGCG	construction of *Thipk1* silencing vector
R-ThIPK1-rani	GGGGAGAGGTAGGCAGGCTG	construction of *Thipk1* silencing vector
F-P4803-500 bp	CGTGAGAGGCCAGATGTTGT	GME4803 promoter probe-Lac
R-P4803-500 bp	GGCTGTGAAGGGAAGAGGGA	GME4803 promoter probe-Lac
F-P7109-500 bp	ACTCATCCAAGTTCTGACGG	GME7109 promoter probe-Lac
R-P7109-500 bp	GATCGAGAGTCGGCGGGGAG	GME7109 promoter probe-Lac
F-P3358-500 bp	GCCTCGGAGTTCCTCAACGA	GME3358 promoter probe-LiP
R-P3358-500 bp	TTCGTGATGCCTTAGAGAGG	GME3358 promoter probe-LiP
F-P507-500 bp	TCTTGGCATCCGTCCAAAAC	GME507 promoter probe-MnP
R-P507-500 bp	TGCAGAGGATCTGAGATGCT	GME507 promoter probe-MnP
F-P7667-500 bp	CTACCATGTACGGCCGGGTT	GME7667 promoter probe-VP
R-P7667-500 bp	GATTAAAGTCGTAGAAAGG	GME7667 promoter probe-VP
F-12003-DBD	ggtggatccGGGCGCGAGACGGAGAAG	expression of GME12003 DBD
R-12003-DBD	ggtgctcgagTTACGCTTCGACGCGGGC	expression of GME12003 DBD
F-8051-DBD	attggtggatccGCCAAAGTCAAACG	heterologous expression of GME8051 DBD
R-8051-DBD	gtgctcgagTTAGGCGATGCGCGAGGA	heterologous expression of GME8051 DBD
F-eGFP	ATGGTGAGCAAGGGCGAGGA	eGFP- promoter reporter vector
R-eGFP	TTACTTGTACAGCTCGTCCATG	eGFP- promoter reporter vector
F-P-*Thipk1*	CTTGCGATGGGCAGGGAGAG	*Thipk1* promoter reporter vector
F-P-*Thipk1*	GTCGGTCGTTCAGTCAGGGA	*Thipk1* promoter reporter vector
F-P-*AbgpdII*	GAAGAAGAATTCAGAGGTCC	*AbgpdII* promoter reporter vector
F-P-*AbgpdII*	GGCGATAAGCTTGTTGTGTGTA	*AbgpdII* promoter reporter vector
F-*Poipk1*-q	CAGAGGAAATGACGCAGGAAG	qRT-PCR primer of *P. ostreatus ipk1*
R-*Poipk1*-q	TGAGATCAGTAGCCAGGACAC	qRT-PCR primer of *P. ostreatus ipk1*
F-*Potubulin*-q	AGGCTTTCTTGCATTGGTACACGC	qRT-PCR primer of *P. ostreatus tubulin*
R-*Potubulin*-q	TATTCGCCTTCTTCCTCATCGGCA	qRT-PCR primer of *P. ostreatus tubulin*
F-*Tvipk1*-q	GCTCCGCCTTCATCAACAC	qRT-PCR primer of *T. versicolor ipk1*
R-*Tvipk1*-q	CATCGCTGCTTTGGTTTCCT	qRT-PCR primer of *T. versicolor ipk1*
F-*Tvtubulin*-q	AGAACAGGCTCAACGTCTCG	qRT-PCR primer of *T. versicolor tubulin*
R-*Tvtubulin*-q	ACGGGGAAATGAATACGGGG	qRT-PCR primer of *T. versicolor tubulin*
F-*Scipk1*-q	TTCACCTGCCGAGGATGGA	qRT-PCR primer of *S. commune ipk1*

R-*Scipk1*-q	ACAAGGTCCGTAGCGAGCA	qRT-PCR primer of *S. commune ipk1*
F-*Scactin*-q	TGGTATCCTCACGTTGAAGTA	qRT-PCR primer of *S. commune actin*
R-*Scactin*-q	GTGTGGTGCCAGATC	qRT-PCR primer of *S. commune actin*

Table S2. IPK1 sequences from different species. Fig. 6 Phylogenetic tree of IPK1 original sequence.

>translation-elongation-factor-[Euglena-longa]_out group<br>MSRQKFERIKPHINIGTIGHVDHGKTTLTAAITMALSVTGNTKSKKYEEIDSSPEEKARGITINTAHVEYETKNRHYAHVDCPGHADYI<br>KNMITGAAQMDGAILVISATDGPMPQTKEHILLAKQVGVPNLVVFLNKEDQIDDNELLELIELEIRETLNNYEFPGDEIPIITGSALLAIE<br>ALNKNPKIIKGENKWVDKILDLMDKIDSYIPTPIRDTDKDFLLAIEDVLSITGRGTVATGRIERGKIKVGETVELIGLKNIKSTTITGLEMF<br>QKSLDEAIAGDNVGVLLRGIQKNEVERGMVIAKPGTIQPHIKFNSQVYILTKEEGGRHTPFFEGYKPQFYVRTTDVTGKIESFKSDDGT<br>TVQMVMPGDKIKMIVELVQPIAIEKGMRFAIREGGKTVGAGVIINIID
>Trametes-hirsuta<br>MENDAPIIGDTSPQDWKYISEGGSTIVFSYVGPPHPSFDGTALRLRKGPTPAHEDPEKYEKPSLAPAEPSQEPEASAAGGADDEEPDDPT<br>VVFQRTVIERLVPTAHLPRLQPVRVARAWLAQLAARTEALRPAERRARDAIDLGRRKAVLATDLVGGEGWAVEIKPKWGFLPQPAY<br>LSPETRAIKTRTCRFCMHAQLKKTEGEDVALGYCPLDLYSGDAGRVERALRELWDVWVGSGGAVNNLRVFVQGKMVKPSADPASL<br>APLTAQLLPPTSTTSPDSEPPAATRPPDTLRDAFIATLLPLLLDTPVLRTLSTLQRTLDALDIEGLAALWARVHPAHALGTGPGPGAADP<br>DIAAWARFVDEHIDRLAQAAEDAKGAGEGEGQGENGSEVPATEDELRYRVAAYLLGASFKDCSLILRMRPHAQGTVTVIDLDVKSA<br>DRLGKWAALDREIVDAYRGVSNARRCVDAHYGAVPLP
>Homo+sapiens<br>MEEGKMDENEWGYHGEGNKSLVVAHAQRCVVLRFLKFPPNRKKTSEEIFQHLQNIVDFGKNVMKEFLGENYVHYGEVVQLPLEFV<br>KQLCLKIQSERPESRCDKDLDTLSGYAMCLPNLTRLQTYRFAEHRPILCVEIKPKCGFIPFSSDVTHEMKHKVCRYCMHQHLKVATGK<br>WKQISKYCPLDLYSGNKQRMHFALKSLLQEAQNNLKIFKNGELIYGCKDARSPVADWSELAHHLKPFFFPSNGLASGPHCTRAVIREL<br>VHVITRVLLSGSDKGRAGTLSPGLGPQGPRVCEASPFSRSLRCQGKNTPERSGLPKGCLLYKTLQVQMLDLLDIEGLYPLYNRVERYL<br>EEFPEERKTLQIDGPYDEAFYQKLLDLSTEDDGTVAFALTKVQQYRVAMTAKDCSIMIALSPCLQDASSDQRPVVPSSRSRFAFSVSVL<br>DLDLKPYESIPHQYKLDGKIVNYYSKTVRAKDNAVMSTRFKESEDCTLVLHKV
>Arabidopsis-thaliana<br>MEMILEEKDASDWIYRGEGGANLVLAYAGSSPLFVGKVIRIQKARRNDKAIKNSNGVVSVLTSDEQHLWRENNELISSPNKEVLEQRY<br>VQNVIIPLLGPKHVDAGVRVSVSKEFLECVDKKVTKQRPLWRVNAANVDTSHDSALILNDHSLFSQGITSGGDCISVEIKPKCGFLPTS<br>RFIGKENILKTSVSRFKMHQLLKLEYIEISEESEYDPLDLFSGSKERVLEAIKALYSTPQNNFRVFLNGSLILGGSGESTGRTSPEIGYAFE<br>DALKGFIQSEDGHRTECFLQLVSDAVYGSGVLDRLLEIQKLDKLDIEGAIHCYYDIINQPCPICKEGRPLEAELSLHALPLDESLKIVKEY<br>LIAATAKDCSIMISFQSRNAWDSEPSGDYVSLKPTNQTFDYKVHFIDLSLKPLKRMESYYKLDKKIISFYNRKQKAENTAEQIGNSKPS<br>HS
>Cerioporus-squamosus<br>MTSETPHIADTSPHDWKYISEGGSTIVFSYSGPAHPHFDGTALRLRKGPVPAHEEPEKYTQPQLAETLDLDEDAGVAEEKLEEPDDPTI<br>VFQRAVIERLVPKEHLPRLDAVHVERKWLQELADLTEPHRPLERRAKDRIDTSKRKAVLATDLVGGQGWAVEIKPKWGFLPSPKHLS<br>PETRDIKTRTCRFCMHAHLKSTQGEDVSLGYCPLDLFSGDAERVTRALHTLWDVWIGSGGAVNNLRVFVQGKMLKPTADVMTLAPL<br>AEQLFPAASPEDPPPPAHTLTETRDAFTAVLLPLLLHTPVLRTLATLQRTLDALDVEGLSALWAALRPDDAHKLGKGEADPDMDAWA<br>RFVDEYLARQAGDGESADALQTEEELRYRLAAYLLSASFKDCSIILRMRPPSEGGEHAGTVTVIDLDVKSIDRLSKWAKLDREIVDTY<br>RGIAPRDCVDARATSLA
>Lentinus-tigrinus<br>MTTETPHIVDTSPHEWKYISEGGSTIVFSYSGPAHPQFDGTALRLRKGPVPAHEDPDKYSQPQLESEVEEPDDPTIVFQRTVIERLVPKE<br>HLPRLDAVRVERTWLQELADLTEPHRPLDRRAKDRIDTGRRKAVLATDLVGGQGWAVEIKPKWGFLPSPKHLSSETREIKTRTCRFC<br>MHAHLKSTQGEDVSLGYCPLDLFSGDAGRVARALHTLWDVWIGSGGAVNNLRVFVGGKMLKPTAEVRAFSTLACMHVMSLAPLAE<br>QLFPAASPENTPPPAHTLNEARDAFTAALLPLLLHTPVLRTLATLQRTLDALDVEGLAALWAGLRPDDAHKLGKGETDPDMDAWTRF<br>VDEYLVRQAGDGESGDAVPTEDELRYRLAAYLLSASFKDCSIILRMRPRSEGTEPAGTVTVIDLDVKSIGRLSKWEKLDREIVDAYRGI<br>APRDCVDSSAIS
>Trametes-versicolor<br>MQTDALDIADTSPQDWKYISEGGSTIVFSYVGPPNTLFDRTALRLRKGPVVAHSDPDKHQEPQLAEPSTLEDRQTSKNAPADPDEPDD<br>PTIVFQRTVIEQLMPIEHLPRLQSVHVARAWLEELAERTEPLRPPQRRAKDTIDRGRRKAVLATDLVGGAGCAVEIKPKWGFLPKPTYL<br>SEETRAIKTRTCRFCMHAHMKSTQGEDVALGYCPLDLYSGDATRVEKALRALWDVWIGTGGKVNNLKVFVEGKIIKPRADLTSLAPL<br>TAQLFPTSANPDPAYPLDALRSAFINTLLPLLLHTPVLHTLSTLQRTLDALDIEGLAALWARMYPTIPLGTGPAAHDPDMAEWARFVSA<br>RAARGNQSSDVVPETQEELHYRVAAYLLGASFKDCSIILRMPPRAAADAGVGEKGTVTVIDLDVKGVDRLPKWAALDREIVDAYRG<br>VVPRECVDAHYGGGGGAVPLA
>Polyporus-brumalis<br>MTTETPHIADTSPHDWKYISEGGSTIVFSYSGPSHPHFDGTALRLRKGPVPAHEEPEKYAQPPLAHTLDGGEDVAEDELEEPDDPTIVFQ<br>HTVIERLVPKKHLPRLDAVHVERRWLQELAELTEPHRPLERRARDRIDTSKRKAVLATDLVGGQGWAVEIKPKWGFLPSPKHLSPETR<br>EIKTRTCRFCMHSHLKSTQGEDVSLGYCPLDLFSGDPERVTRALHTLWDVWIGSGGTVNNLRVFVKGKMLKPTADVRNPPPPVHTLT<br>ELRDAFTAAILPLLLHTPVLRTLATLQRTLDALDVEGLAALWAALRPDDAHKLGTGEADPDMDAWTRFVDEYLARQAGAGESADAL<br>PTEDELRYRLAAYLLSASFKDCSVILRMPPRSEGAEHTGTVTVIDLDVKSIDRLSKWAKLDNEIVNAYRGIVPRDCVDAKGAS
>Dichomitus-squalens<br>MADTPQIADTSPHDWKYISEGGSTIVFSYTGPLHPQFDGTALRLRKGPVSGREHTDTEQYQQPQLAETHDQGEEPDDPTIVFQSAVIER<br>LVPRQFLPRLDAVRVERAWLQQLAHLTEAQRPKERRAKDRIDTGRRKAVLATDLVGGDGWAVEIKPKWGFLPARTHLSDETREIKTR<br>TCRFCMHAHLKSTQGEDVSLGYCPLDLFARDKARVTRALHALWSVWLGSGAAVNNLRVFVQGRKLTPAADVPSLAPLAAQLFPEAS<br>HEGGPAHTPDEVRDAFTSALLPLLLDTPVLRTLSTLQRTLDALDVEGLTALWARLRPADAPELGEGEADPDMAEWTRFVDTYLSRHP<br>PRPESASASEAQTQTRTRAGPPAEADEDELRYQLQAYLLSASFKDCSVILRMKPGQAVGQGTITVIDLDVKGIDRLAKWAKLDREIVD<br>AYRGIPPRECVDAWAISALG
>Daedaleopsis-nitida

MSADPHIGDTSPHDWKYVSEGGSTIVFSYSGPPHPDFDGTALRLRKGPVPAHEEPDKYDTPELVEPDDPTIVFQHAVIERLVPKQYLPH<br>LYAVHVERRWLQELRALVEPHRPQERREKDRIAIERTKAVLATDLVGGLGWAVEIKPKWGFLPAPTHLSPETRAVKTRTCRFCMHAH<br>LKRAHGEDASLGYCPLDLFSADEARVTRALHALWAAWLGSGGAVNNLRVFIEGKMLKPAAEGDPLPSLAPLAAQLFPAPDPSDPPSD<br>PFADPVPPVDTPALREVFTAVLLPLLLGTPVLRTLSTLQRTLDALDVEGLAQLWAALRPAGRALGKGEEDPDTDEWTRFVDAYQARQ<br>QAGEPVGAELAAEAQREEELRYRMAAYLLSASFKDCSIILRMRPGQRGAVTVIDLDVKSIDRLSKWAKLDKEIVDAYKDMPPRNCVD<br>SWASA
>Ganoderma-leucocontextum<br>MADTPHIADTSPQHWKYISEGGSSIVFSYSGPPHPAFDGTALRLRKAPVPERENEASGQYRQPQFAEGEEDEPDDPTIVFQHRVIERLVP<br>KQFLPRLDAVRLDSAWLQELSTLTEVHRPSQRRAKDSIDRGRRKAVLATDLVGGSGWAVEIKPKWGFLPAPAHLSEETREIKTRTCRF<br>CMHAHLKSAHGESVALGYCPLDLFSRDRQRVTRAVHSLWDVWIASKAAVNNLRVFVEGQKLAPTADLFPNLQTLMLDEIRDAFTSA<br>LLPLLLDTPVLHTLSTLQRTLDALDVEGLSALWARLRPEHASRLGKGELDPDIADWTRFVDTYLARHASERDTPETDTETEDELRYRM<br>HAYLLSASFKDCSLILRMRSEQAGTITVIDLDVKGIDRLAKWAKLDREIVDAYRGVVQGDCVDGWASV
>Earliella-scabrosa<br>MSNEPHVGDTSPHDWKYVSEGGSTIVFSYAGPPHPDFDGTALRLRKGPVPDHEEPEKYDLPPLVEPDDPTIVFQKTVIERLVSSEFLPHL<br>DAVHVDHNWLHELSTLVEPHRPMERRMRDRIDVSKTKAVLATDLVGGKGWAVEIKPKWGFLPAPTHLSPETREIKTRTCRFCMHAH<br>LKSTQGEDASLGYCPLDLFSGEPARVTRALHALWDVWLATGATVNNLRIFVEGRMLKPTADDATSLTPLAIQLFPRQSPAPTPPTPTA<br>TEVRDAFVGALLPILLRTPVLRTLATLQRTLDALDVEGLTALWAARRPEGLKLGEDEEDPDMDEWTAVIDAYLTRTSSSNEEGTAELE<br>QTDAELRYRMAAYLLSASFKDCSVIVRMRPREGADEEVSGRVFVIDLDVKSIDRLAKWAKLDRQIVDAYRDMPPRDCVDGGR
>Cubamyces-lactineus<br>MSDAPRIEHTSPRDWKYISEGGSTIVFSYTGPPHPQFDGTALRLRKRPISAAVQTQGVPHGYDTQDNGEKQADRNAVEEEQADEPDDP<br>TIIFQRTVIERLVPRAHLPRLDAVHVARAWLAELAALTEPLRPPERRAVDAIDTGRRKAVLATDLVGGGGWAVEIKPKWGFLPAPTHL<br>SPATRPIKTRTCRFCMHTHLKRTAQSKGAHDDTGGGEIQSYCPLDLFADDPARVRQALHALWDTWVASGGTVNNLRVFVRGKMLRP<br>NAADPTSLTPLAEQLFPGSQVSSSATPPATPDALRDAFTSAILPLLQHTPVLRTLARLQRTLDALDVEGLAALWARLRGADVPLGHGEP<br>DPGMGEWAAFVGAYLARERREGEGEGEESGESEAELRYRILAYLLSASFKDCSIILRLPPLSSNAPKGDNAEGTVTVIDLDVKSIGRLA<br>KWAALDAEIVDAYRGVAHPRACVDAHA
>Lenzites-betulinus<br>MSEDLLRIAETAPEHWKYISEGGSTIVFSYTGPPDARFSGTALRLRKAPVSAHSDIFGAHEDTNGNSNTQTAAEQEEEDEPDDPTVIFQR<br>AVIERLVPAAHLPQLRSVRVERAWLARLAAGTELLRPAERRARDAIDTGRRKAVLATDLVGGEGWAVEIKPKWGFLPAPTHLSPRTR<br>ATKTHTCRFCMHAHLKHAHGEPVARAYCPLDLFAGARAPERVRGALCALWDAWVGSGGGVNNLRVFIGGRMVRPGGEPTALATSL<br>EPLAALLLPPRTPTPTPTPTSTPTSISAPTTDFDTNTLRDAFIDTLTPILLASPILPTLSRLQRTLDALDIEGLAALWARVHPLPSPLGEGMPDP<br>DVEEWARFVEEHDARTSAAAGGQSMAEPPATEEELRYRALAHLLGASFKDCSLMIRLPPSSSDAAAQEEVEKKATATVTVIDLDVKG<br>VDRLAKWAALDREIVDAYRAATAGAGEAEAGEGPRRCVDAWYGAEPLP
>Pilatotrama-ljubarskyi<br>MISQLRVHETSPSHWKYISEGGSTIVFSYTGPSNPSYNGTALRLRKAPLPANEDPDRYQKPTLAREPTEKGDEEPSGKQQEGISRSEAEE<br>RYEDSKNDSKHPDDPTVVFQRAVISRLVPPEYLPRLESVHVEKQWLEQLAQLVEPLRPLERRAKDRIDTARRKAVLATDLVGGEGWS<br>VEIKPKWGFLPSPTYLSPETRDIKLRTCRFCMHAHLKSTQGEDVALGYCPLDLYSGDEDRMQKALGALWDVWVGSGGAVNNLHVFV<br>HGKMLKPTVDIRTAFIRTVQPILRSPVLSRLSHLQRRLDALDIEGLAALWAHVRGPGVPFGAELADPDISEWERFVEAYAARERDIGED<br>ADAKRGDLSTSTLEERAPPTEAELRFRVIAYLLAAAFKDCSVIVRLRPQLTEGGVVRQDATVTMIDLDVKGVDRLRKWAKLDREIVE<br>AYRGVEPRNCVDSH
>Cubamyces-menziesii<br>MRYSSPCNSPYLSTLNSTNTIPTRLRDANMESSMSHSDTPRIEHTSPRDWKYISEGGSTIVFSYAGPSHPQFDGTALRLRKRPVSAAAPT<br>QGVPHGYETQQSSENQADGDTSEKAEKEEEADEPDDPTIVFQRTVIERLVPRAHLPRLDAVHVARAWLAELAALTEPLRPPERRAAD<br>AIDTDRRKAVLATNLVGGEGWAVEIKPKWGFLPAPTLLSPATRPIKTRTCRFCMHAHLRRTTQSEGASNDNDGEDSYCPLDLFADDP<br>ARARRALHALWGAWIASGGTVNNLRVFVRGKMVKPNAADPASLAPLAEQLFHGPQVPAPSEDRASTTAADALRDAFTSAVQPLLQH<br>TPVLRTLARLQRMLDALDVEGLAALWARRRGADVPLGHGEPDPDVAEWAAFVDAYLARRRTEEEGEEEGEEGGEDSEAELRYRILA<br>YLLSASFKDCSIILRLPPRSQSQPQPEGAPAGTVTVIDLDVKRIGRLAKWAALDAEIVDAYRGVAQPRVCVDAGHGVRAGD
>Fomes-fomentarius<br>MSSSESVADPHLSDTHPHDWKYVAEGGSTIVFSYAGPPHVHFDGTALRLRKGPVPALEESDNHDELAPPPFDEPDDPTIVFQRTVVERL<br>VPSEFLPRLDAVRVESAWLRELRGLVEPLRPLERRQRDRIDVGKTKAVLATDLVGGKGWAVEIKPKWGFLPAPTHLSDDTRAIKTRTC<br>RFCMHTHLKASQGASDVSLGYCPLDLFSGDRARVTRALHTLWAIWLDTGAAVNNLRVFVGGRQLKPVAADATASLAPLADQLFPAE<br>SDQDSPAHTPTELRDAFTHALLPLLLGTPVLRTLSTLQRTLDALDVEGLAALWAALRPAGHKLGQGEAEPDMAEWTAFVDVYLARY<br>GPPLPSTSDPNPNSSERGETTRTEEELRFQMAAYLLSASFKDCSVILRMPPPPPRTVASSGDEEGVRGRVTVIDLDVKSIDRLSKWAEL<br>DREIVDAYRRDVAEPRLCVDAAVGRGGLI
>Abortiporus-biennis<br>MDTISLPSIQNTSPADWQYISEGGSSIVFSYSGPVNPQFDGTALRLRKVSNDSPVSPDQSQSAAEGELEEPDDPTIVFQREIIQRLVPLTYL<br>PRLEAVKVEREWVAQLAKLCEEKRPLERRAKDRIDLNRKKAVLATDLVGGKGWAVEIKPKWGFLPSPTHLSPATVATKTTTCRFCM<br>HSHLKSTDGENVSLGYCPLDLFSGDTDRVKKALNTLWDVWIGSSGSVNNLRIFVEGQMLRPSPQSLRPLATQIIPSDARHPDLITIRDRF<br>VDSVLPLLLDTPVLRILSMHQRNLDTLDIEGLSTLWKNAHSPLGPSEGIKIPSLGSGLTQPTIQELSSFVDIYLQKHAAMDHEHPDTANL<br>QYYCLAYLLSASFKDCSIIMRIPPVSFDGKRREGTITVIDLDVKGVDRLSKWEKLDREIVASYVGVGDPAVCVDGQKGSTRL
>Grifola-frondosa<br>MAQTPHIAGTSPTDWKYISEGGSTIVFSYTGPPHPQFDGTALRLRKNVLASELEPMEDEENEKEQDDPTIVFQRAVIERLVPKEYLPRLE<br>AVRVQSAWLAELAAICEERRPIERRGKDRIDVHKRKAVLAADLVGGKGWAVEIKPKWGFLPSSLHLSPRTLPIKTRTCRFCMHAHLK<br>TTQGEDVSLGYCPLDLFSGEEARVTRALHALWDVWIGSSGGVNNLRVFVDGQMLRPTVNPSSLSPLAHKLLSSLPVSPSMPSPAPTLN<br>ELRECLTSALLPLLLEVPVVPKLAVLQRRLDALDIEGLTALWKRVRPTAGKLGAGLEELTPEDWVRFVDAYLERSENPDSDALPETEE<br>ELWYRCAAYLLSATFKDCSIILRMPPPTEDVPTSNGTITVIDLDVKSISRLAKWAELDSEIVDAYAGIRPKGLVRC
>Steccherinum-ochraceum

MPSNITATSSSDWKYISEGGSSIVFSYIGSADEFIGTALRLRKIENKLPYTEYADGKEELDDPTILFQHRIIEKLVPSAYLPRLDSVQTDRE<br>WLELLAKQSEEKRPAERRAKDRIDAGRRKAVLATDLVGGLGWAVEIKPKWGFLPSPQHLSKDTAQLKTRSCRFCMHSHLKTTESED<br>VALGYCPLDLYSGDKERVTKALHTLWDIWIGSSGKVNNLRVFVEGHMLKPSMSPSSLQPLAAQILPQNPDDLPDLNTLRDAFTSTILPL<br>LLDTPVLRLLSTHQRQFDALDVEGLSALWKHANQPLGSPDIPPPVLGEGLAQPTLEELEAFVEIYLAKHTTMDHDHPDTANLKYYCLA<br>YLLSASFKDCSVILRMVPVKDGEEPWRKSVTVIDLDVKSVDRLHKWEQLDQKIVEAYIGLPEPTHCIDQTRVEV
>Panus-rudis<br>MSVSVTETSPKDWKYISEGGSSIVFSYVGPPHPSFNGTALRLRKVNHEPTESEILLLKDVEEPDDPTIIFQHRIIERLVPKEYLPVLESVKL<br>HRPWLEDLAMLVEEHRPKERRQADRIDITKKKAVLATDLVGGKGLAVEIKPKWSFLPSSAHLSPNSVSIKTRTCRFCMHSHLKAGEGE<br>DVSLGYCPLDLYSGEEERVKKALVALWDVWIGSSGSVNNLKVFVDGHILRPSTLPSSIAPLAQQILSSSETEPTLDRVRDQFVAELLPLL<br>LGTPVLETLSTHQRKLDILDIEGLVALWQKAYSSPEYAQIPPYGTGLTQPTLTELEQFVTLYQTKHDTMDHTHPDPANLRYYIIAHLLS<br>ATFKDCSIILRIHPGEGSDLPTKRIAVIDLDVKSVDRLGKWEKLDRKIVENYATVRDPVICVEPKAAQS
>Schizophyllum-commune<br>MAAPLTSDKPPSAITCKADVTVTSPSDWKYVSEGGATIVFSYAGPPNPQFDGTVLRLRKCPAPMLHRASSIQDAVIQEEPAPMASQDST<br>SPAIITAEELEEPDDPMIEYQARCMERLIPKVHLPRMESVRVERSWLERLRDLKDAERPPERRAKDQIDVTRRKGVLATDLVGGDTIAV<br>EIKPKWAFLPSPKHLSEATRGIKTRTCRFCMHAHMKRMQGEKVSNGYCPLDLFSGNPARVRHAVLTLWDAWVESNATVNNLKIFVH<br>GKTIHPSEQETIFAPGAHGDAREAFADAILPLLTETPVLKTLSKLQRSLDVLDIEGLSKLWQYTEMMAPAYRTQFAQFFALPEDEEDYSI<br>SMPGSWPHPQPSTPLSPLTPLSPGMCTPSMPSDFRRRPTDPPTSPLGVSSQYLQEPEPTIDDWSSFLDTYLSPFIQIDHAAPVPEDLRFYLL<br>AYLLSATFKDCSIIVRMDLLGGEKQPGPVDPSRVTVIDLDPKSMKRLRKWEKLDQEIVEAYANVKDFRTCVDEGV
>Antrodiella-citrinella<br>MSIIITDTSPTDWKYVSEGGSSIVFSYTGTASNFVGTALRLRKVDNILPSTENAEEPDDPTILFQHRIIEKLVPVNYLPRLQTVETGRAWL<br>EQLARVSEEKRSAERRAKDRIDVGKRKSVLATDLVGGPGFAVEIKPKWGFLPSPRHLSPTSAALKTQTCRFCMHSHLKTTESEDVALG<br>YCPLDLYSGDKDRVIKALHTLWDIWIGSSGGVNNLKVFVEGQVVKPTTLPVSIRPLATQILPHDLEVPPSLDTLRDAFTSLILPLLLDTP<br>VLRILSTHQRQLDTLDIEGLSTLWKQANQPLGSPNLQPPVLGEGLAQPSIAELEAFVEIYLAKHKSMDHDHPDTANLKYYCLAYFLSAS<br>FKDCSIMLRLSPTENGWSKTITVIDLDVKSVERLHKWEQLDRKIIQNYIELAEPSHCVDQALVDSV
>Antrodia-cinnamomea<br>MSEIASVTKTLPEHWRYIAEGGATIVFSYAGPPDARFDHTVLRLRKVTGHDLFPRSEKSVDAEIIFQRAVIQRLLPVRYLPRLESTSIDKE<br>WLVKLASLCEEWRPVERRNKDQIDVDRKKAVLATNLVGGNGWTVEIKPKWGFLPRPAYLSLSSRSIKTRTCRFCMHAHLKVTKGED<br>VAVGYCPLDLFSGVESRVRRALRALWDAWIGSSGSINNMRVFVKGRAINPMSDLSSLRLLARHLIPSSFDALSPAPTDSNADLCEQFTS<br>ALLPLLLQTPVLRMLSTLQRTLDPLDIEGLADLWARAHTHSLSTVSSELGIGAMEPTSEEWSNMVDTYLDRLKANSSETKPDTHELRY<br>YCLAYLLSATFKDCSIMLKVPSYSEQCGEADDRLGEVNGSITVIDLDIKSISRLRKWMELDKEIVTSYTGVEPRWCIDQLAAMEVES
>Trametopsis-cervina<br>MTVDVTSTSPTDWRYISEGGSSIVFAYIGPSSPHFTGTVIRLRKAAHKDASSSAHHQNEEHEEPDDPAIIFQNRVIERLVSQEFLPRLLSV<br>KVNKQWLQQLRDLSEAHRPEERRAKDGIALERTKAVLADDLVSGEGFVVEIKPKWGFLPSPTHLSPRSKSVKTTTCRFDMHAHVKST<br>QGEDVALGYCPLDLYSGDEARITKAIHALWDAWIGSSGSVNNMRVFCNGLMLKPSSLPSSLAPLAAQIMSPRHVEPSLIDLRDNFTTTL<br>RSVLVQTPLLQDLSYLQRSLDALDIEGLSALWAEFHAITGVSPTPPLGSGLTEPTLSDWDKFLDVYLNKRLTMDHDHPDVANLQYYCL<br>AYLLSASFKDCSIIIQVHPSGKSSIALIDLDVKPIDRLHKWAILDADIVKAYEKLSQPTICVDQLAE
>Spongipellis-delectans<br>MSANVTSTSPSDWKYVSEGGSSIVFSYVGAPSPDFDGTALRLRKVAHYPISPETIVSEDIEEQEDPTILFQCQVIQRLIPSEYLPRLESVDV<br>EGPWLEELARLAEEHRPLERRAKDKIDVRRRKAVLANDLVGGSGWAVEIKPKWGFLPNPTHLSPSTKVTKSQTCRFCMHSHLKTTEG<br>EDASLGYCPLDLYSGEEKRVRKALDVLWDAWVGSSGGVNNFKIFVEGHMLRPTTFPSSLGPIAHQVFSANAAEPSLDTLRDQVISPLL<br>PLLLETPVLRILSTHQRNLDALDIEGLSSLWSETHKPAGSLEAAPVPPLGKGLSQPTIEELENFVDVYLEKHASMDHNHPDTADLRYYCI<br>AYLLSATFKDCSIILRLHPAVGNTPARSSITVIDLDVKSIDRLPTWEKLDQKVVKAYAGVSQPTRCYDRWAPARL
>Hermanssonia-centrifuga<br>MSPDATTTSPSDWRYIAEGGSSIVFFYNGPPNPDFDATALRLRKTANDVFPVIDESVEEEEPDDPTILFQHQVIQRLMPAEYLPRLDAVR<br>VGKEWLEELKKLNEEIRPLERREKDGIDVRKRKAVLATDLVGGTNGFTVEIKPKWGFLPSPTHLSIATRPVKTNTCRFCMHSHLKSSE<br>GEEVSLGYCPLDLYSGQEDRIKKALHALWDVWIGSSGSVNNLRVFVDGQIVSPSLLPSSLAPLAKKLPLPDPSSPPSLTGLRDAFTDAIL<br>PLLMDIPVLRVLSTLQRNLDALDVEGLSVLWSQAYASVNAQAVVDGGAGGSLPVPPLGTGLAEPTLEDWKAFLDIYLTKHANMDHD<br>HPDAANIKYYCMAYLLSTSFKDCSIILRLPRDGANTVTVIDLDVKPVDRMSKWENLDKKIVQSYRSLSEPTHCVDQWASQTS
>Sparassis-crispa<br>MSCLFRITETDPKDWKYVSEGGSTVVFSYAGPANPQFDRTVLRLQKVGISDSIARSSESPAEDLVDPGIIFHHTVISKLVPAQYLPHLEP<br>VLVGRGWLLHLAQLCQAQRPQDRRVKDCVDVRCRKAVLATDFVGGPGWAVEIKPKWGFLPSPTYLSPSTRSIKTRTCRFCMHAHLK<br>YTRGDIVACGYCPLDLYSGEQTRVRRALHALWNAWIGSSGRINNLRVFVGGKLLEPTFEPSSLRELALEFGSVSLSDNFVAINQLRDQF<br>TAILLPIILETPVLHTLSTLQRTLDELDIEGLAALWTRARANGVDSPPPALGDGISQPTIDEWTRFISDYTSKIHTPGQFGKEPQVHELRY<br>YCLAYLLSTTFKDCSIILRMRPLKEGVHHSCDNFDSSVTVIDLDVKSISRMQKWAKQDREIVETYAKTERHECQDRYRW
>Cristinia-sonorae<br>MPLNVASTPPADWKYISEGGSSIVFSYIGTSADFIGTALRLRKIDNRLPYTEYADGVEESDDPSILFQHRIIEKLVEPQYLPRLESVETDR<br>AWLEQLAKQTEEKRPADRRATDRIDAGRKKAVLATDLVGGLGWAVEIKPKWGFLPTPKYLSKTTAPVKTQTCRFCMHSHMKTTESE<br>DAALGYCPLDLYSGDEDRVRKALHTLWDIWIGSSGSVNNLKVFVEGGMLKPSTLPSTLRPLAGQILSSEDESPDLDTLRDAFTSALLPV<br>LLKTPVLGLLSKYQREFDVLDIEGLSALWKQANQPLGSPDVPPPVLGAGLKQPSLEEIEAFVEIYLAKHSTMDHDHPDTANLKYYCIA<br>YLLSASFKDCSIIIRLQPTKDEVEPWRKTITVIDLDVKSMDRLHKWEKLDQKIVAEYAVLTDPKRCVDQGLREWALPA
>Phlebia-brevispora<br>MTLNITAVQPEHWKYVSEGGASIVFSYQGPPHPHFDGTVLRLRKIPYNNAPTSATPEVAEEPDDPTIQFQQQIIQRLIPVAHLPRLESVE<br>VQREWLEELQRLADVERPAERRAKDRIDVCRRKAVLATDLVGGNALAVEIKPKWGFLPSTAHLTAATLPIKSMTCRYCMHSYLKAA<br>KGEEVCFDYCPLDLYSGDEIRVRRALNALFDAWVGSSGAINNLRVFAEGTMVKPSLRPSSLGPLAKHFPRSVAEEATLSKLQGDFVAA<br>LIPLLLGTPVLRILSTLQRTLDALDIEGLSALWAAAHQSAAVQVAADPASVAVPPLGAGLTEPTIADWQAFLDIFLTKHAQMDHDHPD<br>SANVKYYCMAYILSASFKDCSIILRIPQDGPATVTVIDLDVKPVEKLSTWAKLDAQIVDEYRGIPEPANCVARL

>Irpex-lacteus<br>MLVDVSATSPADWKYVSEGGSSIVFSYAGPASPYFSGTALRLRKSATAGGDVEVPHLDDTPQDEEPDDPTVVFQQKVTSRLIPAEYLP<br>RLLCVRVTEPWLQQLHDISNEQRPEARRSKDKIAVKKTKAVLADDLVGGEGFVIEIKPKWGFIPSPTHLSPRSKQVKSRTCRFCMHAH<br>LKSTDGEDVALGYCPLDLYSGDKARVTKAIHNLWDAWIGSSGQVNNLRVFINSQKLEPSSLPSSLSPLARHLIEVANTEPSLTDLRDAF<br>TSLLLPALLETPVLQKLSNLQRTLDPLDIEGLAALWSEAHISGNTQLQTALGTDTSQPSSIPPLGKGLAEPTLSDWDAFLDIYLTKHTSM<br>DHDHPDVANLRYYCLAQLLSASFKDCSIMIHLKQPGSKPTIAVIDLDVKPIERLHKWALLDAEIVKAYEGIAEPTQCVDRWAI
>Phlebiopsis-gigantea<br>MFDVTTTSPTHWKYISEGGASIVFSYVGPSNQAFNHTALRLRKAPLETLNESLSEEQQLEDPDDPSIAFQHQVIERLLPEEFLPRLEGVH<br>VNRAWLEGLARIADEHRPPERKARDAVDTRKKKATLATDLVGGKGLAVEIKPKWGFLPSATHLSSETAPVKTTTCRFCMHSHLKATR<br>GAEVSEGYCPLDLFSGDADRIRKALTALWDSWVSTGGSSNNLRVFVEGQMLRPSATPSSIQPLADQLHLSAEENFTLYTVRDAFISRTL<br>PMLLQTSVLRILAVHQSNLDALDIEGLVHLWARAYASRTPGAALTTGQPEGDDAPPAPPLGASMPQPTLDEWSAFLDVYINKHTQMD<br>HDHPDIANLRYYCLAYLLSATFKDCSLIIQAPDQGLGAVTIIDLDVKPIDRLDKWGKLDREIVEAYRQAGRPTQCVAN
>Obba-rivulosa<br>MPELMSVVSTSPQDWRYIAEGGATIVFAYVGPAHPAFDGTVLRLRKSFHNATSAEIVEDVQNSHFEEPDDPSIVFQRAIIARLVTPAHLP<br>RLEPVLVNRPWLECLEILWNGQRPAQRRALDRIDVTRRKAVLAENLVKGDGWAVEIKPKWGFLPTPAYLSDATKTIKTRVCRFCMQS<br>HHAVSEKQVVASGYCPLDLYSGESTRISRALGALWDTWILSSGGVNNLRIFVEGEPLRPISENPDSIRPLAERLSYGSTSAIPDLTNLRQ<br>KFISSLLLVLLDSPILGILSTLQRTLDSLDIEGLSALWTQVHTSIDSDTPRRFGEGAAEPTFEEWSQFIDMYLARTPEADTGPTVQSNTAQ<br>LRYHLLAYLLSATFKDCSVIVRMLPPGPAEQTGDGREWNASAYLIDLDVKSISRLAKWERLDRWIVERYAQTGDTRICVDARAAT
>Cytidiella-melzeri<br>MSLDVTSTSPADWKYVSEGGSTIVFSYTGPVSPCFTGTVIRLRKTSITSDQSSLPIHSDRPELEQPDDPTILFQHRVTQRLIPSEYLPRLVS<br>VSVNEPWLRQLRKMSNEARPAVRREKDGIAVTKTKAVLADDLVGGEGFVVEIKPKWGFLPSATHLSPRSKLVKTTTCRFCMHAHMK<br>STSGEDVSLGYCPLDLYSGDESRVTKAIHYLWDGWVGSSGQINNLRIFVGGSILKPSLLVSFSHFTPSLTDLRDAFTSALLPALMQTPVL<br>QKLSNLQRTLDPLDIEGLTALWSEAHAAADTQMQTSLGTEPAQPLSMRPLGDGQRDPSMSDWDDFLDVYLTKHVAMDHDHPDVAN<br>LRYYCLAYLLSASFKDCSLMIRVKHGRQVNVAVVDLDVKPIDRLHKWALLDAEIVRTYEGIAQPTLCVDRWAM
>Fibroporia-radiculosa<br>MSEQHHISDTSPQHWKYVAEGGVSVAFSYNGPPHPFYDGKVLKLRKVSKHSGLFHPHSEFEDFEDLEGLAVAFDSAILQRLVPREYFP<br>RCEAVQMDSEWLAQLKLLCEAQRPSERRLRDEIDVRRNKAILIEDLVGWDGMTVEIKPKWGFLPSPTHLSQLSCPVKTRTCRFCMHA<br>HMKSTQGNDSALGFCPLDLYSGEELRVRYALSALWDVWLSTSGAINNMRVFVKGVFIKPVSNSAVLAFMLGFKDPASPDQSPPSMAE<br>IRDRFVSALVPSLVKTPVLGLISSLQRTLDSLDIEGLATLWEHSHAESQPAIPFGTNTTQPTVGEWSEFADEYLRRSASDFNKEPLPDELR<br>YHCLSYLLSATFKDCSLMLRLPLSSFQDDQAKVQGSIKVIDLDVKSISRLDKWRRLDRAIVMTYIKVAEPRRCIDQRGSTPKPRYPK
>Wolfiporia-cocos<br>MQIQSDVGAGEVATTIFHVLSQTSPEYWRYVAEGGATCVFSYIGPPHSMLDGTVLRLRKIRKDQILDADRTTVMCDPNEAPGDAQLFF<br>QRNVIQRLVPPEHLPRLEIVHLDQTWLNQLIQLHDEARPPLRRLKDEIDRRSTTAILATDLVRQEGLAVEIKPKWGFLPSATYLSPQTRT<br>VKMRTCRFCMHTHLRSAQGEKAATEYCPLDLYSCDEPRVRRSLHALWEHWVGNDGEINNLRIFVKGKNIVPSQDAEKLHAISGLFDV<br>ASDTSHNASSLDEIRNRFIDTLIPIVLHSPVLSLLSRLQRTLDPLDVEGLAGLWSRAHSELTPEPSLGQGLAEPSEQLWAHFVDEYNTRL<br>ASLEKEYDEPRPQAHELEYYCMAYMLSATFKDCSIIINLAGKDAKSVKVIDLDVKSISRLEKWDKLDREIVSKYAMLDTPKTCVDRW<br>AKGGQAEQCNALDA
>Daedalea-quercina<br>MIDLISRTSVGDWKYVAEGGSTIVFSYDGHPYPVLTGKVLRLRKIALKTSDVSAPAAALPVGNFDDPTIAFQQTVIHRLVPEDFLPQLH<br>VVKVDKEWLRLLAEVVEPSRPEARRVVDRIDLDMETAVLADNLIGGSDLAVEIKPKWGFLPCPDHLSSATRPIKTRTCRFCMHAHLK<br>GAAEEDVPSNFCPLDLYSGDEHRVGLALRALWDAWVRTDGGINNLRVFAKGRILKPSSDVALLGSLLTSTATPNLALDELRAQFVEA<br>LLPTLLATPVFHLLSFRQRTLDPLDIEGLAALWARIYAHDQSSAPLAFGEHAVEPTLEEWASFVDAYLARCQAYAGSHKGGPVAQHTE<br>LRYYCLAYLLSATFKDCSIILRFGSAGQVDERAHKGQQNASPLAAQAEVKIIDLDIKSIKRLTKWEKTDKEIVDAYVRVEAPRTCVDAL<br>AGVHVA
>Gelatoporia-subvermispora<br>MAARVSVASTSPQDWRYIAEGGATMVFGYVGPAHPVFDGTALRLRKTVHGVRDVDPEESSQGQDGEGPDDPSVVFQNAVIARLVSR<br>EYLPRLEHVLVEKTWLEELDRISDERRPPKRRAVDKIDVTRKKAVLADNLIGGDGLVVEIKPKWGFLPSPAYLSPATRDVKTRVCRFC<br>MHSHPAVSEKQDIKGSFCPLDLYSGETNRVVCALGALWDAWLLSSGGINNLRIFVEGEIVRPNPDKLISESTPAGANVADLRRRFVSLT<br>VPAILDSSSLQLLSTLQRTLDPFDIEGLVTLWTRIHTLTDDNTTRQFGSGVPEPTIEEWSRFIDSYLAKSHLPSANELAKAAPDELRYHTL<br>AYLLSATFKDCSLMIRLPCATALATHNHIPDASIYLIDLDVKSISRLAKWKQLDRYIVESYMQTGDTRRCVDARKGL
>Laetiporus-sulphureus<br>MADLCTQTLPEHWKYVAEGGASMVFSYRGPVHPTYTGRVLRLRKVPLNSVEAELNSTEESQDHAGYSIAFDDAVIRRLIPEQFLPRLE<br>SVLMDSKWLETLSQLTEHERPRERRTKHTVDTRQTRALLAPDLLGNEGIAVEIKPKWGFLPSPAYLSPSTRPIKARTCRFCMHTHMKY<br>AECETVAVTFCPLDLYSGDETRVRRALLALWDSWIASPGEINNLRVFTGGRLVRFTHDSAAFIDIARVLDSYSSSTQLDPRSQPEDLRD<br>RLISSLIPPLLEMPVLRILSSLQRTLDKLDIEGLASLWARAHAAIIPAPALGEGLSEPTLEEWTLFIEEYFSKTSTAVESELHVHELRHYCL<br>AYLLSATFKDCSIMVKIDFPNVNRDNVKTSLHIIDLDVKSISRLAKWQKLDREIVAAYANVEPRNCVDQWAPQ
>Armillaria-nabsnona<br>MAVSISSITATLPSHWKYISEGGATIVFSYVGPPNPDFDGMVLRLRKSGVQTPVLEKQAFGTVAEEEEPDDSSIVFQETCMKRLIPSVHL<br>PKLQSVQVEKAWLEVLSQAHQQDRPLQRTASGGIDTTKNKAVLATDLVGGSGLAVEIKPKWSFLPNKTYLSADNCAIKANICRFCMH<br>SRMKSKEGETVSLGYCPLDLFSNNRGRMKQAVYSLYDNWLTSDGTVNNLKIFIKGTKIGVDQKFSMFSPEAYEESDENVRETFSEVIL<br>TLLVNTPVLRVLNKVQRTLDSLDIEGLSLLWREAHSSANPPKSTNGYTPSGRAIGVGEPNPSLADWSAFIDEYLTQSPDLEDLRYHLLA<br>YLLSATFKDCSIMIRMELLDPRRPQEDVDPSKVTVIDLDPKNMNRLSKWEKLDETIVKAYSAVPLADRKTCLDDWVDEA
>Collybia-nuda<br>MVSLASTVQVTGTLPVHWKYVSEGGATIVFSYRGPPNPDFDGTVLRLRKSILSTVPKITRASELGSQNTETPGEVEEPDDPMIKFQTTC<br>MERLIPPEHLPHLESVHVNRGWLESLIAVHDDERPVDRRLRDQVDLNRTKGVLATDLVGGEWLAVEIKPKWAFLPSPTHLSEITRPTK<br>TQTCRFCMHSQMKAKMGDSVSLGYCPLELFSGDKDRVTKAIHSLWNSWMDSNGTVNNLKIFIRGKVIQPSDTSLMFVESTNQAMDP

RDAFTSALLPMLMNTPVLRILSKLQRSLDCLDIEGLSNLWRAAEKSAPLYRTTFASFFEKAGGTSGEEPPTSPIGVSSSFLASPEPTISDW<br>SDFLDTYLSQTSSQIDHTNPSPKNLRYYLLAYLLSATFKDCSIIIRLDPLQSAVAQSTQVVPERVTVIDLDPKSMDRLRKWEKLDREIVQ<br>AYLGTDENKVCVDGWVE
>Tephrocybe-rancida<br>MTVRASDSLPHHWKYISEGGATIVFSYVGPPNPQFDGNVLRLRKATLSTTPGIQDDTDDPLIEFQQKCMERLIPSEHLPRLESVHLTRD<br>WLDSMITFHDAERPAERRAKDQVDLARRKGVLATDLVGGDWLAIEIKPKWAFLPLPIHLSDATRDVKSQTCRFCMHSAMKSKMGEA<br>VSLGYCPLDLFSGNKDRVQKALYNLWDAWSESNGTVNNLKVFVHGQKILPSQASLMLLNNFNAETSHSAIREAFTESLLPLLLETPVL<br>RTIATLQRELDSLDIEGLSKLWRVVQSAHRQSFRIDDSSPPFPPIGVSSAYLTSTEPTITEWTKFVDVYLSSSAGLDYRNPAPVNLRFYLL<br>AYLLSATFKDCSIIFRLDSLQPGPAKVKPGQVTVIDLDPKSMARLRKWEELDREIINAYIAGEGRKLAVNQTLRSSSSEMSALRFLTKN<br>VRNRQIRTMVLPVTSRRTVSTYNNDVAGLSEDEAEFRNAVVDFAQKEVAPRAAEIDKTNTFPAVSIALLRRYLPLTIDDLKLQDLWEK<br>FGSMGLLGITVASKYNGLSLGYFHHTLAMEALSAASGSVALSYGAHSNLCVNQIHRHGTEEQKAKYLTDLVNGTKVGSLAMSEVGS<br>GSDVVSMRLTAERVQGGWKLNGSKFWITNGPVASTLVVYAKTAPDMGSKGITAFIIEKDFEGFSTSPKLDKFGMRGSDTCELVFDNC<br>EVPDANVLGQINKGAAVLMSGLDLERIVLSGGPLGLMQAAFDEAVEYVHDRKQFGQPVGTFQLMQAKIADMYTKLNASRSYVYAV<br>ARACDRGTVSRRDCAGAILYSTEKAVEVALEGMQCLGGNGYINDYPMGRILRDSRLYTVGAGTQEIRRMLIGREFNEQLKQ
>Neoantrodia-serialis<br>MNSLVSETVTVNWRYIAEGGSAIVFSYVGPVHPLFTNKVLRLRKIAVKPATIDVPAPAPRRGDLDDPVIAFQEVAIRRLVPDEFLPHVQ<br>VVKVDKAWVQLLAEAAEASRPEARRVEDRVDTDRETAVLADNLIGGSGCAVEIKPKWGFLPCPDLFSPTTRSIKTRTCRFCMHARMK<br>GAAEKDVPSDFCPLDLYSGNESRVRVALRALWDAWVRTEGGINNLRIFANGRVLKPASDSLLAPATEVMPDPSLDTLRTQFCDALLPF<br>LLNTPVLRLLSFRQRTLDPLDIEGLSAFWGRIHSDDESDPPLGFSEAEPTPEEWTRFIDKYLVVCDTCVGVKAEPEPNKAELRYHCLAY<br>LLSATFKDCSLMIRMGNRAGTVSLDGGAGDPVDADQQDPHTRAIAPSEVKLIDLDIKSIKRLAKWEKLDREIATAHAQVEAPRTCVDS<br>LAGIRISP
>Psilocybe-cf.-subviscida<br>MADVVHTQPKDWKYVSEGGATIVFSYVGPPNPAFNGTVLRLRKRKVSLGDQSLEIDLGDEPDDPSIVYQTKVMSRLIPPEHLPVLQTV<br>HLERGWLQDIAQFHAASRPEDRIRVDTIDLSRKKGVLATDLVGGDWLAVEIKPKWGFLPVPHHLSAATKSIKTRTCRFCMHTHLRVQ<br>QGHEDVTTYCPLDLFSGDEQRVVKAIDSLWDAWIASKATVNNLKIFARGKFVKPSEASIMLADSETHTAGIDLEALHDAFVTSLKQPL<br>VKTPVLRILSELQRNLDVLDIEGLSELWRLTQTSKPLYHQSFASYMGEASEGHISTPPTSPIGVSSQFVEFPEPNIGDWGEFVDIYTTLDK<br>PVLDHANPSPENLRYYVLAYLLSATFKDCSVIVKMDLLDPTRPQSIDSTPTPKFNVTIIDLDPKSIEKLRGWEKLDREIAHMYQEASDK<br>KMCVDAWEVPRSTVAP
>Pleurotus-ostreatus<br>MAAHQVASVADTLPLEWKYVSEGGATAVFSYVGPSDPQFDGMVLRLRKCPLGQPFRSAEEMTQEEPDDATVEFQQRCMERLVPPIH<br>LPRMVSVKLNEPWLRAFSEAHNIDRRAERRRVDEIDWSRTKGVLATDLIGTDGLAVEIKPKWGFLPSPKHLTTTTSSVKARTCRFCMH<br>VHLKVLQGDTDVSVGYCPLDLFSGNPERIKLALESLWSSWLATDGTVNNWKMFVAGMIVRPQTIHDLKVTLLGHGSDEDDPNVLRD<br>KCVELLMPLLTDTPVLKVLAKLQRTLDILDIEGLSSLWHRTLVSYADPAAGPDTPLSPVGQTSSLFESPDPTIDDWSKFIDQYLMQIGTT<br>TEEEKMNHAQPLTSNLRYYLMAYLLSATFKDCSIIVKPTMPGMHGKDSITIIDLDPKGMDRLRKWERLDAQIALAYGRIDEASRKICV<br>DAGVDAW
>Coprinellus-micaceus<br>MPPSVVDTSPSDWRYVSEGGATIVFSYNGPPNPNFNGTVLRLRKALVPALRTVTREGTVQTRANNEEPDDPTIEYQTKCMSRLIPPEHL<br>PRLETVHLDEPWLERLVAQQNLRRPETRRETDGIDVDRQKGVLATDLVGGDWLAVEIKPKWGFVPSPKHLSAATKDAKTQTCRFCM<br>HNHLRKSKGHIIEMSYCPLDLFSGDEARITKAIHGLWDAWTESGGSVNNLKIFANGKIVHPTEPHRMLARDAKPDTPIREIRDLFTSALL<br>RILTATPVLRILSELQRSLDVLDIEGLSRLWRRTERTAPSYRTTFASFFEHTKGDGVEKPPSTPLGVSSLFLSEPQPDIEDWEEFLDTYLSP<br>FRDGLDNSKPAPENLRYYLLAYLLSATFKDCSLIARLDFLKPGVEQEVKVGPQTVTVIDLDPKSMEKLREWEKLDKEIATFYSTNTEK<br>KTCVDAFAS
>Desarmillaria-ectypa<br>MSVARTPIIMAVSISSVAATLPSHWQYVSEGGATIVFSYVGPPNPSFDGMVLRLPKSGVQMRVLEKQAFGTVAEEEEPDDSSIVFQETC<br>MKRLIPSVHLPKLQSVLVDKAWLEELSRTHEPDRPLQRTVSGGIDTTKSKAVLATDLVGGSGLAVEIKPKWSFLPNKTYLSAANRSIK<br>ANTCRFCMHSRMRSGKGETVSLGYCPLDLFSNNRGRMKQAVYSLYDNWLKSDGNVNNLKIFVKGTKIGMGQRFSMFSPEAYEESDE<br>NVRETFSEAILALLVGTPVLRVLNKVQRTLDSLDIEGLSLLWREAQSPANLPQSTNGYTPPGRAIGVGEPNPSLADWSAFIDEYLTRSPN<br>LEDLRYHLLAYLLSATFKDCSIMIRMDLLDPRRPQEEVDPSKVTVIDLDPKNMARLRRWEKLDEAIVRTYSAVPLTDRKTCLDDWVD<br>EV
>Flagelloscypha-sp.-PMI_526<br>MSSATPSIHPLVEESASVDWKYISEGGATIVFSYIGQPHPDFNGMVLRLRKRDLTGIYSESDKFVLTEPHFNPEGFEEPDDPQIAFQNEIM<br>SRLFPPEHLPVLRTVHVNRGWLESLALLHNNERPPKRRSVDQIDVSRTKAVLATDLLGGTGALGVEIKPKWSFLPNPAFLSDETREIKM<br>NTCRFCMHTHMRHVEEGGVADLPYCPLDLFSGTKERVTRAIVALWDTWESTGGKANNLKIFASGKLVSPGDKQLLMTPPGPAELPIP<br>EISAIKASFVDAMVRPLLETPVLERLGRLQRTLDALDVEGLRALWNLAGKTLPMGHGEAQPDIAEWTSFVNDYLTRNKGEVKLPDAG<br>SLDPKDLRYFMLSYLFSATFKDCSIIIRRAGPLDIVDYPAKEAITSTVTVIDLDTKDIERVAKWESLDTKIVQTYAKLETERRRKCIPVYQ<br>QVA
>Hypholoma-sublateritium<br>MANVLQTAPQDWKYVSEGGATIVFSYVGPSQPALDGMVLRLRKTALSAANLVHEVREWDFDSADPEDEPDDPSIEYQVKCMQRLIP<br>AEHLPRLQTVTIRRSWLEAFSVFHNPARPEGRRIVDEVDLTKKKGVLATDLVGGHWLAVEIKPKWAFLASPTYLSDETKGVKTQTCR<br>FCMHTHLRTQQGSQEITEYCPLDLFSGDECRILKAIHSLWNAWVASNATINNLKIFAEGKFLTPSECQLMFADRHAAGLDLKAIGDAF<br>ASTLIQPLLRTPLLRILSDLQRNLDPLDIEGLSKLWKLTESTIAMQTLQGSDSVPPYTPLGGRSPFFQSAEPHISEWIDFLDTYGSTDRPDA<br>ANVKPSPEDLRYFLLAYLLSATFKDCSVVVKLDFLQPYPQSTGTISSDSVTVIDLDPKKMSKLKGWEKLDAEIVRAYALANQKTCIDA<br>WKSPQSVSSSQLTVPNRLS
>Candolleomyces-aberdarensis<br>MASIVDTDPSDWRYVSEGGATIVFSYHGPPNPEFDGTVLRLRKALVPSLKTVVKIKDGEVYKQQALDGEDEPDDPTIEYQTKCMAKLI<br>PPEHLPRLETVKLDQEWLQRLAQLENLKRPEARREKDGIDVNRRKGVLATDLVGGNWLAAEIKPKWAFLPSPSHLSEETNSIKSQTCR

FCMHNHLRKSNGDIIEMNYCPLDLFSGDETRITKAIHGLWDAWTESGGTVNNLKVFAKGKTLRPAETSLMLADGLDASSTTTEQIRDA
FTSALRRTLIKTPVLGILSRLQRTLDVLDIEGLSKLWRTTERAAPLYRTTFESFFAQPDGTSPPPTTPLGVSSLFLSSPEPAIPDWVDFLNT
YLSSFSSQLDHSKPEPKNLRYYLLAYLLSATFKDCSIIVRLDFLRPCIEPKIEVKPNTVTVIDLDPKSLDKLRGWEKLDKEIAGTYSLNTA
RKTCIDAFSSPT

>Cylindrobasidium-torrendii
MSNSPVEISVLSTRPADWRYVSEGGATAVFSYVGPANASFDGMVLRLRKAGVSTPVLEKVLSQEFEFGSAAQGAERVDEPDDASIVF
QEQCMEKLIPPVHLPRLSSVRVDQTWLEEFARFHNASRPLVRSVAGGIDPARTKAVLANDLVGGSGLAVEIKPKWSFLPNLKYLSDST
LPVKSRTCRFCMHSHIRDSNGEAVSIGYCPLDLFSNDPARMKKAVHSLYDNWVKSEGQVNNLKVFVKGKKISFKERFSMLQAGEESD
EELRETFSDAIMRLLVGTPVLQLLNRIQRTLDPLDIEGVWHLWRQAHPTSAHLAQNEANPTLADWTAFIDSYHSRVHFTSEEPKVEDL
RYHLIAYLLSATFKDCSIIVRMPLLDPARTLETVNQENVTIIDLDPKNMDRLKKWHDLDTIIAWEYSTIPEAERKTCSDGWDLKAASTS

>Lyophyllum-atratum
MLARVTDTLPEHWKYVSEGGATIVFSYDGPPSPQFDRTVLRLRKSLVPAVPLRTASAGGTGYEPDDPMIEYQQKCMERLIPLEHLPRL
ESVHVDREWLENLAALRNIERPRDRRDKDQVDLSRTKGVLVTDLVGTNRLAVEIKPKWAFLPSATHLSDGTRDAKTQTCRFCMHSA
MRAKAGETVALGYCPLDLFSGEERRVKQAIYNLWDTWNESNGTVNNLKIFVHGTTIAPAEVSSMLSNNADAETYSGDLREAFASALL
PLLLGTPVLQILSTLQRNLDVLDIEGLSNLWQLAETSAPLYRTTFASFFEGQSTGIDDGPPPSSPIGVSSLFLTSPEPTISDWTNFLDMYLS
QTSTQLDHQNPAPENLRYYLLAYLLSATFKDCSIIVRLDQLRPTGPAQEVKPEQVTVIDLDPKSMARLRKWESLDQEIVREYIAADDRK
VCVDASR

>Mucidula-mucida
MSVSIFSVSSTLPSHWKYISEGGATIVFSYIGPPNAELDGTVLRLRKSGVQTPVLEKSQFGLDVGDEEPDDASIVFQEQCMERLIPPVHLP
RLRSVRVDEAWLTEFARAHNVDRPLERSAVGGIDTSKTKAVLANDLVGGVALAVEIKPKWSFLPNKTHLSEINHPIKSSTCRFCMHSH
MRSGEGESVSLGYCPLDLFSNNPARMRKAVYSLYDNWLKSDGSVNNLKVFVRGKKIGTAERFLMLENAEETDDVMQEAFADAMLP
LLIDTPVLRVLNKIQRTLDALDIEGLSLLWKRAHTVAEVTTYGSPPAVGVGAPNPTLADWASFIDNYLSQPPFNHASPNPENLRYYLLA
YLLSATFKDCSVIIRMDLLDPSRPPTAIDPSKVTIIDLDPKNMNRLQKWEKLDQIIVDAYREVALEDRKTCLDDWVDSNVEA

>Rhodofomes-roseus
MTSHVSQTATEDWRYIAEGGSTIVFSYNGPVHSSLDNKVLRLRKISISVGPVGRPKGLDDPAFAFQQRVILRLVPDESLPHLEVVKVEK
KWLRLLADAAEANRPEVRRAEDRIDWDRETAVLADNLIGGSGLAVEIKPKWGFLPCPDYLSPTTRRIKTRTCRFCMHAYHKDAAEKH
VPSAFCPLDLYSGEEHRVRVALHALWDAWMQTDGGINNLRIFASGRVLRPTTDVCLSRSLLTSTESTTSHSSVDALRIEFINALLPTLM
TTRILRLLSFLQRTLDPLDIEGVAALWARIHGDKDADAPLGLGEGVPEPTLDEWTRFVDAYLATSQTRTNTCAVESDPVADREELRYY
CLAYLLSATFKDCSVVIRAGSHPGPETLDTAADGQEEHQDAHTCRKTTSEVKLIDLDVKSIKRLAKWEKLDREIVTAYAQVETPRTCR
DSLAVMRRFP

>Panaeolus-cyanescens
MCSSKGYPDITHTRPEDWKYVSEGGATIVFSYVGPLNPRFDGTVLRLRKAIVPKAGNKGEITTPSEAPSIPTEEPDDPTIQYQEKCMSRV
IPSDHLPRLETVHLERSWLERLVNQHDTIRPQGRRDKDQVDLSRTKGVLATDLVGGNWIAVEIKPKWGFLPNLHHLSEASKGVKTQT
CRFCMHSAMRHEEGEQVASGYCPLDLFSGEKERIIKAIYALWDAWISSDATINNLKLFQRGSLVKPSNMQSLIAEDYTRDHLEALSIRD
AFAEALVQPLLKTPVLQTLSQLQRDLDALDIEGLSKLWRETESSAPLYRTTFSSFFERREGDAPPNTPIGVTSLFLDAPEPNMSDWIEFL
DLYLSSASRELDHHNPRPENLRYYLLAYLLSATFKDCSVIVRLDFLKPGSSTQGILQSHTATVIDLDPKSMDKLRNWEKLDQRIVNYYT
SLPPSKRKECVDANKTT

>Fomitopsis-betulina
MVTISVSRSSIEDWKYIAEGGSTIVFAYVGPANNLLSNKVLRLRKVKHQTNGTKEDALAPPLNDLDDPAIVFQQAVIRRLVPDCFLPSL
EIVSVDKKWIRLLAEAAEDHRPEARRSVDRIDWDKEAAVLADNLIGGSGLAVEIKWGFLPHPDHLSPVTRPVKTRTCRFCMHAHAKG
AAEKDVPLEFCPLDLYSGDEQRVRRALRALWTGWVRTDGGINNLRIFADGSVLRPSSDAPSLHRFLTAAMVSSVPSLDGLQADFVETL
LPVLLKTPVLALLSFRQRTLDSLDIEGLAALWARIHADSDPFFDAAEPTLEEWTHFVDVYLAGEPDWRQETEEAELRYHCLAYLLSAT
FKDCSVVVHFGPRSERLPVVGDAHIAKQDRGLCAVAPAEVKLIDLDVKCIRRLAKWERMDGDIVSAYARVDDPRTCLDSLSRVCSLA
TSLER

>Pterula-gracilis
MSTQRPHVGSTAASDWKYISEGGATIVFSYVGPSNLLFDDTALRLRKISHDLPLDFSLLEDEEPDDQMIAFQHRCMDRLIPPEHLPHLQ
SVTVSSEWLKAFAQAKDAERPQKRREKDGIDVCRKKGVLATDLVGGRGLVAEIKPKWGFLSSTRHLSEETRPIKSKTCRFCMHSHLK
ASEGATVAQKFCPLDLFSKDPERIAVAVESLWSDWVSSEASINNLKVFLNGHKCDPTSDMSGLATSLNVPGDDAEDIKASFVQAVVSI
LSTTPLLHLLSQLQRNLDALDIEGISSLWAKAHVSSPTGESTVIGLDEEYIPHPNPSIEEWCSFIDLYLSETFQASLDHDNPHIAHLKYYLL
AYLLSATLKDCSIILRINPGEGHAMVIDLDPKGLERLHKWEALDRSIVSHYAALDPNARKTCVDDNR

>Postia-placenta
MTHQPSLSNTSPGDWKYVAEGGASIVFSYTGPADHRFSGTVLRLRKTTHDFVNDSAAESLPTKDIDDLFIPIHQRVTRRLVPEGYLPRL
LSARVEREWLMSLDQLSANQRPLDRLRTDRIDISRHNAILVTDLVGSAEWAVEIKARTCRFCMHTSFKAGQRGDTELHFCPLDLYSGE
EYRVRRALCALWDSWISSCGTINNLKIFVKGEVLKPTGEVQSLQLLVRQLSRTETATFDTSAALSLRDIRERFVASLLPILLETSVLYTLS
NLQRTLDPLDIEGLSTLWTNSYPQFVSSSVFGTSAQEPTLEEWTRFIDEYLSRTTITGRSGHHPDPQPEDLRYYCLAYLLSATFKDCSIMI
SMTPATGEQDAGRRGSVKIVDLDVKRISRLAKWERLDKEIVSAYASSANPRTCVDQQQVMES

>Blastosporella-zonata
MALSVTETFPHQWKYVSEGGATIVFSYIGPPNPRFDGVVLRLRKAALPSVPSLQEGIQDEPDDPLIEFQQKCMARLIPSEHLPRLESVHT
TRDWLESLVILHDPERPPERRAKDQVDVSRRKGVLATDLVGGNWLAVEIKMKSKTGEAVSLGFCPLDLFSGDKDRMLKAIYDLWDA
WSNNNGMANNLKIFVHGSKISPSEANPMLSDNAETSHSELREAFARALLPLLLETPVLHIISTLQRSLDSLDIEGLSKLWRLAESAQGRS
NGMYDAPPSHIGASSAYLDSTEPTITDWTKFVDVYLSSSACLDHSSPLPENLRFYLLAYLLSATFKDCSIIVRLDNLRPTGPAKKVKPEQ
VTIIDLDPKSMARLQKWEKLDREIVEAYIAGSGRKVCVDALR

>Coprinopsis-cinerea
MLDIRETKPTDWKYVSEGGATIVFSYTGPPNPNFDETVLRLRKALVPALRKQKKNKKARKSPWSSPPSTPTTPTSDPDDEPDDPTIEYQ
TKCMSRLIPPELLPRLESVRLDRQWLEDLVELQNLNRPEVRRDKDGIDIDRRKGVLATDLVGGNWLAVEIKPKWAFLPAPMHLSEETK
PIKTQTCRFCMHNHMRKMKGDIIEMCYCPLDLFSEDEDRMRIAIHALWDAWTRSEGTVNNLKVFAHGNLVFPHETKLMAAPDAQDA

NEEQIREAFTDALVRTLRKTPVLGILSKLQRTLDTLDIEGLSRLWRHTEVSAPLYQTTFASFFAHEPNAKVPPSTPLGVSSLFLSSPEPGIP
DWIEFLDSYLSSFSSQLDHSNPSPKDLRYYLLAYLLSATFKDCSIIVRLDFLRPGSEPHIELKPHTVTVIDLDPKSMDKMRGWEKLDKEI
ATVYSRKEDRKTCIDAARI

>Tulosesus-angulatus
MANISNTAPSDWRYVSEGGATIVFSYHGPPNPDFDGTVLRLRKALVPSLRPVVKVKDSEDGEAGNGKVVFHEEPDDPTIEYQIKCMSR
LIPAENLPRLETVLLESEWLERLVELQNLKRPEGRREKDGIDVDRKKGVLATDLVGGNWLAVEIKPKWAFLPSLTHLSDATKATKSQT
CRFCMHNHLRKSKGQIGRMTRAVHGLWDGWHESGGSVNNLKVFAEGKILRPSEANSMLAGGVSTEYSINQIRDAFTTALVRTLVRN
PVLSILSKLQRNLDVLDIEGLSKLWRETETLAPLYRTTFASFFEKPQESDAPPSTPLGVSSLFLSSPEPDIPAWEDFLDSFLSQFSAQLDNS
QPVPQNLRYYLLAYLLSATFKDCSVIARLNFIDPGAEPNIKTGSNAVTVIDLDPKSMDKLRGWEKLDKEIATTYSSNPERKTCIDAFAA

>Leucoagaricus-leucothites
MIMFKLSTPTPTLKGAMDDITQTLASHWKYVSEGGATIVFSYVGPPDPSYDGMVLRLRKAVNESRPEPDVDGENEPDDPIIEYQTRCM
ERLLPRKNLPGLRSVRLERDWLERLAEKHEKERPEVRTKKDHIDFSRKKGVLATDLVGGDWIAVEVKQPRYISPNTLLAEMGLFALSD
PSIGGDKDDQDADLSFFFMRNQKNQGYVNNYCPLDLFSGSADRVRKAIDSLWDAWEQSNGGVNNLKVFASGKVVEPDKVKHLLDG
KFDGISSHEQIEQEFKEALFTTLLESPVLAIISKLQRTLDALDIEGLSKLWRETELQSPLYQSAHKSYFEQPISNERIPTTPLGVPSAYLPTS
EPSISDWVEFLDIFQSTQAAEMDHSQPKTENLRYYLMAHLLSATFKDCSIIVKLDFLKPGNQSGRRVYPVSVIDLDPKSLDRLQRWEQL
DREIVRSYTPLERKICIDG

>Helicostylum-pulchrum
MFHSQVVVSMVPSLNKSDEVDNWVYLGEGNLNLVVAYRGDDPNYKGKVLRVIKNCKNEKLSAQDSILFKKQFSEKVIQPLLGKEYV
VPMFPVHTSVEFLQKLAFNVEPHRPAFRTDRQIVVNSPTSFLLDDLTQIWPNSTAMTFELKPKWGFKPDSSSIDMSNTIKKRTTCRFCM
HSYLRRVPQNNFCPLDLYSIDQVRVRKAVNVLLKENPLKKTLSVSINGKKASLTSEIDQQLFSTFIEYNDDHNTLMQDILERVLLHDPIL
KILKSLQMNLDEIDVEGIYSLFQRNKDYFKLVMDDINIWMDVVNRYQQRLEMENSSINELDEQKQRIYEYILSMTFKDCSLMINVTPT
VEGNNTSKIITLPNGLSFQYDIKVIDTDLKSIDKIPFWFELDQSIVRHAIETKFDKECHQ

>Saccharomyces-cerevisiae
MQVIGRGGANILIDYGDPTWLWRCCIRWPDLLSSNNSYTIKNISYIKDYVEPLLHGLLCPMYLIDVDIEAIRPILSDFILNLDDKVVKVIK
IKNLTNNTSNLILNNHFLKSYCSQNLQTVILELKPKWLYYDTDYCRNCTHNAFKGRGTKYCYNQLLMNPAHLELIFGECNIFPVKFKD
AMHEYLRNDNNIFKILYDLQKKLTKNTTPISDIKSINDVNDEHLLLMTLRDVTCFIEWNSAENALHVNIIDVDLKPKEKWTHWTKTYS
QLTSSQKIYHTSNK

>Kluyveromyces-lactis
MTLLFVGKGNANVCYLLSSEVYRISLRYQKLSRNNAYVRENFQFIDSKIRSLPMLADVVVSMRLEEVFVDKEWTNVLKDENILIDDSH
MQCIVMPLLHAKDSTCEQLDHFNQIYRCSLNDVVTWEFKPKWLYQSSDYCRNCTHNSLKGRDIEYCFLHDPELIIETLFEGRQLPEEFL
DDILQYLQSSDSITQRLYAAQRFVKDDLSTLMTLRDVTCFLTWSRNTRSVKATIIDVDQKPANKLRHWQSTESALASFPGRKKAHFNH
E

>Pluteus-cervinus
MLNATTTSPHHWRYVSEGGASIVFSYRGPPNIGFNGTVLRLRKVPRAPKDGHSKTPIVVEDEPDDPIIEYQQRCMEKLIPIEHLPRLESV
LLDKAWLEEVSDVHDAFRPESRTKHDCIDVTRKKGVLATDLVGTSGIAVELKPKWGFMPSATHLSEATRSIKTQTCRFCMHSRLKSHS
GQQVASGYCPMDLFSGDESRITKAIHDLWDAWYLSEGTVNNLKVFVKGTVVRPSEAARMLDQGYDSEQSIDVLRDIFTAALLPLLKD
TPVLHLLSKFQRTLDPLDIEGLSKLWSSTQATAATRFVNQNRSNSESPNASELAPSSPIGVATEYFEVPEPTVDEWCNFIDTYLVQHPA
MDHINPSPDNLRYYLLAYLLSATFKDCSVMLRLDLLRPGAENVSGEADRVKVIDLDPKSISRLRKWEKTDQEIHLFNADQEPLGCIDQ
HAARNYLV

>Schizopora-paradoxa
MDQGLESTSPSDWKYLAEGGSTVVFTYNGPYNAKFNRCVLRIRKSFEDKPEGATDEDDLAIEFQRRVTSKLLDHEWLPRLESISIEAS
WLQELDSLSRDHRPPERQSSPAGSIDLNLRKAVLSENLIGGHGWAAEIKVIIILRNEDPSQTYIQPKWGFLPNPEHLSAETRDTKLNHCR
FCLHRYQRSLQHQKTSEHYCPLDLFSGETGRIKQALSSLWDSWKSSEGHSNNLRLFSAGQLVDPCNEISLNHLRTSIKSSLPPSSRQNSS
VKESFVEASLSILSRSPVLNALSRLQRSLDPLDIEGLYSLSQSPKSVQSAMHGEPSLEEWETFINNYLDPVFQASLDHRDPDPANLRYYA
LAYLLSASFKDCSIILRFLDGPETPPAISVIDLDVKSIERLKKWKNLDYEIATCFACDGPPDSKCRDAHSL

>Cryptococcus-neoformans
MNDGNHYRPAISPNPSADTQPSDWAYIAEGGAHIVFSYQGRSKTYATRALRVRKPSATTEPLAQAEENDLFGQWRRNILPKLLPRQLL
TTSREVILEDRWYKELLAMVDVVRPDQRKSGIDFAAKGDRRGVLLEDLTSSEDDGAIAVAIEIKPKWGFLPCTEHLHPPESVSIKSHVS
RFRLHQHFRGCSDDPPYDPLDLFSGDKMRMRTALDGLWTMWEISRGKVNNWKVFVGGKEISPDDLQNGLLPIGGDDFVTKITQLTLD
TLQTSFALPLLKNLQQNLDPIDISSLAALFQAEYPNSPPFDPDLIPDVSAVELNGFVDIYISDPQAGQRMDSWSLRERIIAYALSAIFKDCS
LFIRGFLKHAEDGVWRLVSGSDSVKVIDLDLKPIKNIQKWAETDEKVWKYWLETKGAR

>Coemansia-pectinata
MAVPSSVDLPESIPFEPNDWNYKGEGNENIVFSYRGSNTDICGWLLRLSKCDVATSVNGCTAHRLDDTQRKRDHATYSTSVIGPLLGP
EYILPQRLVALKPDFLRRLQVKSEPLRPAHRLHKQIDMSQDVGVLMPNAFRGAPPVSSSGRLRQSVTVELKPKWGFMPSLGSVSPRNS
VKSRVCRYCMHQFTKHASQDVSGFCPLDLYSGDRHRIVRALDSLAQSPQNNLRVFVDDHIAIDRHGKISEECVAQWEALRDCIADIVL
KEPLFSRLKQCQRMLDSLDIEGILPLYKRAVESGSLANVQPSISDWLTAVADFTQRTEIADSNVAEVTDKQAILEFLISTTLKDVSVLITL
PEWPVHKTSLDSVPTEPQYAIAVIDSDPKKLAKIPNYFSKDEKIVKHYLKHCPDLDSQQQCSE

>Gongronella-butleri
MARWNNDDWDYLGEGNAHVVFQYKGNDPRLYGRVLRLTKQGSAPSVAFDLGFVEHVMRPLLGGAYIDAAEKVTLPGEFLHAIQQK
SHAQRSANGKADARTVAPSASLMIDYTRIFTPNDTWTFEIKPKWGFLPSSPKIDVRHRALKQTMCRFCMHQRLRLGNVSPDAPATRYC
PLNLFSGDKIRMETALASSLRAPHKYMHVFYNGKRVDCQKERIARCRQLLLPGTADTRDDGGQVTDTVAQLLAVILRQDPILARIKDL
QQKLDALDIEGIYPLYEKQKARPLGDYNLDEWCQVVETFLARDHAANQDRDKNDAMDEDNENDERQRLHEYVLSMTLKDCSVMV
CVTPVKDRRLPNGRVVDFLGSNFLFEIKVVDVGMKSSTKIPYWYDLDGKIVKHNLGCGTDWHCRGF

>Linnemannia-hyalina
MATILELYRVDYWKYRAEGNANIVLQNTVLRLRKANRLDDPGTGLQGTNFGDGTMTMKDLTMESHFATKVIGLLLGQEFVEQLIAV
ALPPGFLPSLALAMEPSRPESRRQKGIDLAQSVGFLALDHTRFIKPSPGQSDVAIEIKPKWGFLTKSAFLRKDQDIKRRKCRFCMYQYQ

KIKTGQEGTLSKYCPIDLFSGRELLVQDAVDALVDTPQNNLRLFVDGIQKPVSRESMAQCLSAPSTSATTLLHEQQQNRKPQWMESDD<br>PLGYEEDDSGPVRLTDVLTKILVESPLLRRLGRLQQALDSLDVETVHQLYTYLIEHDSAHLPEPTVEEFLETAEAFMDRTDMNAMMTE<br>DQDTFESHNAAGLGFEPEDDLEELPGPLQLHFIREFLLSATLKDCSILITIQRETATVGVATTANATLSRLEVDAGNEFHEACHRIKVKE<br>EMFLYKITCIDLDPKKMTSVPMYLKKDRTIVNHYLTTVGDREAACGAN
>Selaginella-moellendorffii<br>MHACGDGALNAAGDWRYKGEGASNVVLDYRGSDSQLLGTVLRIRKAPLRENGGGCAPVLSDLERNLWKDWPAVASSSNEEELRHV<br>YVRDVMRPLLGEDYVDSGVIVPVSRKFLEDVSQHIRANRPAWRAEEADLDFSSKNVLAVCDHTLFPGVRANESDSPTLSIEIKPKWGY<br>LPQSDAISTENHVKKVVSRFAMHQLLKFVQGKIRSSSSYCPLDLFSGTRDGIIKALNALFKSPQNNLRVFKDGNRIFTGSVDGAQDSTD<br>SSASRDLLERSLVDFTDSVTGQRLDVLEKLIAEILHSSETLRKLLQTQQLDSLDIEGVIHLYYSFVGLHCPECGSDKTGCSIDSDVDKRS<br>VLFASPHSMPADEQRRNLCKFLIAATAKDCSLMVTLKPLDDSIVASSVTRSIISCPTTGRSYVYKISFLDLDLKRLDKIPHYFRLDQSIVK<br>FYAEHTT
>Capsella-rubella<br>MEEIVLEPKDAVDWSYRGEGAVNLVLAYTGSSPTFLGKILRIQKMPKDGNINGDKSENGLTTHEKLIWGDIKDLVSCQNKEIEEYLFV<br>KHVMRPLFGHKHVNPGIRLLVAKEFLEYVDSIISSQRPSWRANAASVDTNRSSVLLMDDLTLFAHGHVEDKPCLSVEIKPKCGFLPSSS<br>FIAEENVIKKSVTRFEMHQSLKFKENEITEISEYDPLDLFSGSKDRIHRAIKALYTTPQNNFRVFLNGSLVFGGLGGGICKTTSKVELAFE<br>HMLKDIIKIDDGLRADRFIELVAESVYSSGVLDQLLDVQKLDKYNIEGVIHLYYDLIDQPCKVCRELEKISKPSNQYSSMHSIPLDEKVN<br>ILKDFLISATAKDCSVMISFRSTEVGLSRSSSHSNLHLESTKQEFEYKVHFIDLDMRPLKKMEVYYKLDKKIMNTYLEMLKKKGNHQP
>Gilbertella-persicaria<br>MTPVKTSRGFLEQLASHVQPFRPDSRLKNNIVIDAPFSMLMDDLMSMLTFELKPKWGFKPSSTHPKKRLYCRYCMHSHMKQIPIHDY<br>CPLDLYSGDQARIQKALVSLFTAPLEKTLRTYVNNSPVCLEQAMFEEIRMQDILEHILVQDPILSRLKSLQSRLDALDVESILPLYQQYQ<br>SSIFHTTDIAVWQIVVKRFFNRQPLLEKEQSLQKIYEYVLSMTFKDCSIMINVNKTTEKDKRTVDVNGSLFSYDVKVIDTDLKNLEKIPY<br>WHQLDQAIVQHAVDTCFEKQECK
>Podila-epigama<br>MNTDELNVSHWTYRGEGNANVVLKYTGQAPSFHNTVLRLRKTDKTRQLTTSNISNEVEYASEVMGLLLGKEYVGQLVPVKLTQTFL<br>KDLTAVIEPLRPKDRRHRVIDLTQQCGFLALDHSMFMFHSRKYSTACVEIKPKWCFLPRPDSPFIDDSESVKKRVCRFCMYQHSKVWE<br>KKVDSVSEYCPLQLCTREPHWVRNALLGLSRSPQNNMRLFVNGVEENVARVTVSEGPGNPLVCNQDVIGQSGQAGDCQPKEGEFVG<br>EPTLVDVLTAIFTRSPILERLARLQLGLDWFDVSTIERWYRQYVARHPGTVIPEPTPKEYLAAAEAFLRRSNLNVMVGKTREEFVNHLA<br>SNTELDPLEINIPDSLMKQFFCEFLLSTTLKDCSILIAVRKVDEPLDQVESTYHSKRNDVSQVFTENIQWIEVNGRVYEYKLTFVDLDPR<br>KLNNVPKYREKDKTIVEHYKEHVASSDIKSCNLVGMILLFIGTFVYFAPAAVFVTSTLLPHCLLILGASISLVSFLGYYGVMNEKRWIL<br>WVHGMVLLIAIALQITVGAFAFAYRNQGNEVLDRSWERVFRSDPRVIQDLEHFFQCCGFEHVLDRPVPLTCAIDTRYMTGCRESIQTA<br>FQDSLQAIGVIGAVLGAIELVSLLGAAILFHRFDQQRYQREQDEGEAALVRALLEAQHVDRQIEEARRQRQNQYESLAEQVRAQTLAR<br>AQEEGLLEGGAGRRVGGGGGSQDPPPYTGGYYGSTGQTQPSSSTGRAK
>Zingiber-officinale<br>MSHHRQEEHAILRESDAKDWFYKGEGAANLVLGYSGSSPSLVGKVLRIQKSAKQINLSENGCLVLSDEERLIWRGVGELVKSTSKEA<br>AARAFVQCVMTNYLDSKHIDPGIPIPVFKEFLEAVEMNINSQRPTWRVAASNIDNLCGSALLISDHSIISGAPKSDFCFAVEIKPKCGFLP<br>SSEYIAEANSIKKHVTRFKMHQFLKLHQGEVSQVSGYDPLDLFSGSRDRMLLAITALFTSPQNNFRIFMNGALIYGGLGGGADNNSVLS<br>PKTENAIADLIAASGLQLVSLLELTAEILLRSEILHRLLATQKMDILDIEGAIHLYYNIISQPCSVCKNLSKAEVLHKYSSLHSLPWEESLK<br>IVGDYLIAATAKDCSLMISFRPSEDGHSASNHDTVFSKSSNQSYDYKAYYIDLDLKPLKKMIHYYQLDQKIVNFYKMNQEIQGNVCCS<br>GNGAPTAKH
>Raphanus-sativus<br>MEIGLEPKDAVDWSYRGEGAVNLVLAYTGSSPSFLGKMMRIQKMPNDGKEDNGNTSGNGLTSHEKVIWGECKEVVSCQNKEIVEFL<br>FVKHVMRPLLGHKHVNPGMRLLVAKEFLESVENIVTSQRPSWRADAACVDTNRNSVLLMDDLTLFAHGRVEDKPCLSVEIKPKCGFL<br>PSSSFIAQENVIKKGKTRFEMHQVLKLQENEISEISEYDPLDLFSGSKDRIHKAIRALYATPQNNFRVFLNGSLVFGGLGGGTCKTTSKV<br>EQDFEHILKDIIKTKDGPRANHFIELVAETVYTSGVLDHLLDVQKLDKYNIEGAIHVYYDLINQPCKVCKELEKSTKSSASQFSSMHLIP<br>MAEKVNVLKEFLISATAKDCSVMISFRSTNDVISRSSSHSNLHLESAKQEFDYKVHFIDLDMRPLKKMEVYYELDKKIMKTYLEMMK<br>KKEALLGGERRAQRQCF
>Danio-rerio<br>MELDKMDENDWKYHGEGNKSIVVSHLRHCQVLRLLKVPSEDSAHTRQTAEQTLRHILNIMDYSKHVMKPLLGEKYVHSGEVVGLPL<br>DFLRQMSLEVQQERPELRCDKVMDTFSGCGLCLPDLTQLPLHHLRDHRPPICVEIKPKCGFLPFSRHMTKECKWKVCRFCMHQHYKL<br>ANGKWKRLSRYCPLDLFSGSKQRMYVALKNLLEEPQNNLKIFKGGELIFSCKDDAKQQPDLNNLIQHLRPYFPHTNGLYNGHQPGKV<br>ILNEFIQVICSALLSGGDSNRSGEPRKMHLSESKPHCEASPFFPRDLIRNGHHGLPKDSVLAKILQVQMLDNLDIEGIYPLYKRVEQYLEE<br>FPKERIRLQIDGPYDESFMDTVKSCLNEDDGSVEYAIGKVHQYRVAMTAKDCSVMITFAPCEEDEEHKLNLEKPRFTYSVSILDLDTKP<br>YEGIPHQYKLDSKIVNYYLRSTQAPPPSSLYKERQECTLLFHAV
>Mus-musculus<br>MEEGKMDENEWSYHGEGNKSLVVAHAQRCVVLRFLKFPPNKKKTSEEILQHLQNIVDFGKNVMKDFLGENYVHCGEVVQLPLEFV<br>KQLCLKIQCERPESRCDKDLDTFSGYAMCLPNLTRLQTFHFAEHRPILCVEIKPKCGFIPFSNDVTHEMKHKVCRYCMHQHLKVATGK<br>WKKISKYCPLDLYSGNKQRMHFALRSLLQETQNNLRIFKNGELIYGCGDARSPVADLKELAHHLKPFFFPSNGLASGPHCTKAVIREL<br>VHVITRVLLSSSEKARAGALRLGLQGPRVCEASPFSRSLHNQGKNTSEHSGLPKGCLLYKTLQVQMLDQLDIEGLYPLYKRVEQYLEE<br>FPEERKTLQIDGPYDEVFYQKLLDLSTEDDGTVAFALTKVQQYRVAMTAKDCSIMIALSPCLQGTSSDQRPVIPSSRSRLAFSVSVLDL<br>DLKPYESIPHQYKLDSKIVNYYSKTVHAKDDTVRSTRFKEHEDCTLVLHKV
>Oryza-sativa<br>MEVVLHEGDAKDWVYKGEGAANLILSYTGSSPSMLGKVLRVKKILKDKGQPAPNCIVFSSHEEHLWGKIPGLLESVKNDCLPQAYAT<br>IVMSQHLGANHVDGGVRVRVSKNFFELAGKNVLDNRPAWRVNASAIDAGADSALLISDHTLFSGNPRGSSCIAVEIKAKCGFLPSSEY<br>ISKENSIKKQVTRYKMHQHLKFHLGEISKTSEYDPLDLFSGSKERIHMAIKSFFSTPQNNFRIFVDGSLVFGGMGGGADSVHPNETEKCL<br>EDLSKVTGLQLSDFIELLSEAIFKSGVLGKLLATQKLDDHDIEGAIHLYYNIISQPCLVCKSITDTELLRKYSTLHSLPLDKSEKIVRDFLIS<br>ATAKDCSLMISFRPRQSGTTDSEYDSVFLDSVNQSYDYKAYFIDLDVKPLDKMVHYFKLDQKIVNFYTRNGEVGGDPRDPPKGCGPR

>Agaricus-bisporus<br>MDKTQPTDWKYVSEGGATIVFSYVGPPNPAYDGTVLRLRKVTTDVEDVFTVSTTEDEPDDRIIEYQTRCMERLIPRHYLPRLESVALE<br>RRWLEQLATVHDAERPGRRIGKDHIDLTRRKGVLATDLVGGNWIAVEIKPKWAFLPSPTHLSQATKLIKTQTCRFCMHSHMRSSKDC<br>KYANKYCPLDLFSGNPHRVQQAIDDLWDAWERSDGEVNNLKIFSRGKAVRPGQAIEIVRLLHEDFITSVSPDRIQEVFKKALCDTLLQS<br>PILSIISRLQRTLDVLDIEGLSKLWREAQLESLSDEQIQVPPLGVSQSEPGMTDWVDFVTAFESVEMKELDHDQPRTSNLRYYILAYLLS<br>ATFKDCSIIARLGLLKPGNHSEIQEEQIRVIDLDPKSLNRLPRWEELDREIVMSYVPAERKMCVDENIVPSRVASGNGPES
>Phanerochaete-chrysosporium<br>MTSPLDVTTTSPRDWKYIAEGGSSIVFSYAGPPHEAFDGTALRLRKTPVSSPLVSPSPATSQVDDPDDPAIAFQREVIARLLPEEYLPRLE<br>AVQVSQEWLEELAALVEEHRPEERREKDTIDVQRRRAVLAADLVGGRVLAVEIKPKWGFLPSPAHLAPDTAPVKTTTCRFCMHAHLR<br>NGGAGDGAAEGYCPLDLFSGEPSRVRGALLALWDAWVGSSGTTNNLRVFVHGQAVRPDASPSSIQPLAAQVGLSGDFTLYTVRDAF<br>VARVLPLLLETSLLRVLANLQRSLDPLDIEGLSLLWRRHHAAQEPGVALQTQSPDDGAPAAPPLGSGLDNPDADAWKQFLAVYLAKH<br>SQMDHDHPDVADLQYYCLAYLLSATFKDCSLILRLPEEGRGSITAIDLDVKPIERLEKWERLDREIVEGYKNAWKPKQCVESKY

Table S3. FPKM of lignocellulose degradation enzyme genes in *D1* and *D10*. Expression levels of *D1* and *D10* lignocellulase genes in transcriptome sequencing.

Gene ID	*D1*/FPKM			*D10*/FPKM		
GME4803	570.904458	552.076357	580.923392	6.14514958	6.54020944	6.83394612
GME8156	121.157107	120.051654	126.387876	73.0875538	74.6770963	85.3499341
GME4792	22.798152	20.9037579	22.4809122	2.99094803	3.5117323	2.40109841
GME7109	14.94993	12.9327905	14.7093744	2.61299256	2.48575959	3.62140679
GME7110	1.40789616	1.12343899	1.42292063	2.60203884	2.54275919	2.38729899
GME4074	224.136932	216.368483	225.499204	375.815223	368.127431	461.382504
GME6111	14.6480988	17.1512199	16.576538	0.13290257	0.27138015	0.09754756
GME9857	0.90606796	0.56233508	0.38155714	0.26580514	0.27138015	0.19509512
GME6075	507.668313	490.578643	503.970495	166.734627	163.519885	84.2453774
GME3355	3.5641699	1.68239593	2.62132304	1.19285504	1.35319335	0.63232674
GME10190	13.3530872	15.5421339	15.3474236	52.8832402	50.068154	30.886729
GME507	105.559722	98.5428833	107.991467	8.92127602	7.98735836	2.88049922
GME10572	6.05867162	5.69926926	4.81237097	3.77151129	2.8149324	1.47955849
GME11091	4.63020433	4.04844644	4.56345523	2.42763945	3.55849945	1.4318308
GME261	2.17315584	1.85231534	2.32945877	0.5650741	0.26627353	0.43070394
GME10569	6.35182407	7.07911007	6.14549256	2.03277646	1.47165576	0.55950493
GME10575	1.95244891	2.83630081	1.56007544	1.10148588	1.29552593	0.63060377
GME11080	2.02309932	2.20736828	2.0516437	1.52613963	1.2020006	0.6000819
GME3357	17.7835354	17.483294	18.2463889	11.2410463	12.0241722	8.42521402
GME3356	10.0707866	10.9368588	10.7575082	9.12254144	10.7528941	6.90201149
GME10307	6.19037899	5.65095869	5.67250568	1.17677883	1.33495624	1.05566968
GME4164	0.34666122	0.03951719	0.37538641	1.13319443	0.9611685	0.57581983
GME3358	6.93981856	6.17509843	6.18129481	1.40607068	1.83034109	1.25778035
GME10233	7.81725756	8.46278372	6.83551403	9.55289753	9.66378082	2.84645894
GME12053	12.1106831	10.0354748	9.89480968	2.50518226	2.39961205	2.50738446
GME4289	18.8388403	16.1889141	17.6973248	13.4368867	12.3328899	12.9381038
GME10557	2.69742763	3.73629045	3.81498422	0.76145933	0.93291624	0.73971314
GME7667	134.651767	136.285923	140.582609	63.9261366	58.7809397	45.408389
GME4217	16.4758282	15.5373444	16.5675521	141.617681	147.665459	157.174168
GME8492	4.65896455	3.56512919	3.36047038	7.9902846	7.88308154	8.77388925
GME2405	10.6617304	11.7715086	11.5209833	8.08490639	6.72408691	6.62718183
GME5593	0	0.03683638	0	0.28439369	0.14932727	0.17891889
GME3348	809.602817	824.286353	884.763676	945.553582	931.454883	1065.26597
GME5666	0.67218457	0.59597068	0.56613153	1.44608082	0.88584657	2.2433956
GME98	1.83730448	3.50586233	3.49852876	7.66308519	7.14909698	4.56671021
GME8868	0.47312562	0.18876668	0.13282628	2.01255181	1.53044426	1.6809161
GME11585	18.2399068	13.9703302	17.6608423	20.2708523	22.4665397	19.9954826
GME6098	4.55530037	3.5743465	3.9644803	0.93544371	0.65490085	1.39771142
GME7104	9.78891733	9.61369105	9.19577035	3.18094678	2.92289764	4.30468585
GME10766	1243.27831	1309.12272	1140.01226	246.401624	236.922757	171.400136
GME11985	40.7033609	42.8548766	41.1377299	19.4535286	20.3593561	10.2980208
GME557	2.62472069	3.04641638	3.61735991	10.8498744	9.26216915	10.0186833
GME11024	14.9234119	14.3018369	14.7163388	4.15795186	2.50666641	6.9756459
GME11025	5.17444936	5.26594832	4.98965994	5.54393581	4.20473075	5.3043974
GME5668	23.7221592	25.3781669	27.8144959	5.67990259	5.57961016	5.46466986
GME11930	3.138253	2.64077942	2.40283569	1.95845559	2.09181839	1.8244726
GME5667	3.03686691	5.14948224	5.6270043	14.4993776	13.4254674	17.3610471
GME79	16.4024343	14.2908882	13.4728589	5.61097605	6.06196472	3.70649955
GME7415	0.78639492	0.7986471	0.12042227	10.8218202	11.1001871	13.6462204
GME1723	2.73050288	2.21574995	2.80641524	3.95081068	3.79248627	4.91024193
GME9665	6.53174767	6.13934294	5.51808464	18.7971869	17.391117	18.8896009
GME10881	46.5919319	44.6315032	49.0882086	76.7485851	76.7785074	75.745505
GME11891	194.194121	204.10804	208.355274	17.4121277	17.0565731	13.17814

GME1762	27.7326726	25.6636827	25.5893414	32.4341859	32.9793003	33.5921089
GME4453	3.59919722	2.40700996	2.30959132	2.23239953	2.73506632	2.72350603
GME5585	160.773772	149.497934	163.955806	55.0180422	52.4133487	62.9616327
GME5586	25.5181178	24.4347981	22.6162288	7.53209228	8.50846343	7.56983653
GME5908	16.1822211	15.1773868	17.5291096	6.17870895	6.65727123	9.60076828
GME2981	9.81765754	11.9376113	12.5999079	2.7978302	2.36922464	1.94784853
GME2076	4.26945428	4.13413816	3.83826338	2.9975371	2.48280948	2.13815082
GME8956	12.4077705	10.2816553	10.048222	3.14996353	2.26408579	0.97104162
GME2311	0.92881111	0.94959879	1.0756201	3.57626191	4.35022803	4.83731105
GME7571	21.9914456	22.3977862	22.900724	16.7263264	15.2652629	12.526375
GME3171	2.42984584	2.41513317	2.15418958	0.93792418	0.55157542	0.82609882
GME1950	7.00781172	4.69578271	4.88063515	4.42791939	3.63277824	4.26561884
GME10182	55.4744144	67.4926923	60.0589444	28.1408344	26.3778503	27.0970683
GME6933	447.855249	436.729549	446.953779	195.72391	190.638748	157.675262
GME8997	255.515185	261.22846	259.619225	574.388153	558.409052	576.039088
GME2321	67.0237665	73.1945749	63.5777724	68.5147873	64.7744769	65.8312067
GME2178	49.7946078	52.7354	53.8327243	61.8435004	56.1304602	57.4593344
GME1259	53.7724192	50.4303529	52.174998	41.3981071	41.1773195	29.593044
GME4696	31.1693302	25.4926811	27.7080312	58.3254659	58.6864355	54.2450503
GME2820	2.62472069	3.22652546	2.03137028	3.74590258	3.59500109	1.85585174
GME5711	16.7982124	15.4986434	16.8637874	9.30589226	9.29892378	12.2073803
GME5710	1.96075973	2.08613296	1.95721889	2.49257984	1.80097733	2.18132537
GME5708	52.9306555	52.6329612	58.639308	59.7073697	61.2432071	27.3693863
GME5709	0.07408486	0.35469868	0.3743773	0.65200858	1.27811295	0.86140789
GME6009	271.856435	261.501455	263.995572	438.128797	450.520363	509.068974
GME5892	167.361736	173.031691	173.979939	120.207858	131.606419	107.785619
GME5889	210.94116	200.148833	209.555457	272.771779	269.158722	306.998016
GME2937	6.36737797	8.76549899	5.93589409	6.41659238	6.07948962	4.66254109
GME5036	393.953343	398.065074	356.376939	411.799399	402.368353	334.739624
GME4972	34.4434626	32.5754775	33.6555094	10.9768964	11.1726427	12.1316818
GME10438	6.94092805	7.35371066	6.82832506	3.38796077	4.02504141	2.88229813
GME5907	6276.12812	5833.64668	5867.27562	88.5271826	89.210676	97.4095206
GME4997	245.460232	246.906662	200.474246	125.225771	124.812645	104.473315
GME3842	1924.25919	1960.41926	2080.61441	1291.10857	1251.23035	1516.03994
GME1514	3.89974912	3.91384512	3.89396085	3.99822504	3.81476162	3.311174
GME2130	3.21628794	4.2346521	3.99554244	6.61653171	6.45908501	4.71355358
GME3975	10.2295337	8.51483356	9.22374122	7.27288523	7.48310014	4.85874171
GME4680	46.2743427	44.5280084	46.8968329	3.50286973	3.77141205	2.3372993
GME6564	41.4697105	39.4791278	42.2377598	12.2285896	11.5755638	11.1748139
GME8276	3.7719496	3.39821695	3.07435666	10.8155701	10.889353	7.54540064
GME8275	18.7813347	18.14691	17.9157565	9.41442433	10.7858531	7.71438617
GME1021	3.37282949	2.92745407	3.06495041	24.2677384	21.7567342	23.8225749
GME1030	42.4876662	50.3662275	50.6459694	5.18781494	5.44795643	5.93414321
GME1020	2.03323434	1.68142876	2.21060883	1.13871921	1.23568063	0.93131423
GME7005	2.41802748	3.14726525	3.37195349	8.05874165	8.8261489	9.89023868
GME8214	5.41328162	5.60078518	6.04674651	2.48300496	2.00333434	2.33365182
GME6976	47.1055593	45.9743596	48.1456604	35.8246414	35.7767183	37.6960538
GME4617	39.9246264	41.2410522	41.4400732	28.588229	28.2303197	27.0952979
GME8025	2.57222628	2.80651109	2.78536713	2.55252045	2.75935456	2.6449324
GME9579	2.3789376	2.54234893	2.64761937	1.83195567	1.83220815	2.18156941
GME8741	163.55816	154.974601	159.055595	126.191533	135.168267	148.688843
GME8167	87.2034068	88.2114445	90.1606711	82.9607932	82.4585536	88.3810919
GME8486	0.31842365	0.45735844	0.18772935	0.28023939	0.10300217	0.24682745
GME8487	5.5769782	6.32914837	5.37554458	68.6603518	67.7507628	59.441502
GME1306	18.3730448	17.7920446	18.5629368	27.8856652	30.566288	32.6200208
GME7709	7.50281895	6.92974016	8.06054796	2.23569759	0.76440196	1.48830601
GME10260	12.7677091	13.0042824	12.5455174	14.6167799	15.1098946	19.0596634

GME7096	1.48286385	1.93361985	1.67536299	0.9549102	1.52090409	1.29663365
GME10070	3.49714206	2.49901344	3.03330701	5.3286883	5.62805417	3.79313131
GME1171	1.80490869	1.18173331	1.8709435	5.25417091	3.5429806	2.69054352
GME1172	10.5597655	10.2210508	10.8279951	11.0021407	10.8244308	9.03886247
GME4608	3.59057425	3.29183974	2.94364856	4.30298552	5.18951057	4.33032072
GME5129	8.77632463	9.24107317	9.17528921	12.962716	11.5202704	14.7706555
GME44	47.2742543	41.4846369	42.4522092	5.42820287	5.96733565	5.70544398
GME10965	23.3135288	23.5112366	21.9580778	14.6680807	15.5105769	11.3043506
GME581	91.7787628	87.1546127	89.9145637	41.6039771	38.6193104	39.1653452
GME7168	0.55792492	0.85969551	1.21525616	0.15238567	0.18669817	0.1491303
GME1784	83.4259308	86.9832046	83.6276495	57.7161739	54.692838	62.564041
GME9778	1.74743633	1.67325248	2.14453085	0.79091476	1.29200548	0.72564251
GME3094	0.84034716	0.88391993	0.90078863	0.73957564	0.53542569	0.44413546
GME7465	0.99544361	1.06665837	0.64675707	0.17521469	0.52133554	0.41336911
GME8000	6.90866536	5.83239322	7.12575	9.78119466	9.60848398	9.84712048
GME12312	0.08984374	0	0	0	0	0
GME9740	0	0	0	0.03953499	0	0
GME4774	0.89937982	0.41009451	0.72141081	1.09306427	1.33918827	1.49391018
GME7985	626.222154	656.922926	674.66257	536.837785	523.413695	428.907009
GME7015	4.89354705	3.97583155	3.70932669	8.73091586	8.15454628	10.0435015
GME10796	3.52414006	2.78051151	3.13487201	3.65911712	2.60443058	2.03346718
GME9433	6.26707673	7.67211245	7.83548427	6.88587367	7.00931221	5.88385386
GME11031	3.65779153	3.58973464	2.97445765	1.92409672	1.58667339	2.03689126
GME6726	12.8559752	13.6734296	12.3765144	13.2655995	12.8056396	10.6747964
GME5717	6.31121323	6.49349949	6.6862214	2.86473061	3.31479096	3.50441528
GME5236	2.62472069	2.09441126	2.48693494	2.23778659	2.16680085	1.62924021
GME813	7.07083847	7.9079892	7.78402262	3.96895405	3.12169405	4.26179376
GME812	1.71410331	0.51291704	0.72183146	0.4242808	0.17327187	0.20760851
GME4624	140.411378	141.139057	139.136548	86.055358	87.2078019	112.637519
GME209	28.0845114	28.3067738	29.1290225	11.5143282	10.9938781	12.148691
GME6175	19.6854052	18.7959985	20.0088441	43.1787308	39.7303516	50.570638
GME4308	50.2999752	51.1929048	52.1051374	37.4026325	39.1668284	39.6614785
GME3768	20.5308463	21.5735212	21.6746569	8.68533986	9.51189756	9.4392848
GME4803	570.904458	552.076357	580.923392	6.14514958	6.54020944	6.83394612

Table S4. Differential expression of cell physiological metabolism-related genes at *D1* and *D10*.

Gene name	id	baseMean_D10	baseMean_D1	foldChange(D1/D10)	KEGG
NADH:quinone reductase	GME10709_g	1535.423221	2732.558789	1.77967791	K03885
NDUFV1	GME10978_g	4435.574481	7902.410971	1.781598078	K03942
NDUFA8	GME9988_g	1310.424512	2202.861851	1.681029186	K03952
NDUFS2	GME4925_g	3116.802737	4816.98675	1.545489772	K03935
succinate dehydrogenase (ubiquinone) flavoprotein subunit	GME9727_g	74.09059216	375.305786	5.065498534	K09159
	GME11812_g	732.7400769	3444.02606	4.700201571	K00135
	GME1001_g	340.9786725	695.909676	2.040918486	-
QCR2	GME11737_g	3503.895067	7627.899111	2.17697704	K00415
cyt1	GME11737_g	3503.895067	7627.899111	2.17697704	K00415
PMA1	GME7606_g	132558.3936	48254.41518	0.364023838	K01535
ATP5O	GME9830_g	2644.118413	5705.680841	2.157876444	K02137
ATP5H	GME120_g	3257.1497	7275.522608	2.233708389	K02138
ATP5L	GME9085_g	1888.738617	4229.305807	2.239222394	K02140
ATP citrate (pro-S)-lyase ACLY	GME7489_g	16074.62378	5610.953477	0.349056597	K01648
CS	GME6610_g	22.56409884	71.03155668	3.147989963	K01647
	GME1237_g	486.4226165	795.2189381	1.63483134	K01647
	GME9216_g	6214.455503	9995.241668	1.608385749	K01647
	GME2255_g	2477.53794	825.9450453	0.333373319	K01647
	GME2137_g	861.5158399	214.9824596	0.249539764	K01647
SPT serine palmitoyltransferase	GME11216_g	9593.17472	26241.38231	2.735422118	K00654
	GME10325_g	4925.3399	10590.92177	2.150292567	K00654
SNF1 carbon catabolite-derepressing protein kinase	GME11216_g	9593.17472	26241.38231	2.735422118	K00654
	GME10325_g	4925.3399	10590.92177	2.150292567	K00654
	GME4766_g	3135.637372	3914.423304	1.24836607	K12761
ULK2 serine/threonine-protein kinase ULK2	GME10300_g	1647.21398	2045.731313	1.241934162	K08269
ATG13	GME2842_g	858.7691995	2300.017225	2.678271678	K08331
ATG17	GME1401_g	3656.298174	5247.558146	1.435210668	K08329
VPS15 phosphoinositide-3-kinase, regulatory subunit 4	GME11297_g	979.7354435	1332.771466	1.360338115	K08333
VPS34	GME9237_g	334.6167861	1063.163102	3.177255734	K00914
ATG5	GME7634_g	1066.813347	1358.852453	1.273749018	K08339
ATG3	GME10946_g	1027.050615	3493.16712	3.401163555	K08343
ATG4	GME10756_g	1438.914659	1932.893139	1.343299359	K08342
VAM7	GME11418_g	93.37726109	466.926893	5.000434661	K08502
VTI1	GME8251_g	1125.031749	2531.892733	2.250507807	K08493
RAB7A	GME10745_g	702.0301414	1608.263801	2.290875713	K07897
PEP4	GME7751_g	201.3651774	1509.265982	7.495168733	K01381
	GME3316_g	178.3498473	1130.211781	6.337049335	K01381
	GME93_g	32.58705036	173.060924	5.310726872	K01381
	GME6819_g	47.37143453	134.8844875	2.847380258	K01381
	GME7708_g	232.0882743	598.2388234	2.577634846	K01381
	GME7798_g	60.58372804	117.7811819	1.944105879	K01381
PRB1	GME5952_g	70.86934741	188.6584162	2.662059452	K01336
	GME6986_g	32693.87064	47422.37062	1.450497286	K01336
ACSL	GME5198_g	794.9222958	1450.800184	1.825084278	K01897
	GME706_g	766.9674234	731.7442024	0.954074684	K01897
	GME5250_g	2995.845689	2248.326884	0.750481539	K01897
	GME2998_g	274.2508163	205.7285229	0.750147349	K01897
	GME4943_g	5195.206808	3066.699239	0.590293968	K01897
	GME9195_g	6880.512487	1129.362232	0.164139261	K01897
ACOX	GME9296_g	2465.26474	5412.461784	2.195489068	K00232

	GME7743_g	126.7042047	160.3631103	1.265649476	K00232
	GME7729_g	204.3888363	118.0041819	0.577351405	K00232
	GME4948_g	842.1779948	413.030506	0.490431368	K00232
	GME7728_g	404.3277239	73.39005522	0.181511311	K00232
ACAT	GME205_g	7187.015139	3386.971162	0.471262561	K00626
ACACA	GME4326_g	6400.032945	2786.100298	0.435325931	K11262
FAS2	GME1526_g	13551.41214	6727.194928	0.496420215	K00667
	GME11343_g	1616.85599	614.5103528	0.380064988	K00667
FabG	GME12058_g	288.072849	814.5401699	2.827549256	K00059
FDFT1	GME4224_g	2352.58496	2894.290688	1.230259794	K00801
	GME3244_g	735.4515749	322.1924411	0.438087907	K00801
CYP51	GME1500_g	12313.67228	9665.761603	0.784961739	K05917
	GME4560_g	310.8056774	121.4470303	0.390749073	K05917
	GME6151_g	1453.165178	242.6782581	0.166999775	K05917
mTOR	GME4221_g	3867.335838	2936.461213	0.759298219	K07203
	GME2973_g	464.6188929	90.22350335	0.194188193	K07203

342 **Table S5. Transcription factor enrichment analysis**

gene ID	describe
GME1206_g	{IPR001138; Zn(2)-C6 fungal-type DNA-binding domain IPR007219; Transcription factor, fungi}
GME2452_g	{IPR001138; Zn(2)-C6 fungal-type DNA-binding domain IPR002409; Aflatoxin biosynthesis regulatory protein}
GME11378_g	{IPR001584; Integrase, catalytic core IPR001878; Zinc finger, CCHC-type IPR012337; Ribonuclease H-like domain IPR021109; Peptidase aspartic}
GME4066_g	{IPR002893; Zinc finger, MYND-type}
GME2529_g	{IPR001138; Zn(2)-C6 fungal-type DNA-binding domain}
GME4044_g	{IPR002893; Zinc finger, MYND-type}
GME22_g	{IPR001878; Zinc finger, CCHC-type}
GME4812_g	{IPR007087; Zinc finger, C2H2 IPR013087; Zinc finger C2H2-type/integrase DNA-binding domain IPR015880; Zinc finger, C2H2-like}
GME2136_g	{IPR007219; Transcription factor, fungi}
GME8914_g	{IPR003163; Transcription regulator HTH, APSES-type DNA-binding domain}
GME249_g	{IPR019136; Transcription factor IIIC, subunit 5}
GME5432_g	{IPR000571; Zinc finger, CCCH-type IPR011990; Tetratricopeptide-like helical IPR013026; Tetratricopeptide repeat-containing domain}
GME10056_g	{IPR002893; Zinc finger, MYND-type}
GME9978_g	{IPR001005; SANT/Myb domain IPR009057; Homeodomain-like IPR017877; Myb-like domain IPR017930; Myb domain}
GME5436_g	{IPR017956; AT hook, DNA-binding motif IPR020478; AT hook-like}
GME2672_g	{IPR001138; Zn(2)-C6 fungal-type DNA-binding domain IPR002409; Aflatoxin biosynthesis regulatory protein}
GME4004_g	{IPR001607; Zinc finger, UBP-type IPR001841; Zinc finger, RING-type IPR011422; BRCA1-associated 2 IPR013083; Zinc finger, RING/FYVE/PHD-type}
GME2135_g	{IPR001138; Zn(2)-C6 fungal-type DNA-binding domain}
GME7839_g	{IPR001878; Zinc finger, CCHC-type}
GME10703_g	{IPR007087; Zinc finger, C2H2}
GME578_g	{IPR001841; Zinc finger, RING-type IPR013083; Zinc finger, RING/FYVE/PHD-type IPR017907; Zinc finger, RING-type, conserved site}
GME10797_g	{IPR011991; Winged helix-turn-helix transcription repressor DNA-binding IPR014048; Methylated-DNA-[protein]-cysteine S-methyltransferase, DNA binding}
GME7591_g	{IPR003265; HhH-GPD domain IPR011257; DNA glycosylase IPR023170; Helix-turn-helix, base-excision DNA repair, C-terminal}
GME9102_g	{IPR000571; Zinc finger, CCCH-type}
GME5389_g	{IPR007087; Zinc finger, C2H2 IPR011146; HIT-like domain}
GME12114_g	{IPR002893; Zinc finger, MYND-type}

GME10636_g	{IPR007872; Zinc finger, DPH-type}
GME6645_g	{IPR002893; Zinc finger, MYND-type}
GME3328_g	{IPR001138; Zn(2)-C6 fungal-type DNA-binding domain IPR021858; Protein of unknown function DUF3468}
GME12003_g	{IPR000637; HMG-I/HMG-Y, DNA-binding, conserved site IPR001138; Zn(2)-C6 fungal-type DNA-binding domain}
GME5427_g	{IPR000571; Zinc finger, CCCH-type IPR001440; Tetratricopeptide TPR-1 IPR011990; Tetratricopeptide-like helical IPR013026; Tetratricopeptide repeat-containing domain IPR013105; Tetratricopeptide TPR2 IPR019734; Tetratricopeptide repeat}
GME4800_g	{IPR001138; Zn(2)-C6 fungal-type DNA-binding domain}
GME3071_g	{IPR002893; Zinc finger, MYND-type IPR011989; Armadillo-like helical}
GME4045_g	{IPR002893; Zinc finger, MYND-type}
GME11686_g	{IPR001841; Zinc finger, RING-type IPR013083; Zinc finger, RING/FYVE/PHD-type}
GME989_g	{IPR001841; Zinc finger, RING-type IPR013083; Zinc finger, RING/FYVE/PHD-type IPR017907; Zinc finger, RING-type, conserved site}
GME999_g	{IPR003613; U box domain IPR013083; Zinc finger, RING/FYVE/PHD-type}
GME3831_g	{IPR000270; Phox/Bem1p IPR000433; Zinc finger, ZZ-type}
GME6531_g	{IPR001193; Peptidase M50, mammalian sterol-regulatory element binding protein}
GME5812_g	{IPR006565; Bromodomain transcription factor}
GME612_g	{IPR000591; DEP domain IPR006992; Amidohydrolase 2 IPR011991; Winged helix-turn-helix transcription repressor DNA-binding}
GME3864_g	{IPR011990; Tetratricopeptide-like helical IPR013026; Tetratricopeptide repeat-containing domain IPR019734; Tetratricopeptide repeat}
GME7383_g	{IPR006572; Zinc finger, DBF-type IPR013939; Regulatory subunit Dfp1/Him1, central region}
GME8417_g	{IPR003701; DNA repair protein Mre11 IPR004843; Metallophosphoesterase domain IPR007281; Mre11, DNA-binding}
GME7410_g	{IPR001737; Ribosomal RNA adenine methylase transferase IPR023165; rRNA adenine dimethylase-like}
GME2198_g	{IPR003593; AAA+ ATPase domain IPR010995; DNA repair Rad51/transcription factor NusA, alpha-helical IPR011941; DNA recombination/repair protein Rad51 IPR013632; DNA recombination and repair protein Rad51, C-terminal IPR016467; DNA recombination and repair protein, RecA-like IPR020587; DNA recombination/repair protein RecA, monomer-monomer interface IPR020588; DNA recombination/repair protein RecA/RadB, ATP-binding domain}
GME3744_g	{IPR007087; Zinc finger, C2H2 IPR015880; Zinc finger, C2H2-like}
GME2676_g	{IPR001841; Zinc finger, RING-type IPR013083; Zinc finger, RING/FYVE/PHD-type}
GME7779_g	{IPR001841; Zinc finger, RING-type IPR013083; Zinc finger, RING/FYVE/PHD-type IPR017907; Zinc finger, RING-type, conserved site}

GME8051_g	{IPR001138; Zn(2)-C6 fungal-type DNA-binding domain IPR007219; Transcription factor, fungi}
GME1428_g	{IPR001965; Zinc finger, PHD-type IPR002717; MOZ/SAS-like protein IPR011011; Zinc finger, FYVE/PHD-type IPR013083; Zinc finger, RING/FYVE/PHD-type IPR016181; Acyl-CoA N-acyltransferase IPR019787; Zinc finger, PHD-finger}
GME556_g	{IPR001584; Integrase, catalytic core IPR001878; Zinc finger, CCHC-type IPR012337; Ribonuclease H-like domain IPR013103; Reverse transcriptase, RNA-dependent DNA polymerase}
GME4042_g	{IPR002893; Zinc finger, MYND-type}
GME11640_g	{IPR001841; Zinc finger, RING-type IPR013083; Zinc finger, RING/FYVE/PHD-type IPR017907; Zinc finger, RING-type, conserved site}
GME6512_g	{IPR001841; Zinc finger, RING-type IPR004331; SPX, N-terminal IPR013083; Zinc finger, RING/FYVE/PHD-type IPR017907; Zinc finger, RING-type, conserved site}
GME11881_g	{IPR001841; Zinc finger, RING-type IPR013083; Zinc finger, RING/FYVE/PHD-type IPR024766; Zinc finger, RING-H2-type}
GME1081_g	{IPR002893; Zinc finger, MYND-type}
GME10882_g	{IPR000467; G-patch domain IPR000504; RNA recognition motif domain IPR007087; Zinc finger, C2H2 IPR012677; Nucleotide-binding, alpha-beta plait}
GME149_g	{IPR002893; Zinc finger, MYND-type IPR011990; Tetratricopeptide-like helical}
GME3538_g	{IPR000306; Zinc finger, FYVE-type IPR002110; Ankyrin repeat IPR011011; Zinc finger, FYVE/PHD-type IPR013083; Zinc finger, RING/FYVE/PHD-type IPR017455; Zinc finger, FYVE-related IPR020683; Ankyrin repeat-containing domain}
GME357_g	{IPR001841; Zinc finger, RING-type IPR006845; Pex, N-terminal IPR013083; Zinc finger, RING/FYVE/PHD-type IPR017907; Zinc finger, RING-type, conserved site}
GME1906_g	{IPR001680; WD40 repeat IPR003613; U box domain IPR013083; Zinc finger, RING/FYVE/PHD-type IPR013915; Pre-mRNA-splicing factor 19 IPR015943; WD40/YVTN repeat-like-containing domain IPR017986; WD40-repeat-containing domain}
GME1606_g	{IPR001594; Zinc finger, DHHC-type, palmitoyltransferase IPR013218; Mtw1 kinetochore complex, DSN1}
GME122_g	{IPR007219; Transcription factor, fungi}
GME4837_g	{IPR000994; Peptidase M24, structural domain IPR001714; Peptidase M24, methionine aminopeptidase IPR002168; Lipase, GDXG, active site IPR002468; Peptidase M24A, methionine aminopeptidase, subfamily 2 IPR011991; Winged helix-turn-helix transcription repressor DNA-binding IPR013094; Alpha/beta hydrolase fold-3}
GME1496_g	{IPR012935; Zinc finger, C3HC-like IPR013909; Nuclear-interacting partner of ALK/Rsm1-like}
GME3408_g	{IPR011011; Zinc finger, FYVE/PHD-type IPR024610; Inhibitor of growth protein, N-terminal}
GME4229_g	{IPR001841; Zinc finger, RING-type IPR013083; Zinc finger, RING/FYVE/PHD-type IPR017907; Zinc finger, RING-type, conserved site}
GME8637_g	{IPR000504; RNA recognition motif domain IPR001841; Zinc finger, RING-type IPR003954; RNA

	recognition motif domain, eukaryote IPR012677; Nucleotide-binding, alpha-beta plait IPR013083; Zinc finger, RING/FYVE/PHD-type}
GME3926_g	{IPR000571; Zinc finger, CCCH-type IPR001841; Zinc finger, RING-type IPR013083; Zinc finger, RING/FYVE/PHD-type IPR017907; Zinc finger, RING-type, conserved site IPR026290; Putative E3 ubiquitin-protein ligase, makorin-related}
GME1246_g	{IPR001044; Xeroderma pigmentosum group G protein IPR006084; DNA repair protein (XPGC)/yeast Rad IPR006085; XPG N-terminal IPR006086; XPG/RAD2 endonuclease IPR008918; Helix-hairpin-helix motif, class 2 IPR019974; XPG conserved site IPR020045; 5'-3' exonuclease, C-terminal domain}
GME9863_g	{IPR000938; CAP Gly-rich domain IPR001878; Zinc finger, CCHC-type IPR009053; Prefoldin}
GME10919_g	{IPR001965; Zinc finger, PHD-type IPR011011; Zinc finger, FYVE/PHD-type IPR013083; Zinc finger, RING/FYVE/PHD-type IPR019787; Zinc finger, PHD-finger}
GME8421_g	{IPR001138; Zn(2)-C6 fungal-type DNA-binding domain IPR007219; Transcription factor, fungi}
GME8263_g	{IPR001965; Zinc finger, PHD-type IPR011011; Zinc finger, FYVE/PHD-type IPR013083; Zinc finger, RING/FYVE/PHD-type IPR019786; Zinc finger, PHD-type, conserved site IPR019787; Zinc finger, PHD-finger}
GME3510_g	{IPR003604; Zinc finger, U1-type IPR021966; Splicing factor SF3a60 binding domain IPR024598; Domain of unknown function DUF3449}
GME6013_g	{IPR000571; Zinc finger, CCCH-type}
GME4740_g	{IPR001965; Zinc finger, PHD-type IPR011011; Zinc finger, FYVE/PHD-type IPR013083; Zinc finger, RING/FYVE/PHD-type IPR019786; Zinc finger, PHD-type, conserved site IPR019787; Zinc finger, PHD-finger IPR024610; Inhibitor of growth protein, N-terminal}
GME9871_g	{IPR000571; Zinc finger, CCCH-type IPR001841; Zinc finger, RING-type IPR013083; Zinc finger, RING/FYVE/PHD-type IPR017907; Zinc finger, RING-type, conserved site}
GME2453_g	{IPR000014; PAS domain IPR000679; Zinc finger, GATA-type IPR013088; Zinc finger, NHR/GATA-type}
GME4301_g	{IPR007219; Transcription factor, fungi}
GME8052_g	{IPR007219; Transcription factor, fungi}
GME4580_g	{IPR007087; Zinc finger, C2H2 IPR013087; Zinc finger C2H2-type/integrase DNA-binding domain IPR015880; Zinc finger, C2H2-like}
GME9026_g	{IPR017923; Transcription factor IIS, N-terminal}
GME4656_g	{IPR001138; Zn(2)-C6 fungal-type DNA-binding domain IPR007219; Transcription factor, fungi}
GME5528_g	{IPR003347; JmjC domain IPR003349; Transcription factor jumonji, JmjN}
GME2343_g	{IPR007087; Zinc finger, C2H2 IPR013087; Zinc finger C2H2-type/integrase DNA-binding domain IPR015880; Zinc finger, C2H2-like}
GME7812_g	{IPR001138; Zn(2)-C6 fungal-type DNA-binding domain IPR007219; Transcription factor, fungi}

343
